# Trauma-Informed Care for Acute Care Settings: A Novel Simulation Training for Medical Students

**DOI:** 10.15766/mep_2374-8265.11327

**Published:** 2023-07-28

**Authors:** Caroline H. Lee, Carlos Dos Santos, Taylor Brown, Henry Ashworth, Jason J. Lewis

**Affiliations:** 1 Fourth-Year Medical Student, Harvard Medical School; 2 Second-Year Resident, Department of Emergency Medicine, Beth Israel Deaconess Medical Center; 3 First-Year Resident, Department of Emergency Medicine, Highland Hospital, Alameda Health System; 4 Assistant Professor, Department of Emergency Medicine, Beth Israel Deaconess Medical Center

**Keywords:** Trauma-Informed Care, Communication Skills, Emergency Medicine, Gender Issues in Medicine, Intimate Partner Violence, Pediatric Emergency Medicine, Simulation, Diversity, Equity, Inclusion

## Abstract

**Introduction:**

Physicians often care for patients who have experienced traumatic events including abuse, discrimination, and violence. Trauma-informed care (TIC) is a framework that recognizes the prevalence of trauma, promotes patient empowerment, and minimizes retraumatization. There are limited education curricula on how to apply TIC to acute care settings, with simulation-based training presenting a novel educational tool for this aim.

**Methods:**

Students participated in a didactic on TIC principles and its applications in acute care settings. Learners participated in three simulation cases where they performed physical exams and gathered history on patients with urgent medical needs related to intimate partner violence, transgender health, and health care discrimination. Debriefing followed each simulation.

**Results:**

Seventeen medical students participated across four sessions. The sessions were evaluated with pre- and postparticipation surveys, including Likert scales and free-response questions. After participation, individuals' self-assessed confidence improved across multiple domains, including identifying situations for trauma screenings, inquiring about trauma, and responding as a bystander. Learners also felt more familiar with TIC-specific history taking and physical exam skills. Finally, simulation was perceived as a beneficial educational tool. All findings were statistically significant (*p* ≤ .01).

**Discussion:**

Our simulation-based training enabled students to practice conversations and interventions related to trauma. This novel training represents a feasible and effective means for teaching TIC for acute care settings, including in the emergency department and in-patient settings. Development and evaluation were supported by the Society for Academic Emergency Medicine.

## Educational Objectives

By the end of this session, learners will be able to:

1. Identify the importance of incorporating trauma-informed care (TIC) into clinical practice as a universal precaution in the acute care setting.
2. Identify clinical situations in which performing safety screenings for trauma history is indicated in the acute care setting.
3. Improve comfort in using TIC principles during a physical exam in an acute care setting.
4. Increase confidence when responding to colleagues through bystander intervention using TIC principles.

## Introduction

Physicians often care for patients who have experienced trauma, which is defined as experiences or events that have lasting adverse effects on one's mental, physical, social, or spiritual well-being.^1,2^ Patients who have experienced trauma or adverse experiences have higher rates of mental health conditions and chronic physical symptoms.^3,4^ Further retraumatization may occur during medical encounters, leading to distrust and decreased usage of essential services, including preventative screenings.^3,4^ Avoidance of preventative health care may in turn also lead to increased utilization of the emergency department (ED).^3–6^

Trauma-informed care (TIC) is a framework that describes how providers can reduce retraumatization and promote healing for their patients.^2^ This approach is based on the following six key principles: (1) safety; (2) trustworthiness and transparency; (3) peer support; (4) collaboration and mutuality; (5) empowerment, voice, and choice; and (6) cultural, historical, and gender Issues.^2^ Integrating TIC into clinical care has led to increased perceived partnership between patients and physicians, which may improve patient engagement and health outcomes.^7,8^

Acute care settings, including the ED and urgent care, treat millions of patients per year who have had exposure to trauma, including experiences of abuse, discrimination, and violence.^2,9^ These settings also have high volume and turnover, with providers caring for patients experiencing high levels of stress and/or acute pain. An understanding of TIC is essential when practicing in these settings, as patients may present with signs, symptoms, and/or sequelae of trauma (e.g., intimate partner violence [IPV], elder or child abuse, or community violence). Additionally, adults who frequently present to the ED have high rates of post-traumatic stress disorder and adverse childhood experiences (ACEs), suggesting this patient population has greater prevalence of preexisting adversity.^9–11^ The application of TIC may also improve aspects of patient-centered care and safety, such as by reducing restraint usage in the ED.^12–15^

Despite the significant need for the implementation of TIC in acute care settings, several barriers for acute care providers exist, including a lack of longitudinal relationships with patients, limited training, high acuity, and time constraints.^16,17^ Due to the lack of continuity of care, clinicians must also be specifically trained on situations when performing a trauma-informed safety screening is warranted for patient safety.

Educational trainings for health care workers can improve their understanding of TIC and implementation of TIC principles.^13,17^ In particular, incorporating these trainings during undergraduate and graduate medical education can provide developing physicians with an important framework to apply to their future patient encounters.^18^ Undergraduate medical education in TIC, as described in *MedEdPORTAL,* has utilized tools including small-group discussions, peer role-play, and standardized patient demonstrations.^18–22^

Our educational workshop focuses on integrating TIC into acute care settings using simulations, which are designed to reproduce complex clinical encounters in a controlled and safe environment.^23,24^ Simulations are particularly helpful for recreating high-risk situations with sensitive topics and vulnerable patient populations, thus making them particularly well suited for educating learners about TIC.^23,24^

This workshop describes a novel application to TIC to educate medical students on how to apply their skills to the acute care setting through simulation-based training. Simulations of trauma-informed approaches have previously been developed on specific topics such as applications to human trafficking and approaching ACEs in the pediatric population.^21,25,26^ To our knowledge, this publication is the first of its kind to describe a simulation training to educate medical students on TIC practices in acute care settings.

## Methods

### Development

We developed a simulation workshop on TIC practices in acute care settings building off previous literature on TIC workshops.^17–20^ We recruited multidisciplinary TIC experts, including physicians, researchers, and nurses, from Brigham and Women's Hospital, Harvard Medical School, and Beth Israel Deaconess Medical Center to review the content of the didactic presentation and simulation cases. Cases were based on real-life patient cases experienced in the ED using input from emergency medicine residents and attendings, then reviewed by the experts mentioned above for content accuracy and patient representation. The workshop was part of a larger educational movement at Harvard to longitudinally incorporate TIC into undergraduate medical education.^27,28^

Two authors (Caroline H. Lee and Carlos Dos Santos) had participated as learners and one author (Taylor Brown) helped develop the TIC curriculum for first-year medical students at Harvard Medical School that focusing on applying TIC history taking and physical exam skills.^18,20^ Based on these experiences, we identified gaps in the curriculum and developed our educational materials to focus on knowledge not covered in the existing curriculum, such as bystander training and trauma-informed safety screening in acute care settings.

The session consisted of a didactic lecture and three simulation cases with patients who had urgent medical needs and a relevant history of traumatic experiences. Facilitators first presented a didactic that covered an introduction to TIC, unique challenges of delivering TIC in acute care settings, and applications to history taking and physical exam procedures (Appendix A). The didactic included opportunities for active learning through prompts for reflection and open-ended discussion. Learners then interacted with three cases: (1) a patient presenting with physical sequelae from IPV, (2) a transgender patient with postoperative complications following a gender-affirming surgical procedure, and (3) a patient with IV drug use and history of stigmatizing experiences by medical providers.

In accordance with trauma-informed medical education, we incorporated TIC principles into the delivery of the workshop to ensure that students were supported while engaging with challenging subject matter.^28^ At the beginning of the didactic and the start of simulation cases, facilitators provided content warnings about sensitive topics, reminders to practice self-care, and invitations to take breaks from the workshop if needed. We also provided institution-specific self-care resources. Finally, the facilitators acted as the patients, so students did not have to embody the lived experiences of trauma. Instead, students interacted with the simulated patients in patient care roles similar to those in which they would encounter such individuals in real clinical settings.

The simulation sessions were performed in the Shapiro Simulation and Skills Center at Beth Israel Deaconess Medical Center in a simulated ED. We recruited first- through fourth-year medical students from Harvard Medical School and the University of Massachusetts Chan Medical School via email invitation.

We provided students with a $10 gift card as compensation for their participation. Implementation of the sessions was supported by a grant from the Society for Academic Emergency Medicine.

### Equipment

Equipment used to implement the three simulation cases included the following:

• High-fidelity mannequin (with vaginal external genitalia)
• Hospital bed
• Fake blood to simulate hemorrhage
• IV, IV pole, normal saline bag and tubing, syringes, and needles
• Blood pressure cuff
• Screen to display vital signs (heart rate, blood pressure, respiratory rate), with sound and alarms on

### Personnel

Facilitators (upper-year medical students and emergency medicine residents) and faculty mentors were chosen due to their preexisting familiarity with TIC. One faculty mentor had content expertise in emergency medicine and medical student education, and another faculty mentor had content expertise in TIC. All facilitators received the didactic, case templates, and debriefing materials (Appendices A–C) beforehand to review in preparation.

At least two facilitators (upper-year medical student or resident) played the following roles:

• Case 1: IPV
  ○ Simulated participant: patient
  ○ Simulated participant: patient's partner
• Case 2: transgender patient
  ○ Simulated participant: patient
  ○ Simulated participant: health care colleague (nurse, medical assistant)
• Case 3: health care discrimination
  ○ Simulated participant: patient
  ○ Simulated participant: resident or attending

Faculty mentors provided feedback for the learners during the debriefing session. A simulation technician was responsible for changing vital signs and setting up the simulation environment.

### Implementation

We hosted four sessions, with three to five learners per session. Three of these sessions were optional learning activities open to all medical students, and one session was integrated into an Emergency Medicine Bootcamp course for graduating medical students.

Each session lasted approximately 2.5 hours. The workshop began with a 1-hour interactive didactic followed by three simulation cases. Each case took 30 minutes including the simulation and debrief.

Prior to each simulation, participants self-designated themselves to at least one of the following roles: team leader, history taker, physical examiner, communicator with other health care staff, and oral presenter. Roles rotated with each simulation.

Before each case, a facilitator established the setting as the ED, introduced the one-liner about the patient, and asked the team to gather a history and perform an exam on the patient. Participants were not alerted to the specific type of adversity the patient had experienced. When participants entered the room, vital signs were already provided to the team via simulation monitors. A summary of the case scenarios appears below and is described further in the case templates (Appendix B).

#### Case 1

The patient was introduced as a male presenting with unilateral arm pain with a history of prior fractures. Learners encountered the patient and his partner in the examining room. When individuals asked the patient questions, his partner periodically answered for the patient and interjected into the conversation. The history provided to explain the injury was vague and included changing details. If participants separated the partner and patient to speak to the patient alone, the patient revealed a history of IPV and fear regarding his safety at home. This situation provided participants with the opportunity to practice removing potential suspects for confidential conversations and to identify that a trauma-informed safety screen focused on IPV was indicated.

#### Case 2

The patient was introduced as a female presenting with light-headedness. Participants encountered a mannequin (voiced by a facilitator) that was hypotensive and tachycardic. She revealed that she had postsurgical bleeding after a vaginoplasty. During the case, another health care worker misgendered the patient. This situation offered learners the opportunity to address potential conflict in a health care team, to practice conversations related to transgender health, and to perform a trauma-informed physical exam.

#### Case 3

The patient was introduced as a male with right arm pain. While he originally explained his pain as caused by an animal bite, he started to exhibit signs of opioid withdrawal, including nausea and restlessness, and later revealed that he used IV drugs. During the assessment, the facilitator, acting as a supervising physician, made statements referring to a history of frequent ED visits and implying that the patient was malingering, which the patient overheard. This situation gave participants the chance to practice establishing trust and rapport with patients who may have distrust related to prior experiences in the health care system and to manage comments from other health care providers.

During each case, the facilitator completed a skills checklist of TIC actions students implemented during their SIM (Appendix D). At the conclusion of each scenario, students provided an oral summary of patient history, assessment, and plan to the facilitator.

### Assessment

The facilitator completed the simulation checklists (Appendix D) to record TIC-relevant actions that students implemented during each scenario. Facilitators used the checklists to guide feedback provided during each debriefing session. We created the checklists to assess completion of principles from prior TIC curricula on history taking and physical exam skills, as well as tasks specific to each case.^18,20^

We distributed pre- and posttraining survey questionnaires (Appendix E) to participants. The questionnaires used Likert-scale, multiple-choice, and free-response questions. Each learner answered questions that evaluated self-perceived confidence and familiarity with TIC skills using a 5-point Likert scale (1 = *not at all confident/familiar,* 5 = *extremely confident/familiar*), utility of simulations to teach TIC using a 5-point Likert scale (1 = *not at all useful,* 5 = *extremely useful*), multiple-choice knowledge questions, and free-response questions about major takeaways and suggestions for improvement. We designed these evaluations to assess reaction and learning in the Kirkpatrick model.^29^ We developed questions based on a literature review of evaluations for prior simulation and TIC curricula.^18,20–22,26^ We then tailored questions to our specific educational objectives and solicited feedback from content experts in both medical education and TIC.

We analyzed the survey results using Microsoft Excel. A Fisher exact test was used to compare pre- and postparticipation answers. Free responses were analyzed for recurrent themes.

### Debriefing

Given the difficult interactions and sensitive topics simulated in the sessions, a dedicated debriefing session after each case was facilitated to allow for participant reflection.

Following each case, students met with facilitators to discuss reactions to the case in a roundtable and guided-discussion format. Students were first asked to self-assess their performance by discussing what they perceived had gone well and identifying areas for improvement. Facilitators then provided feedback on the students' interactions with the patient and other individuals in the simulation and suggested areas of improvement based on the TIC skills checklist (Appendix D).

Finally, facilitators summarized the following teaching points relevant to each case, as listed in the debriefing materials (Appendix C):

• Case 1: methods of talking privately to patients suspected of being victims of violence, de-escalation strategies, and next steps for ensuring patient safety
• Case 2: balancing the need for acute medical management and TIC principles, TIC applications to invasive physical exams, and bystander responses to misgendering
• Case 3: discrimination in the health care system, safety for individuals who use injection drugs, and engagement of other team members using stigmatizing language

## Results

Seventeen medical students participated in the training, all of whom completed the pre- and postsimulation surveys. Of all the learners, 65% had not yet completed their core clinical year and 35% had. Most participants had had some form of prior education in medical school on obtaining a history (88%) and performing a physical exam (76%) using TIC principles.

Students reacted unanimously positively to the session, with 100% rating it as somewhat or extremely effective in meeting the stated learning objectives.

Students' self-assessed confidence in relevant TIC knowledge improved after participation in the workshop. They reported improved confidence in identifying situations where a trauma-informed safety screening was indicated in acute care settings (*p* ≤ .01), sensitively inquiring about trauma (*p* ≤ .01), and responding as a bystander after observing breaches in TIC (*p* ≤ .01; Table 1). Learners also felt more familiar with specific skills, including TIC-informed history taking (*p* ≤ .01) and performing TIC-informed physical exams (*p* ≤ .01; Table 1).

**Table 1.**
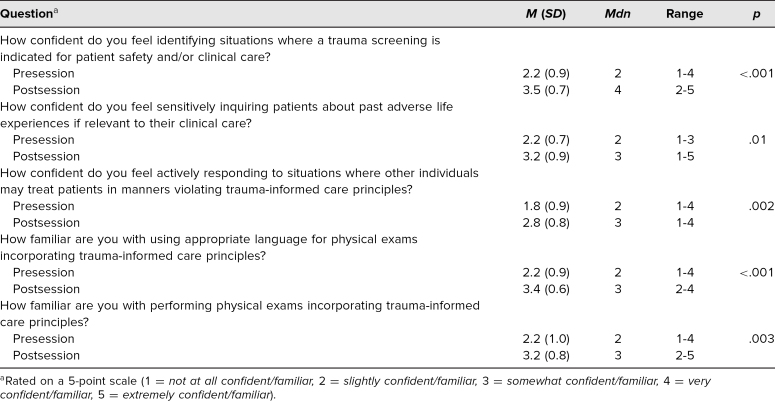
Confidence and Familiarity With Trauma-Informed Care Before and After Participation (*N* = 17)

The utility of simulation-based exercises as a mechanism for learning about TIC and preparing for patient interactions was perceived as more useful after participation in the workshop (*p* ≤ .01; Table 2). Students also expressed greater interest in having more simulations integrated into their medical school curriculum after their experience (*p* ≤ .01; Table 2).

**Table 2.**
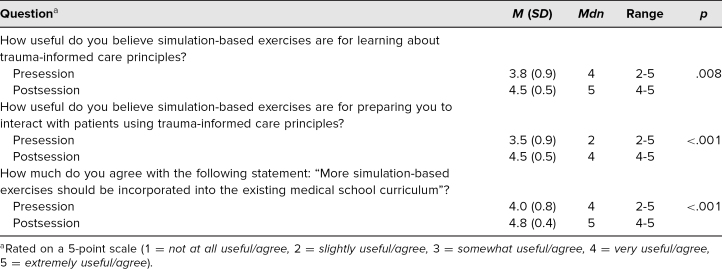
Utility of Simulations in Learning Trauma-Informed Care (*N* = 17)

The differences in learners' performance on knowledge questions were variable (Table 3). After participation, there were improvements in the number of correct responses to questions where students were asked to determine whether all scenarios were applicable to TIC and to identify situations in which to elicit further information after a patient's disclosure of trauma. There was no change in performance on questions about the Substance Abuse and Mental Health Services Administration's six TIC principles and what percentage of individuals had experienced at least one ACE in their life. There was a slight decrease in the number of correct answers on a scenario-based question on the best course of action as a bystander.

**Table 3.**
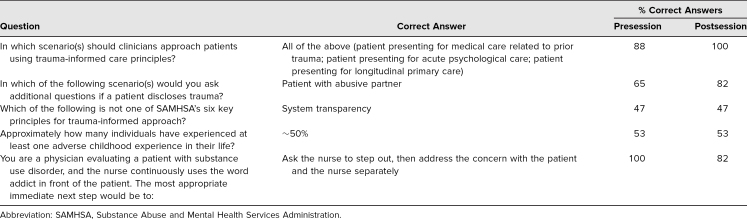
Results of Knowledge-Based Assessment Questions (*N* = 17)

Themes from the students' free-response questions are summarized in Table 4. Students expressed the goals of their participation in the workshop as being a desire to practice TIC skills, respond appropriately to patient disclosures of trauma, and understand TIC physical exam techniques. Challenges in medical school in learning about TIC included a lack of familiarity with the topic by their clinical preceptors, limited integration into the core curriculum, and difficulties in universally applying the principles. After the session, learners found that the opportunity to practice TIC in a supportive setting and the debriefing sessions were the most useful components of the workshop. Participants suggested areas of improvement such as having increased case difficulty for students of different clinical skill levels and a lower learner-to-facilitator ratio. Furthermore, major takeaways from the session included knowledge of specific TIC terminology to use with patients and, importantly, a desire to universally provide TIC to all their patients.

**Table 4.**
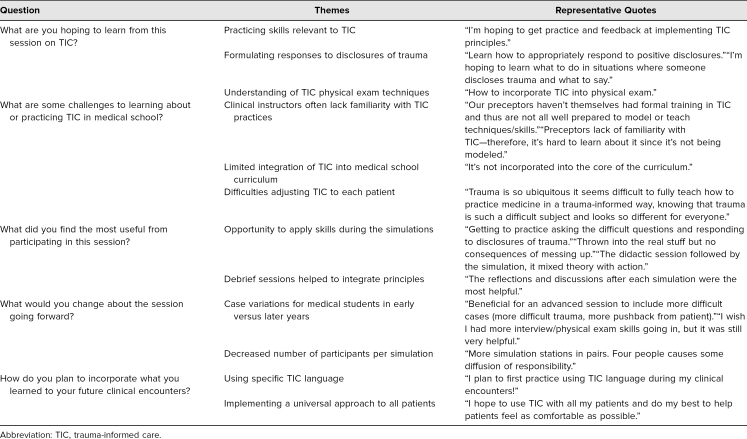
Themes From Free-Response Questions (*N* = 17)

## Discussion

Our educational intervention represents the first simulation-based one focused on applications of TIC to acute care settings. Prior research has demonstrated the value of using simulations to practice TIC.^25,26^ However, those situations were limited in scope and did not reflect the unique challenges present in acute care environments. It is essential to teach medical students how to integrate TIC principles into acute care settings, given the need to treat a patient population directly impacted by sequelae of trauma, to intervene to ensure patient safety, and to navigate unique challenges including time constraints and lack of privacy. Our novel simulation training offers students an opportunity to practice integrating TIC principles into acute care situations, preparing them to provide holistic care for a diversity of patients.

The workshop's measured outcomes demonstrated that simulating acute care settings to teach TIC was feasible, well received, and effective. Students' confidence and familiarity in specific TIC skills, from inquiring about adverse life experiences to actively responding as a bystander, significantly improved after their participation. Simulations were perceived as a valuable modality for education on TIC, and students expressed the desire for further simulation training during medical school. One of the 17 participants indicated feeling “not at all comfortable” with some TIC skills after the session, illustrating the need for this workshop to be part of ongoing longitudinally integrated TIC curricula. Overall, the workshop was successful in facilitating practice of TIC clinical skills, including history taking and performing physical exams.

There were limited improvements in the knowledge assessment questions, which may represent the fact that the answers were not explicitly highlighted in the training and that inherent ambiguity exists in answers to scenario-based questions. However, after participating in the session, all students were able to correctly identify TIC as an important universal precaution in all clinical scenarios. Additionally, this exercise was performed among students with high levels of exposure to TIC even before participation, due to the incorporation of TIC into the first-year curriculum at Harvard Medical School.^18^ Greater knowledge gains may be observed in groups with less extensive backgrounds in TIC.

There are several limitations to our curricular evaluation. First, most of our participants had some prior exposure to TIC during their medical school curriculum. Thus, our findings can be largely applied to students with a baseline understanding of TIC, and it is unknown if the intervention would have the same effect in populations with more limited prior exposure. The sample size was too small to compare differences between individuals who participated before and after their core clinical experiences. Additionally, because participants volunteered their time to participate, they already demonstrated interest in the topic of TIC and may have been comfortable with role-playing in the training. Next, postparticipation surveys were completed immediately after students finished the simulations. This methodology could not evaluate the effectiveness of this intervention after longer follow-up to see if the intervention led to long-term knowledge retention or increased application of TIC principles in clinical settings.

Some logistical challenges we encountered included the time and resources required to implement this training. While all our facilitators and faculty mentors had preexisting education on TIC practices, additional faculty development on TIC may need to be created and evaluated if knowledgeable facilitators are not readily available. Options to reduce the training from 2.5 hours due to limited time constraints include providing the didactic online asynchronously and/or selecting one or two simulation cases to implement instead of three. Participants provided feedback that the session would have been improved if there were even smaller groups for the simulations. Therefore, this intervention may be difficult to incorporate with fewer resources or larger groups. Despite these limitations, our simulation still provides a unique method to educate students on how to integrate TIC into clinical acute care environments.

The medical students who participated in our sessions noted that a key challenge to practicing TIC was that their preceptors often lacked familiarity with TIC practices. Therefore, future simulations teaching TIC should focus on utility in educating both residents and attending physicians who serve as clinicians and educational role models for trainees. Additionally, further research is needed to understand if simulation-based TIC workshops lead to retained knowledge and subsequent integration of learned skills into clinical care. These aims can be achieved by means of longitudinal evaluations that incorporate follow-up with students or clinical skills assessments through individual OSCE performances.

Overall, this novel training represents a well-received and effective means for teaching TIC skills for acute care environments.

## Appendices


TIC Acute Care Didactic.pptxSimulation Cases.docxDebriefing Materials.docxSimulation Checklists.docxSurvey Questions.docx
*All appendices are peer reviewed as integral parts of the Original Publication.*
