# Supplementary material for: Trauma-Informed Care for Acute Care Settings: A Novel Simulation Training for Medical Students
Source: MedEdPORTAL. 2023 Jul 28;19:11327. doi: 10.15766/mep_2374-8265.11327 (PMC10376910; doi:10.15766/mep_2374-8265.11327)
Supplement: Supplementary file 1 — TIC Acute Care Didactic.pptxSimulation Cases.docxDebriefing Materials.docxSimulation Checklists.docxSurvey Questions.docx [file mep_2374-8265.11327-s001.zip › B. Simulation Cases.docx]

**Trauma-Informed Care in Acute Care Settings:**

**Simulation Cases**

| **Simulation 1**    **SIMULATION CASE TITLE: Intimate Partner Violence Case**    **LEARNER AUDIENCE: Medical Students** | |
| --- | --- |
| **PATIENT NAME: Skyler Smith**    **PATIENT AGE: 26**    **CHIEF COMPLAINT: L arm pain**    **PHYSICAL SETTING: Emergency Department** | |
|  | |
| **Brief narrative description of case** | *26 y/o M presents with L arm pain, secondary to intimate partner violence (IPV). During the case the partner tries to speak for the patient. Pronouns for patient are he/him, she/hers, they/them, or any combination thereof* |
| **Primary Learning Objectives** | *-Identify red flags for when to suspect ongoing safety concerns*  *-Apply general principles of Trauma Informed Care*  *-Perform trauma screening for patients with suspected ongoing safety concerns*  *-Perform trauma-informed physical exam*  *-Apply techniques of separating suspected partners from patient/de-escalation techniques to prevent further altercation*  *-Apply techniques of counseling IPV patients* |
| **Critical Actions** | *-Introduce themselves and their role, pronouns*  *-Communicate with patients at same physical* level  **(*Have to make sure there are chairs in room)**  *-Ask permission for pronouns and preferred name*  *-Student does not come between door & patient*  *-Student offers patient the choice to opt out of questions*  *-Student provides anticipatory guidance by introducing questions*  *-Student asks about and does not assume relationship between two individuals in the room*  *-Student attempts to address the patient alone*  *-Student asks screening questions for IPV and patient safety*  *-Student obtains verbal consent before physical touch*  *-Students as a team will come up with short oral presentation of case, and will suggest disposition plan including whether to admit/discharge, who to consult, pain management plan, further imaging* |
| **Learner Preparation Prework** | *Didactic prior to session: will explain prevalence of IPV, specific questions about IPV that should be asked if suspected*  *-Specifically helpful questions in the case of IPV will be provided (as seen in checklist and didactic)* |

| **INITIAL PRESENTATION** | | | |
| --- | --- | --- | --- |
| **Initial vital signs** | **HR 110, BP 150/90, RR 20, SpO2 100%** | | |
| **Overall Setting and Appearance** | *-Patient is sitting up in bed, breathing heavily, appears to be in significant pain and discomfort while clutching arm*  **Extra: can make patient be intoxicated with alcohol for added clinical complexity**  *-Partner is sitting in a chair in the room by the patient* | | |
| **Standardized Participants (and their roles in the room at case start**) | 1. Patient: either patient actor or mannequin 2. Partner   *-Patient: story is intentionally vague, hesitant to respond to questions without looking over at partner for permission*  *-Partner: responds to questions for patient, interjects if patient tries to answer questions about the nature of the injury or prior injuries; exhibits resistance to being separated from the patient when asked to by medical team.* | | |
| **HPI** | *At first, patient reports that they fell down the stairs causing pain, but the story is intentionally vague. Initially involves tripping down the stairs, then changes to falling off a ladder while trying to change a light bulb. Initial report to students mentions a history of multiple prior falls and fractures in the ED, also which are intentionally vague. Once partner is separated from the patient, they open up about a longstanding history of IPV and trauma. They report several other ED visits in past for facial bone and rib fractures.*  **One-Liner:** Skyler Smith is a 26yo M presenting with acute-onset L arm pain, he has a history of prior fractures. | | |
| **Past Medical/Surgical History** | **Medications/Allergies** | **Social History** | **Family History** |
| - Hx of prior fractures - C-section - Asthma | - Albuterol - NKDA | - Unemployed - Lives with partner | Non-contributory |
| **Physical Examination** | | | |
| **General** | In distress and in pain, withdrawn | | |
| **HEENT** | Facial bruises, swelling around the R eye | | |
| **Neck** | Normal | | |
| **Lungs** | Normal | | |
| **Cardiovascular** | Tachycardic, otherwise nml. Cap refills <2 sec, radial and ulnar 2+ b/l | | |
| **Abdomen** | Normal | | |
| **MSK** | Swelling and ecchymosis noted to the wrist with exquisite tenderness to palpation, neurovascularly intact | | |
| **Neurological** | Normal. Dermatomal sensations intact along upper extremities b/l and normal motor function | | |
| **Skin** | Bruises and small abrasions in multiple stages of healing on multiple extremities | | |
| **GU** | N/A, normal | | |
| **Psychiatric** | Anxious, withdrawn (potentially intoxicated) | | |

| **INSTRUCTOR NOTES - CHANGES AND CASE BRANCH POINTS**  *This section should be a list with detailed description of each step than may happen during the case. If medications are given, what is the response? Do changes occur at certain time points? Should the nurse or other participant prompt the learners at given points? Should new actors or participants enter, and when? Are there specific things the patient will say or do at given times?* | | |
| --- | --- | --- |
| **Intervention / Time point** | **Change in Case** | **Additional Information** |
| *During interview* | Partner periodically tries to answer for patient and interject while speaks |  |
|  | Patient periodically looks at partner before answering questions as if for approval |  |
| *If the partner is asked to leave room* | Patient’s partner explicitly vocalizes disagreement (“I don’t feel comfortable leaving my partner”) and is insistent, will not allow students to speak with patient alone voluntarily |  |
| *While students are trying to convince partner to leave room* | Staff member (RN/Tech/MD) enters and asks: “Any labs or imaging you want at this point?”) | Students may either:  - Suggest X-ray imaging and explain to partner that per hospital policy only patients can go to radiology department  - Or continue to be persistent to find ways to speak to patient alone (deferring to need for hospital policy to ask confidential questions or to additional authority members)  -Partner finally leaves room, does not return to case |
| *Beginning of physical exam* | -Students now have opportunity to speak 1:1 with patient, ask for consent to examine patient  -Patient flinches visibly when students attempt to examine certain body parts (arm, stomach) | - CBC: no anemia - Labs: in normal range - X-ray: Minimally displaced fracture to L ulnar - Any other imaging ordered: normal |
| *Students counsel patient: suggest social work involvement, admission for safety, or other suggestions for safety* | Patient asks to maintain confidentiality but is interested in talking to social work. | - Social worker is consulted and will come see patient after |
| *End of case: students exit SIM room and enter main conference room to prepare presentation* | Case ends between patient team and patient: 5 minutes to prepare short oral presentation of most pertinent info and pain management, social work, and ortho plan. |  |
|  |  |  |

**Ideal Scenario Flow**

*The learners enter the room to find the patient and their partner in the room. They appropriately ask for pronouns and communicate at the same level as the patient (crouching or sitting in a chair). The relationship between the two individuals in the room is obtained and not assumed. Learners notice vague history with inconsistent details and the partner answering for the patient, thus preventing appropriate history/examination. An appropriate technique to speak to the patient alone is enacted, such as asking partner to step out of the room and deferring to hospital policy once met with resistance, or accompanying the patient to imaging themselves. A review of the patient’s chart reveals multiple visits for fractures and trauma. Patient is asked appropriate screening questions for intimate partner violence (Do you feel safe in your relationship? Does your partner ever abuse you physically? Emotionally? Sexually? Etc.) and for immediate patient safety (Are you in immediate danger? Do you have somewhere safe to go?). A sensitive physical exam is performed, and a plan is enacted to address the chief complaint, including an x-ray. Options for the patient to talk to a social worker, to receive resources for intimate partner violence, and/or involve legal officials are provided. The social worker is involved in the case and helps plan for safe disposition.*

**Anticipated Management Mistakes**

1. *Issue removing suspected perpetrator of abuse from the room: Some students were uncomfortable with the best strategies to talk to the patient alone when the partner resisted leaving the room. We found it helpful to highlight strategies in the pre-simulation didactic on how to address similar situations.*
2. *Failure to address key medical complaint: We found that students could become acutely focused on patient safety and intimate partner violence but may forget to exam/assess/treat the main medical complaint. We would prompt students if this was the case by asking if they wanted any additional tests or imaging for the chief complaint.*
3. *Uncertainty about resources and next steps: Many learners may be unsure about the best next steps after determining that there was intimate partner violence present. Specific debriefing information relevant to the hospital (social work, specific centers dedicated to the issue) and prompting for a social work consult were helpful to learners.*

| **Simulation 2**    **SIMULATION CASE TITLE: Gender-Affirming Surgery Post-Op Complications Case**      **LEARNER AUDIENCE: Medical Students** | |
| --- | --- |
| **PATIENT NAME: Riley Jones**    **PATIENT AGE: 29**    **CHIEF COMPLAINT: Post-operative bleeding & pain**    **PHYSICAL SETTING: Emergency Department** | |
|  | |
| **Brief narrative description of case** | *29 y/o trans F undergoing gender transition presents with vaginal bleeding and lower abdominal pain following a vaginoplasty for gender-affirming care performed 3 days ago. She suddenly began bleeding briskly and feels lightheaded from the amount of blood lost. Had one episode of syncope prior to arrival.* |
| **Primary Learning Objectives** | *-Students should recognize that though the patient is ill-appearing, the examination is potentially extremely traumatic and should take steps to minimize people in the room.*  *-Apply TIC principles by:*  **Obtaining consent prior to physical exam (pelvic exam)*  **Explaining exam maneuvers*  **Ask patient about pronouns, preferred anatomical parts terminology*  **Demonstrate appropriate response to patient actively dissociating during physical exam*  **Apply techniques of upstanding to faculty/staff that continually misgender the patient* |
| **Critical Actions** | *-Student introduces themselves and their role*  ***-****Student asks permission for pronouns and preferred name*  *-Student communicates with patient at same physical level, does not come between door and patient*  **(*Have to make sure there are chairs for all in room)**  *-Student offers patient the choice to opt out of questions*  *-Student provides anticipatory guidance*  *-Student obtains verbal consent before physical touch*  *-Student provides anticipatory guidance, introduces exam steps, and explains reasoning for exam maneuvers*  *-Student uses patient centered terms when discussing anatomy*  *-Student remains within eyesight of the patient throughout the encounter*  *-Student recognizes patient is distressed by exam and appropriately responds* |

| **INITIAL PRESENTATION** | | | |
| --- | --- | --- | --- |
| **Initial vital signs** | **BP 90/47, HR 120, RR 22, SpO2 100%, normal temp** | | |
| **Overall Setting and Appearance** | *Mannequin will have patient robe/sheet on, there will be some visible “blood,” patient will be audibly fatigued when speaking and complaining of pain in the lower abdominal/genital region. Cardiac monitor volume will remain on throughout case.* | | |
| **Standardized Participants (and their roles in the room at case start**) | 1. Standardized Patient (voicing mannequin) 2. Additional staff member | | |
| **HPI** | *The patient is presenting after feeling lightheaded. When asked about recent medical or surgical history, the patient reveals that she recently underwent vaginoplasty which seemed to go without issues and the patient was discharged 2 days later without complications, but started noticing some bleeding at home and pain, which became worse and is now causing the patient to feel lightheaded from lost blood.*  *If students ask further, the patient will reveal the patient has a traumatic history with prior medical providers misgendering her, as well as prior traumatic experiences with physical exams, particularly of the genital region in preparation for gender affirming surgery.*  **One Liner: Riley Jones is a 29 yo F who is presenting with fainting after “feeling lightheaded.”** | | |
| **Past Medical/Surgical History**  S/p vaginoplasty 3 days ago  No PMHx | **Medications/Allergies**   - Estrogens - NKDA | **Social History**   - Lives alone | **Family History**  Non-contributory |
| **Physical Examination** | | | |
| **General** | In distress and in pain, bloody clothes noted, cold to touch and clammy | | |
| **HEENT** | Normal | | |
| **Neck** | Normal | | |
| **Lungs** | Tachypneic, lungs are clear, using accessory muscles | | |
| **Cardiovascular** | Tachycardia otherwise nml | | |
| **Abdomen** | Tenderness to palpation along the suprapubic region/lower abdomen | | |
| **Neurological** | Mildly lethargic, no focal deficits, A&O x 3 and conversing normally otherwise | | |
| **Skin** | Pale, otherwise normal | | |
| **GU** | Brisk bleeding at site of vaginoplasty (this is reported over microphone) | | |
| **Psychiatric** | Anxious | | |

| **INSTRUCTOR NOTES - CHANGES AND CASE BRANCH POINTS**  *This section should be a list with detailed description of each step than may happen during the case. If medications are given, what is the response? Do changes occur at certain time points? Should the nurse or other participant prompt the learners at given points? Should new actors or participants enter, and when? Are there specific things the patient will say or do at given times?* | | |
| --- | --- | --- |
| **Intervention / Time point** | **Change in Case** | **Additional Information** |
| *~2-3 minutes into the case after initial history but before exam* | BP begins to decrease, HR increases | -Sim tech alerts team of lowering BP  - Patient appears uncomfortable, states “why are there so many people in the room? It feels so uncomfortable” |
|  | BP continues to decrease | Students at this point should order IV fluids and/or blood products  -If not being ordered spontaneously, RN or tech should come in and ask students what they want to do about unstable vitals |
| *Upon starting IV fluids/blood:* | BP and HR stabilize slowly: Patient begins complaining of “pain down there” (in post-surgical region) |  |
| *In preparation for physical exam* | Person acting as mannequin should prompt students a few minutes into the case to “take a look at what’s bleeding,” to prompt students to move on to physical exam portion if they haven’t yet done so. |  |
|  | Patient hesitates for significant amount of time to give verbal consent to exam | I.e. “um, I’m not sure, I don’t know how I feel about this.”  Students should be understanding and try to work with the patient to make her as comfortable as possible |
| *During physical exam* | Patient becomes noticeably quiet, a signal of active dissociation. Not responding to questions. | Students should respond by taking a break, checking in with patient to see how she is doing, an external exam is sufficient to reveal hemorrhage from surgical site |
| *After physical exam* | Staff member enters into room, asks for update, misgenders the patient in front of her (preferably more than once), then leaves room | Observe for student response to misgendering occurrence; if not spontaneous response the person acting as mannequin should prompt students by mentioning they are upset by this situation |
| *After physical exam is done* | Voice-over suggests: “maybe we should contact the on-call GYN or Plastics surgery team,” and keep pushing this as the right option | Voice-over not needed if students spontaneously suggest surgical consult |
| *At very end of case* | Students present case to attending/senior, have opportunity here to address misgendering incident, disposition (who to consult, blood products/maintenance fluids) | After this, case ends fully, begin debrief |

**Ideal Scenario Flow**

*The learners enter the room to find the patient, portrayed as a mannequin, in the room. Individuals appropriately ask for pronouns and communicate at the same level as the patient (crouching or sitting in a chair). The learners elicit history of a vaginoplasty by the patient and post-operative bleeding. When the patient becomes hemodynamically unstable, lab tests (including CBC) and treatment (IV fluids, prepare for blood products) are ordered. Once the vitals stabilize, the need for a physical exam of a sensitive area is recognized and the patient is made to feel comfortable during the process (for example: explanation of need for exam, offer to have other individuals step out of the room, anticipatory guidance on exam maneuvers, opportunities for patient to request to stop the exam, use of patient-centered terminology for anatomy). When another staff member misgenders the patient, appropriate steps are taken to address the situation and reassure patient.*

**Anticipated Management Mistakes**

1. *Discomfort in obtaining history and exam: Some students were unsure how to sensitively elicit details about the surgery that may be relevant for patient care (specifics of surgical procedure, relevant questions for potential complications) and how to examine a sensitive area. We found it helpful to prompt learners to perform the exam given the need to understand where the source of hemorrhage was located.*
2. *Delayed treatment for hemodynamic instability: We found that students sometimes did not respond quickly to the instable vitals. In this case, we prompted via voice over or with someone playing the resident/attending in-person to see if the students wanted labs or treatment at the time.*
3. *Failure to address misgendering incident with both patient & staff member: Several learners did not address the issue of misgendering with the staff member either before or during the incident. During the debriefing, we found it helpful to discuss what could be improved with their response (clarifying pronouns in front of the staff member, apologizing & reassuring patient that appropriate steps would be enacted, and/or talking to the staff member outside of the room).*

| **Simulation 3**    **SIMULATION CASE TITLE: Drug Use & Medical Distrust Case**      **LEARNER AUDIENCE: Medical Students** | |
| --- | --- |
| **PATIENT NAME: Morgan Peters**    **PATIENT AGE: 32 yo**    **CHIEF COMPLAINT: R arm pain**    **PHYSICAL SETTING: Urgent Care** | |
|  | |
| **Brief narrative description of case** | *32 y/o X previously unknown patient presents for care to a primary care office requesting treatment of R arm pain, redness, and swelling going on for the last 4 days and progressively worsening, now with severe pain and feeling general malaise. The patient at first reports that the wounds on his arm are due to a cat bite*  *- Patient goes through withdrawal symptoms, to help tip off students that the patient has been using IV drugs* |
| **Primary Learning Objectives** | *-Recognize manifestations of medical trauma*  *-Navigate sensitive HPI including drug history, housing, safety* |
| **Critical Actions (bolded are most important)** | *-Student introduces themselves & role*  *-Student communicates with patient at same level*  *-Student does not come between door & patient*  *-Student offers patient choice to opt out of questions*  *-Student provides anticipatory guidance*  *-Student addresses the staff member who uses stigmatizing language about the patient by checking in with the patient*  *-Student uses person-centered language, avoiding stigmatizing words*  *-Student asks about social history, particularly substance use, housing, safety*  *-Student obtains verbal consent before physical touch*  *-Student provides anticipatory guidance regarding exam maneuvers*  *-Student remains within eyesight of the patient throughout the encounter* |
| **Learner Preparation or Prework** | *Didactic prior to session* |

| **INITIAL PRESENTATION** | | | |
| --- | --- | --- | --- |
| **Initial vital signs** | **HR 112, BP 105/65, RR 15, SpO2 100%, Temp 38.3 C (101 F)** | | |
| **Overall Setting and Appearance** | *What do learners see when they first enter the room? What environment are the learners in? What is the appearance of the mannequin?*  Standardized patient in room sitting on the chair, appears to be in discomfort and fatigued | | |
| **Standardized Participants (and their roles in the room at case start**) | *Who is present at the beginning and what is their role? Who may play them? Describe what they should say (i.e., their verbal scripts).*   1. Standardized Patient 2. Attending | | |
| **HPI** | *States that his/her cat bit him/her on the R arm and that is the origin of his/her wound. Later on, patient will reveal that he/she has been mistreated and called an addict in previous EDs, and that at one time a nurse told him/her “you won’t get any drugs in this ED,” which caused him/her to mistrust the healthcare system. He/she states, “I get a lot of those nasty pimples when I use, but when I come to the ER I just lie about using IV drugs ever since that time.” Further social history will reveal that the patient is unhoused, last used IV fentanyl about 2 days prior, and is currently experiencing withdrawal symptoms as well as febrile symptoms from developing cellulitis/abscess.*  **One Liner:** Morgan Peters is a 32 y/o X patient, well known to the ED, who presents after progressively worsening R arm pain and malaise. | | |
| **Past Medical/Surgical History** | **Medications/Allergies** | **Social History** | **Family History** |
| -HTN (untreated) | -No current home meds, but medication review from chart history reveals:  Naloxone & Methadone scripts in the past  - NKDA | - Unhoused - Drug Use: Heroin, Fentanyl, occasionally mixed with cocaine | Non-contributory |
| **Physical Examination** | | | |
| **General** | Disheveled, appears to be in discomfort, fatigued, actively vomiting periodically during encounter, warm to touch/febrile | | |
| **HEENT** | Normal | | |
| **Neck** | Normal | | |
| **Lungs** | Normal | | |
| **Cardiovascular** | Tachycardic, no murmurs/rubs/gallops | | |
| **Abdomen** | Diffuse generalized discomfort, no rebound or guarding, no other abnormalities | | |
| **Neurological** | Normal | | |
| **Skin** | Streak marks noted in bilateral antecubital fossa, cellulitis noted in L antecubital fossa area with induration and central fluctuance, exquisitely tender, prominent piloerection noted | | |
| **GU** | N/A | | |
| **Psychiatric** | Anxious, mildly withdrawn | | |

| **INSTRUCTOR NOTES - CHANGES AND CASE BRANCH POINTS**  *This section should be a list with detailed description of each step that may happen during the case. If medications are given, what is the response? Do changes occur at certain time points? Should the nurse or other participant prompt the learners at given points? Should new actors or participants enter, and when? Are there specific things the patient will say or do at given times?* | | |
| --- | --- | --- |
| **Intervention / Time point** | **Change in Case** | **Additional Information** |
| *During Interview* | Patient appears reluctant to disclose information | *Pt discloses that wound was caused by “cat bite” a few days ago. If asked about nausea/vomiting, mentions that this intermittently happens but is initially vague about the details.* |
| *Physical Exam* | Patient winces when arm is touched  During exam patient is exhibiting withdrawal symptoms, actively vomiting/retching, shivering, widened pupils | *Students must obtain consent before exam. Exam reveals track marks, clues students in to more than just a “cat bite”.* |
| *After Physical Exam/ ~ 10 mins into case* | Attending enters room, talks to students and tells them “He comes to the ED all the time and makes stuff up so he can stay longer.”  May also ask students (reluctantly/sarcastically): “anything you want to order for him? Labs/imaging/drugs” | Hopefully students will recognize serious cellulitis/forearm abscess and will order:   - CBC w diff - BMP - POCUS - Meds for withdrawal sx: Benzodiazepines - +/- empiric Abx & IVF |
| *If students have not asked about drug use specifically* | Make story intentionally vague, make statements like, “Well, they called me an addict, I guess that’s not that far from the truth….I do use IV drugs sometimes” | Patient reveals in the past has been actively discriminated against and treated poorly for revealing they use IV drugs and in withdrawal, that this is the reason they now lie whenever they have to come to ED, and that usually they avoid the ED at all costs because of this |
| *After patient discloses recent IVDU* | Patient discloses any drug-related questions students ask about (types of drugs, frequency, paraphernalia use) | Drugs: heroin, fentanyl, sometimes mixed with cocaine  Frequency: 1-2 times per day x several years  Paraphernalia: licking needles, sharing needles, no access to syringe exchange, uses IV drugs alone, no naloxone access, uses belt as tourniquet, uses tap water and lemon juice to dissolve/cook heroin  **all of these details are available, only to be revealed if students ask about these things specifically, otherwise not to be mentioned* |

**Ideal Scenario Flow**

*The learners enter the room to find the patient who is uncomfortable and in clear distress. A vague history that mentions that these symptoms have happened to him before and about the incident of the cat bite are obtained. If asked or after an amount of time has elapsed, the history of IV drug use is elicited. A detailed physical exam of the extremities and abdomen are performed. Learners appropriately respond to the stigmatizing language used by the attending by addressing the issue with the attending and the patient, while advocating for their medical needs. Stigmatizing language such as “addict” and “frequent-flyer” are not used. Questions about their social history are elicited, including about their socioeconomic situation (asking about housing, insurance, primary care, etc.) and drug use (last use, method of use, safety precautions, etc.).*

**Anticipated Management Mistakes**

1. *Failure to address use of stigmatizing language with both patient & attending: Several learners did not address the issue of stigmatizing language with the attending. During the debriefing, we found it helpful to discuss what could be improved with their response (apologizing & reassuring patient that appropriate steps would be enacted, talking to the attending outside of the room by framing it in terms of the patient discomfort, and/or advocating for the patient’s medical needs).*
2. *Omission of questions related to risk reduction: Many learners did not ask any questions related to risk reduction of IV drug use (last use, method of use, safety precautions, etc.). We incorporated specific questions to ask patients who use IV drugs and potential infectious disease concerns into our debriefing materials.*
