# Supplementary material for: Trauma-Informed Care for Acute Care Settings: A Novel Simulation Training for Medical Students
Source: MedEdPORTAL. 2023 Jul 28;19:11327. doi: 10.15766/mep_2374-8265.11327 (PMC10376910; doi:10.15766/mep_2374-8265.11327)
Supplement: Supplementary file 1 — TIC Acute Care Didactic.pptxSimulation Cases.docxDebriefing Materials.docxSimulation Checklists.docxSurvey Questions.docx [file mep_2374-8265.11327-s001.zip › A. TIC Acute Care Didactic.pptx]

## Slide 1
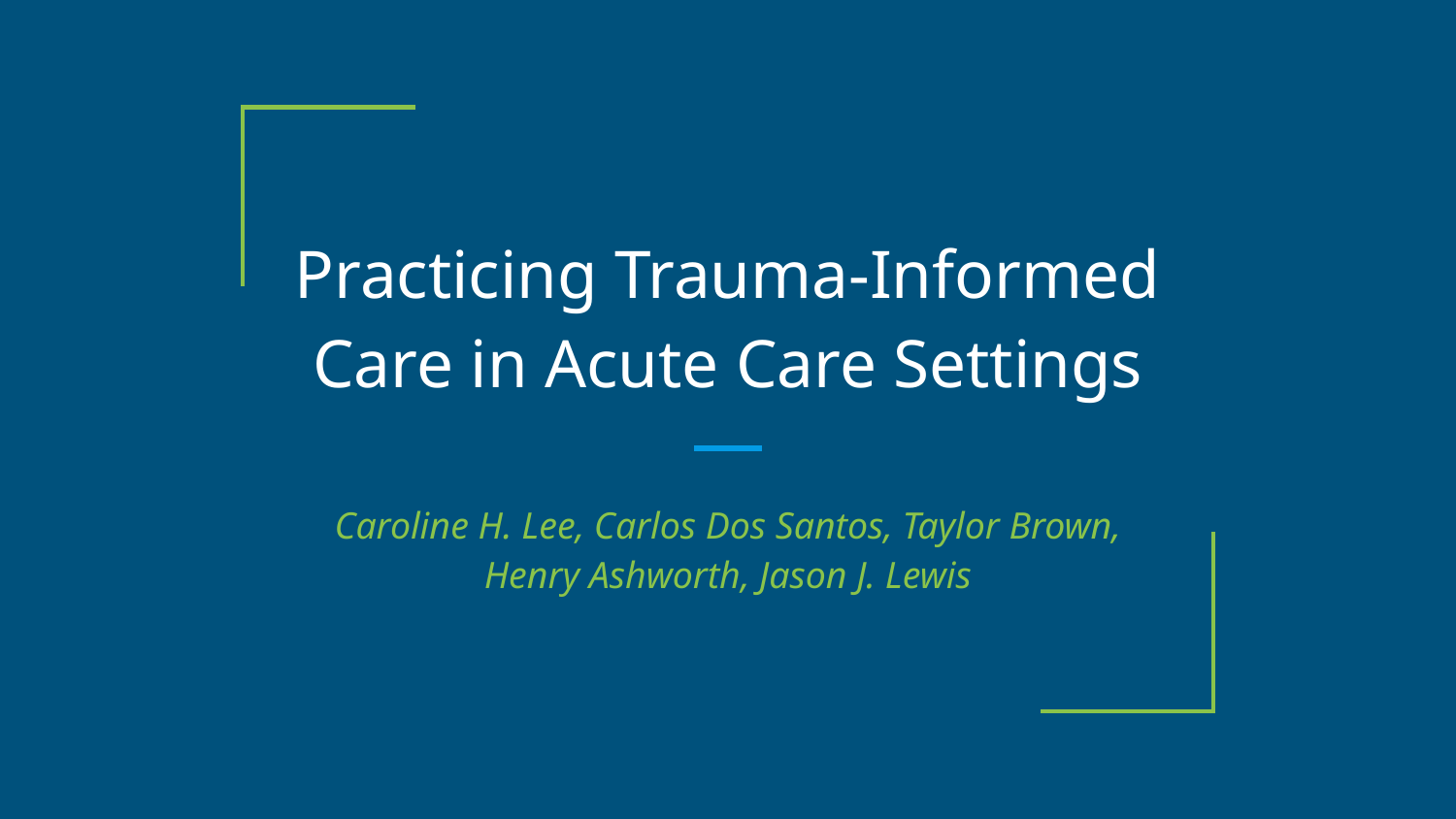

# Practicing Trauma-Informed Care in Acute Care Settings
Caroline H. Lee, Carlos Dos Santos, Taylor Brown, Henry Ashworth, Jason J. Lewis

## Slide 2
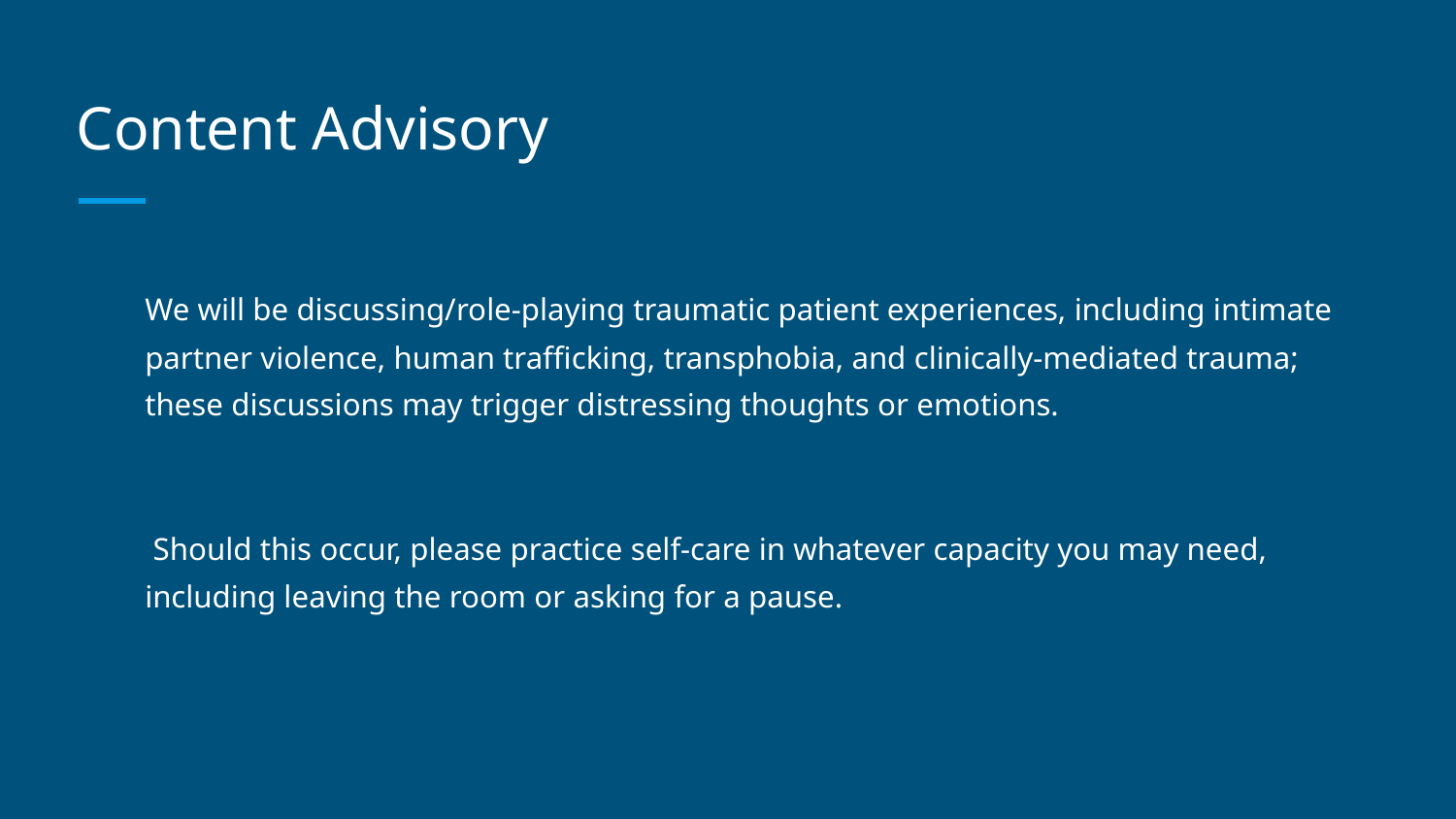

# Content Advisory
We will be discussing/role-playing traumatic patient experiences, including intimate partner violence, human trafficking, transphobia, and clinically-mediated trauma; these discussions may trigger distressing thoughts or emotions.
 Should this occur, please practice self-care in whatever capacity you may need, including leaving the room or asking for a pause.

## Slide 3
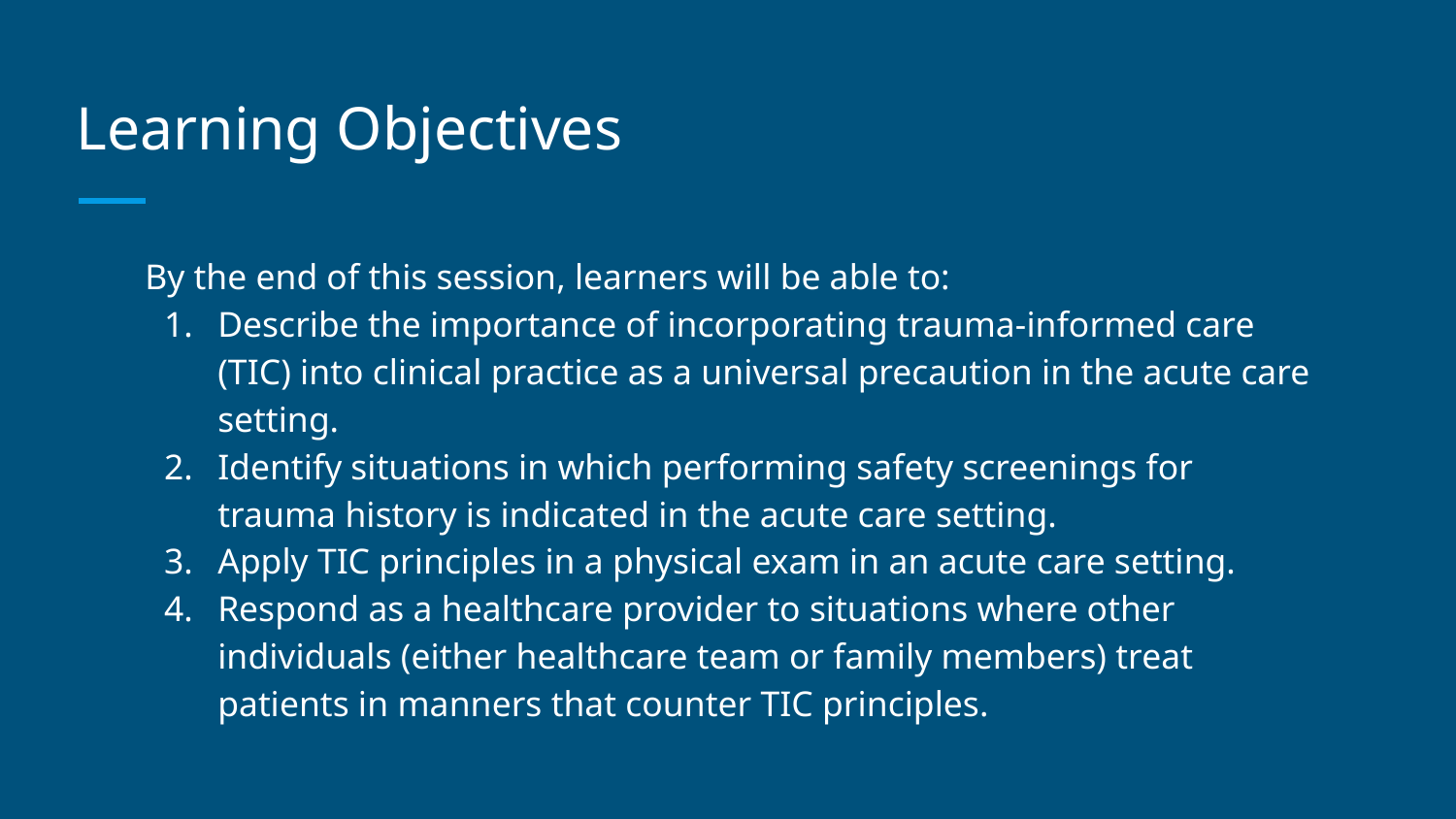

# Learning Objectives
By the end of this session, learners will be able to:
Describe the importance of incorporating trauma-informed care (TIC) into clinical practice as a universal precaution in the acute care setting.
Identify situations in which performing safety screenings for trauma history is indicated in the acute care setting.
Apply TIC principles in a physical exam in an acute care setting.
Respond as a healthcare provider to situations where other individuals (either healthcare team or family members) treat patients in manners that counter TIC principles.

## Slide 4
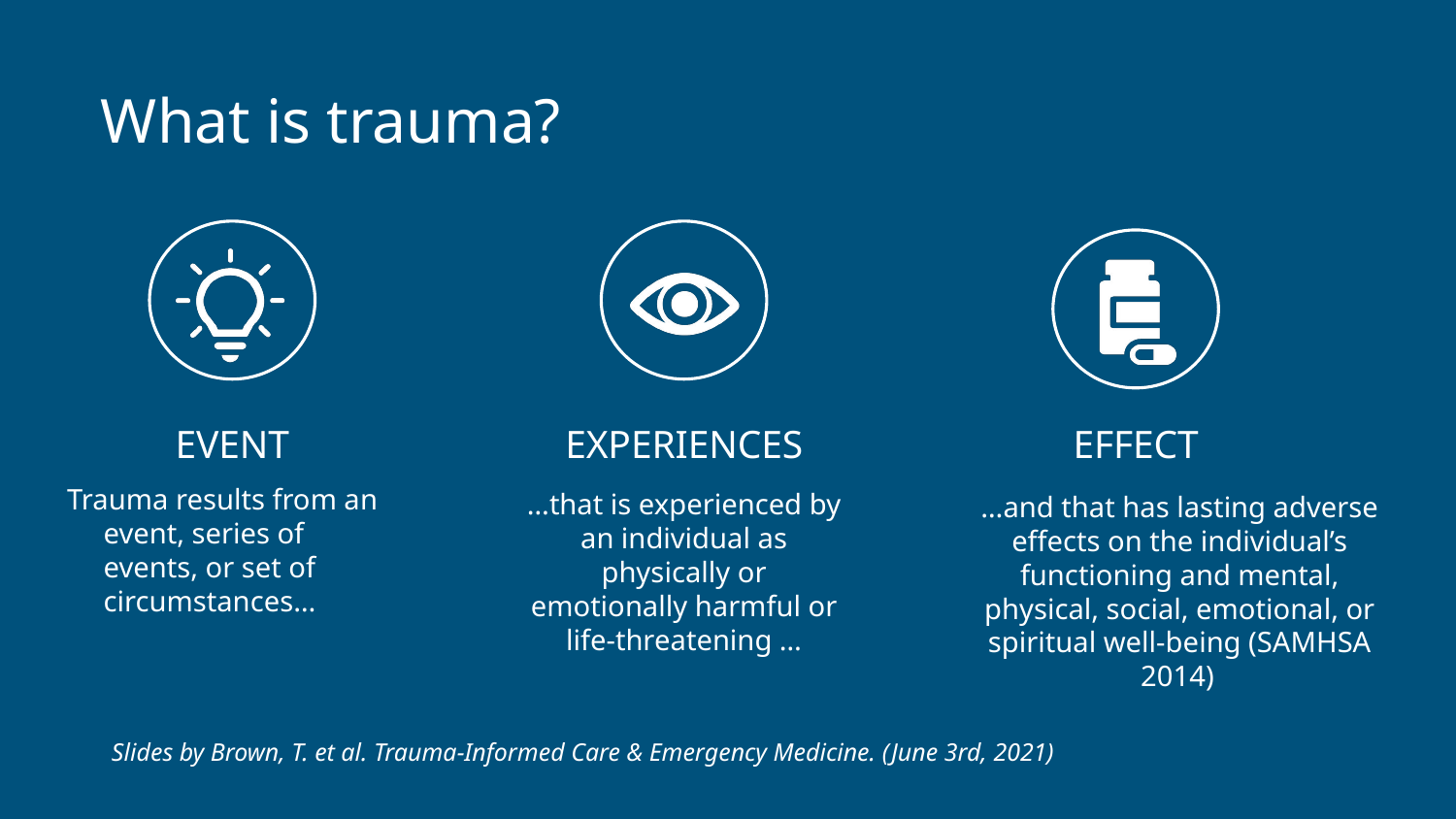

# What is trauma?
EVENT
EXPERIENCES
EFFECT
Trauma results from an event, series of events, or set of circumstances…
…that is experienced by an individual as physically or emotionally harmful or life-threatening …
…and that has lasting adverse effects on the individual’s functioning and mental, physical, social, emotional, or spiritual well-being (SAMHSA 2014)
Slides by Brown, T. et al. Trauma-Informed Care & Emergency Medicine. (June 3rd, 2021)

## Slide 5
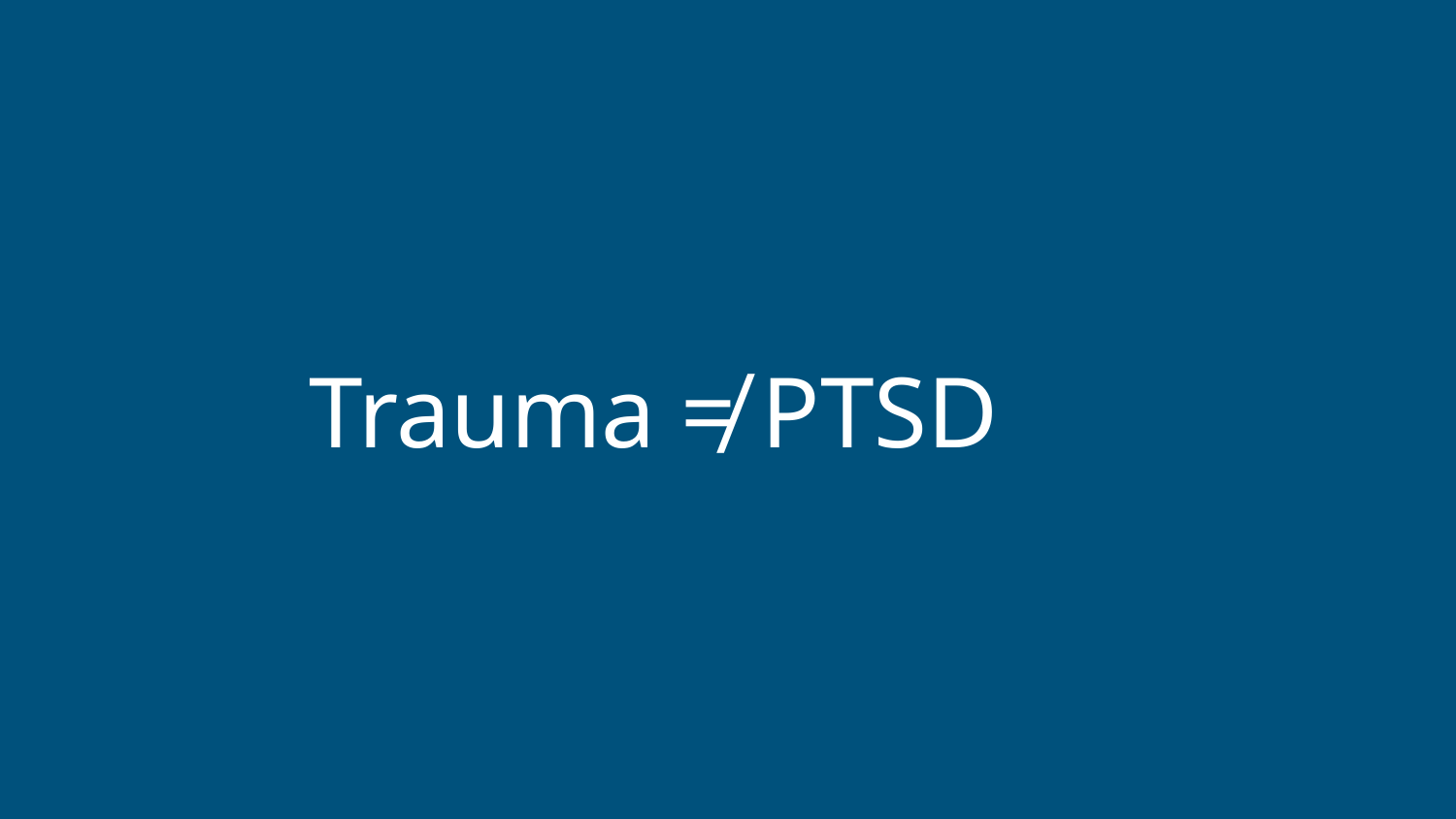

# Trauma ≠ PTSD

## Slide 6
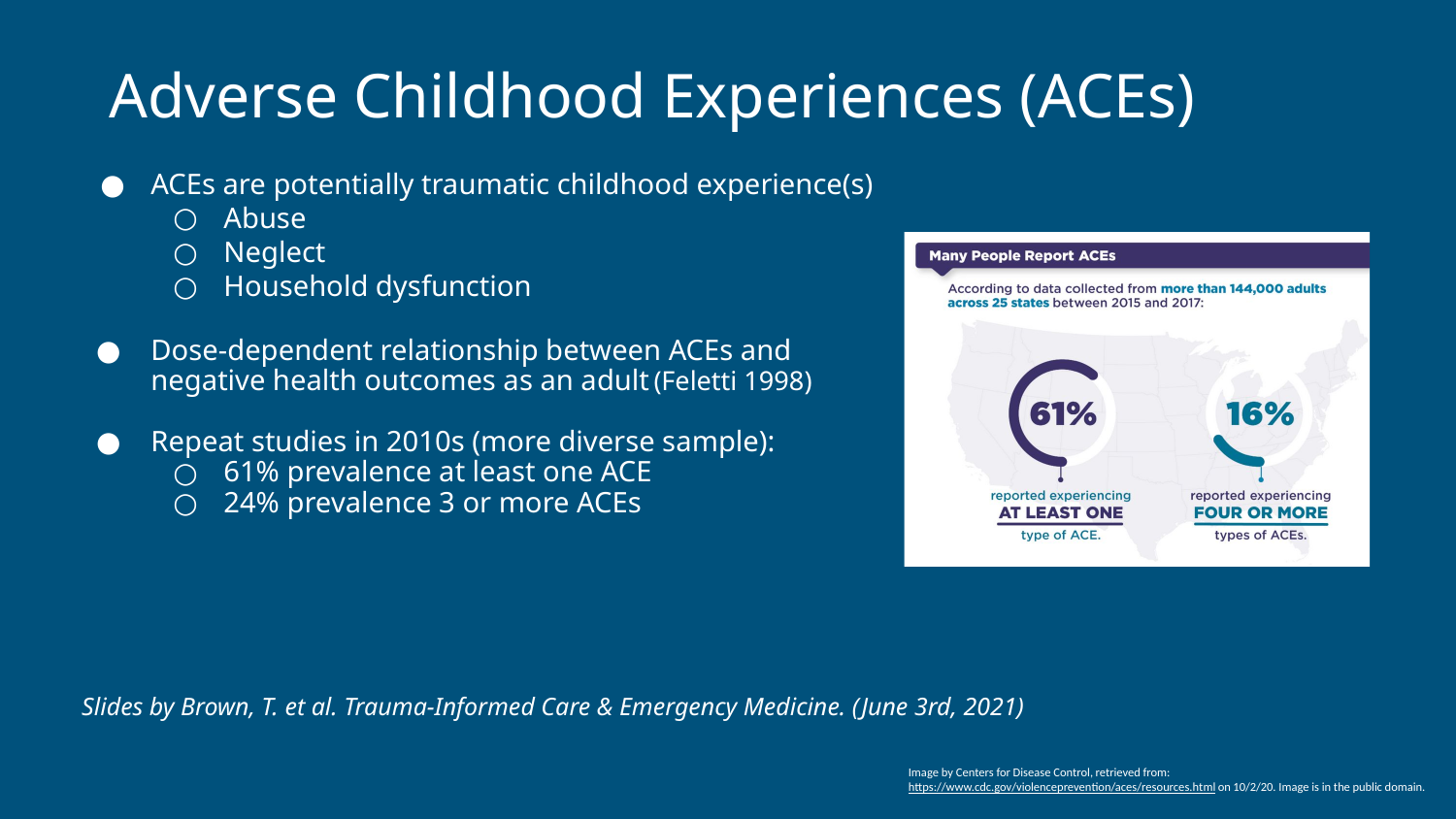

# Adverse Childhood Experiences (ACEs)
ACEs are potentially traumatic childhood experience(s)
Abuse
Neglect
Household dysfunction
Dose-dependent relationship between ACEs and negative health outcomes as an adult (Feletti 1998)
Repeat studies in 2010s (more diverse sample):
61% prevalence at least one ACE
24% prevalence 3 or more ACEs
Slides by Brown, T. et al. Trauma-Informed Care & Emergency Medicine. (June 3rd, 2021)
Image by Centers for Disease Control, retrieved from: https://www.cdc.gov/violenceprevention/aces/resources.html on 10/2/20. Image is in the public domain.

## Slide 7
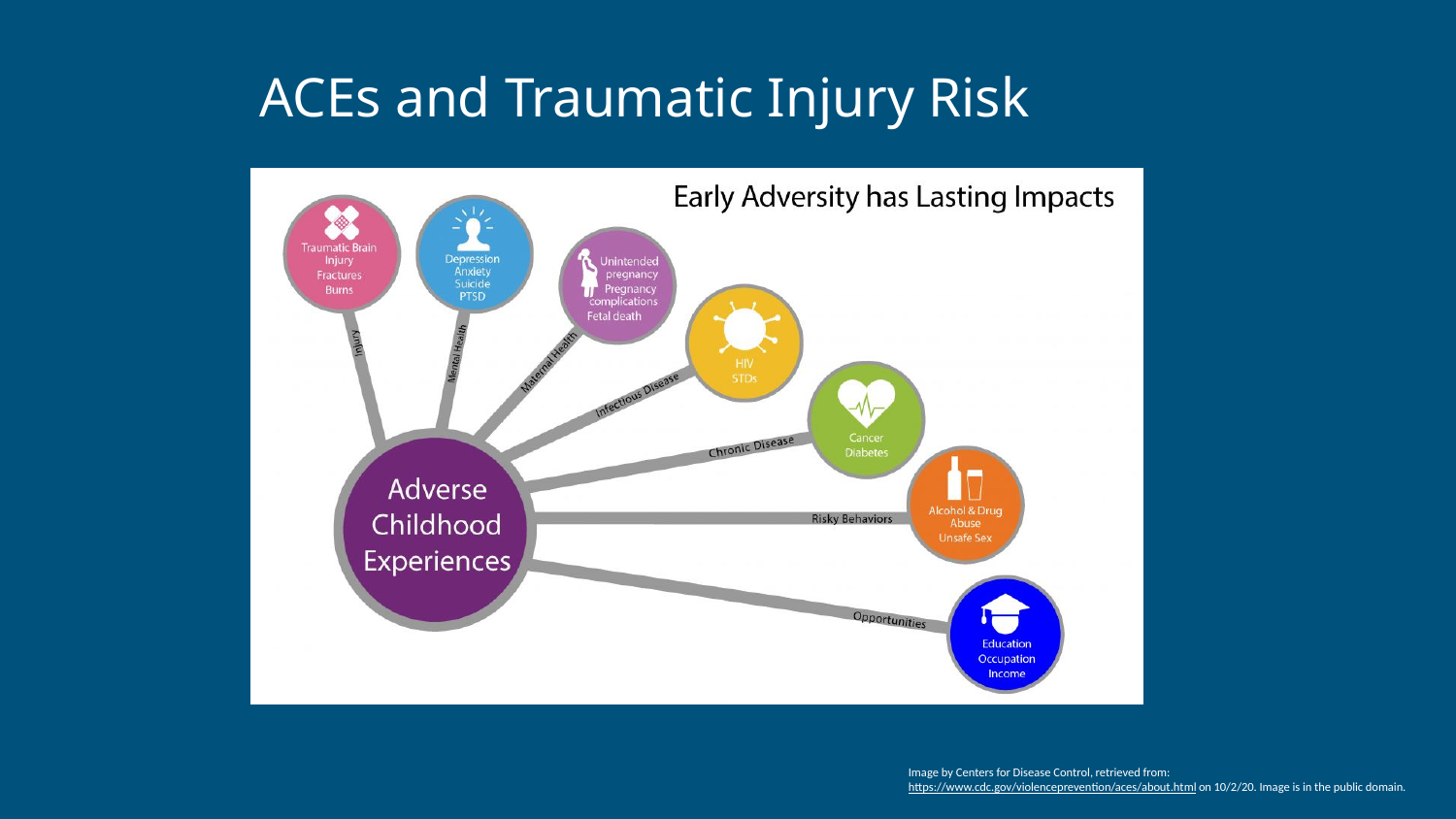

# ACEs and Traumatic Injury Risk
Image by Centers for Disease Control, retrieved from: https://www.cdc.gov/violenceprevention/aces/about.html on 10/2/20. Image is in the public domain.

## Slide 8
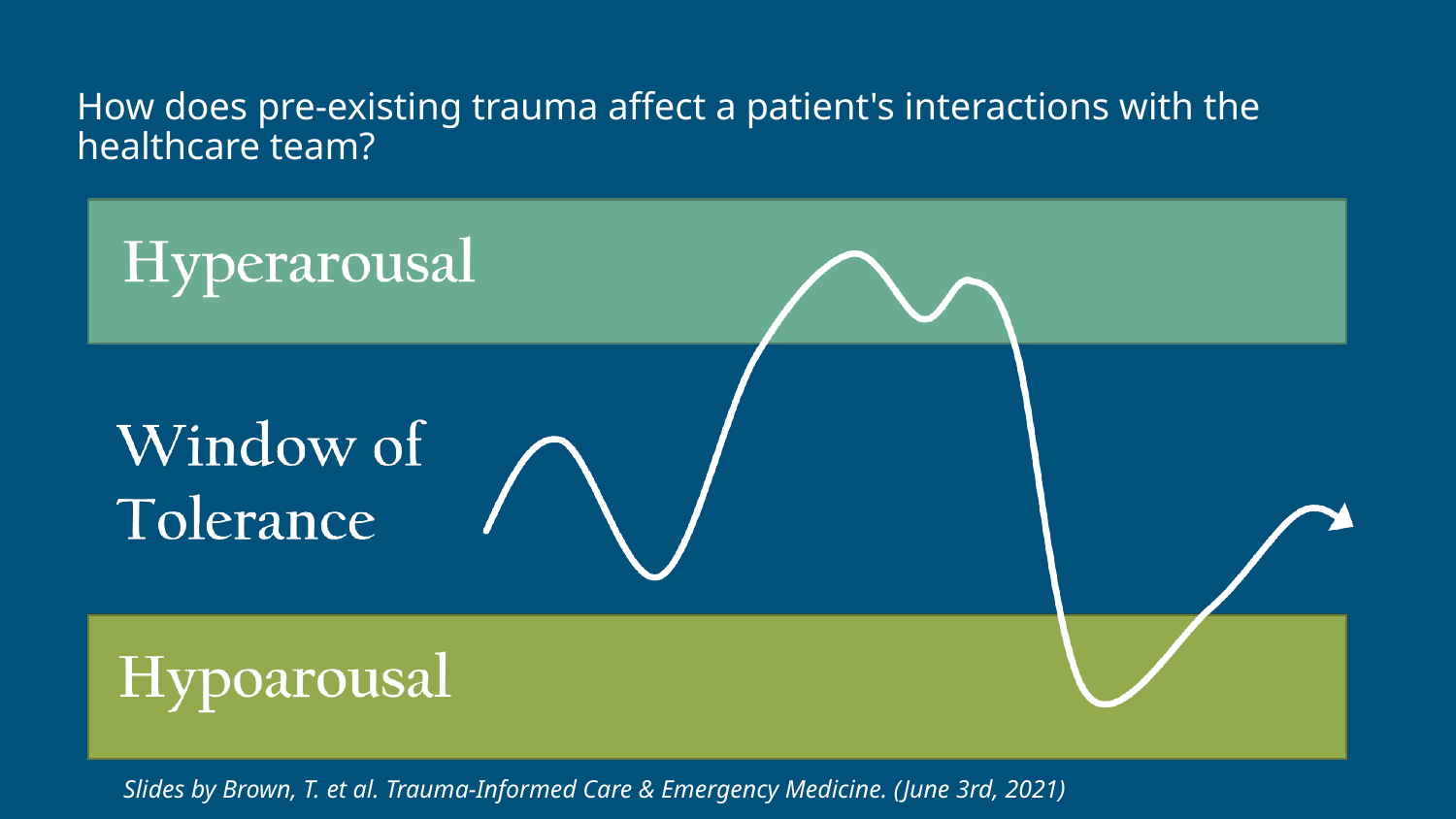

# How does pre-existing trauma affect a patient's interactions with the healthcare team?
Slides by Brown, T. et al. Trauma-Informed Care & Emergency Medicine. (June 3rd, 2021)

## Slide 9
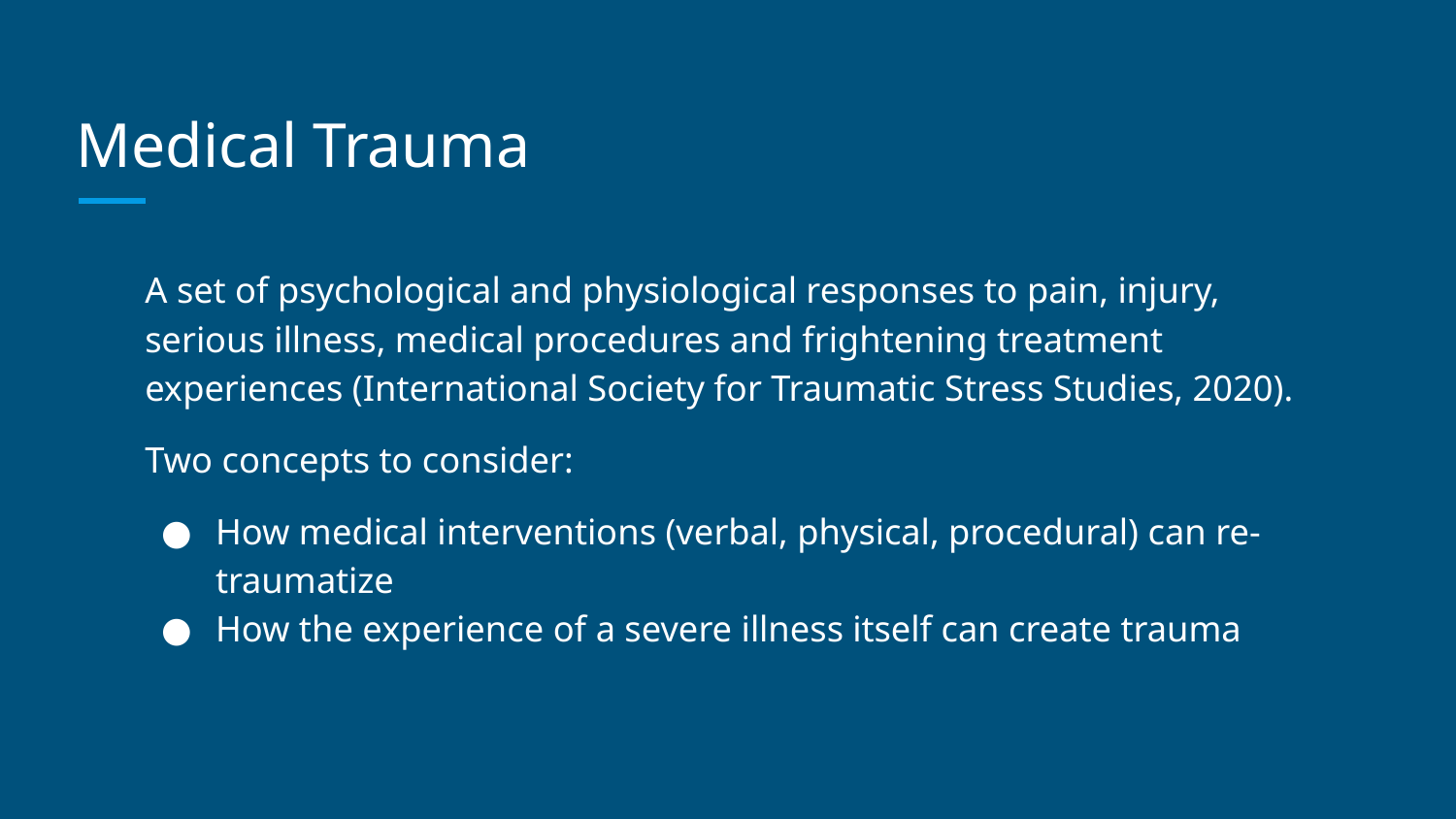

# Medical Trauma
A set of psychological and physiological responses to pain, injury, serious illness, medical procedures and frightening treatment experiences (International Society for Traumatic Stress Studies, 2020).
Two concepts to consider:
How medical interventions (verbal, physical, procedural) can re-traumatize
How the experience of a severe illness itself can create trauma

## Slide 10
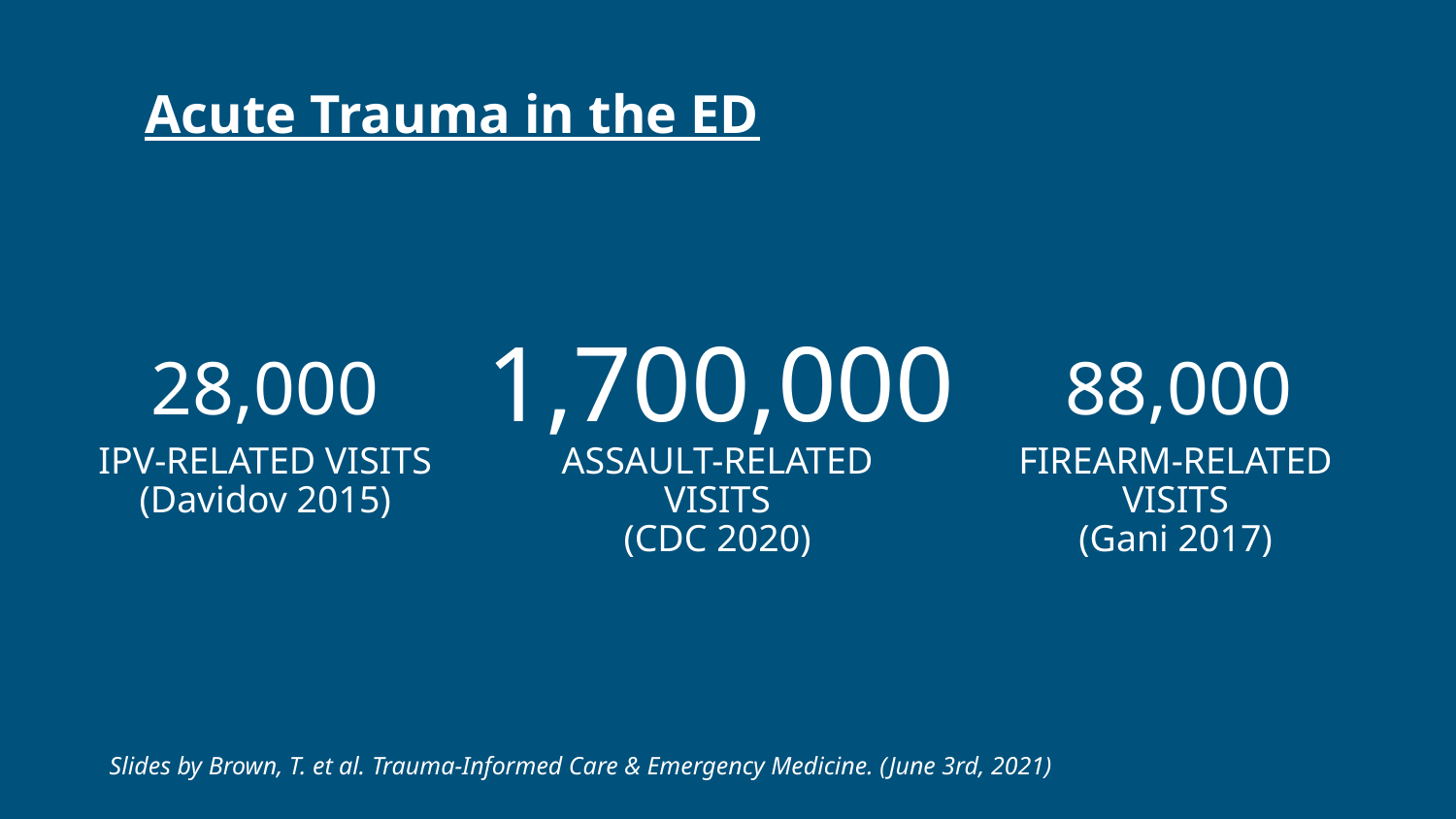

Acute Trauma in the ED
28,000
88,000
# 1,700,000
IPV-RELATED VISITS
(Davidov 2015)
ASSAULT-RELATED VISITS
(CDC 2020)
FIREARM-RELATED VISITS
(Gani 2017)
Slides by Brown, T. et al. Trauma-Informed Care & Emergency Medicine. (June 3rd, 2021)

## Slide 11
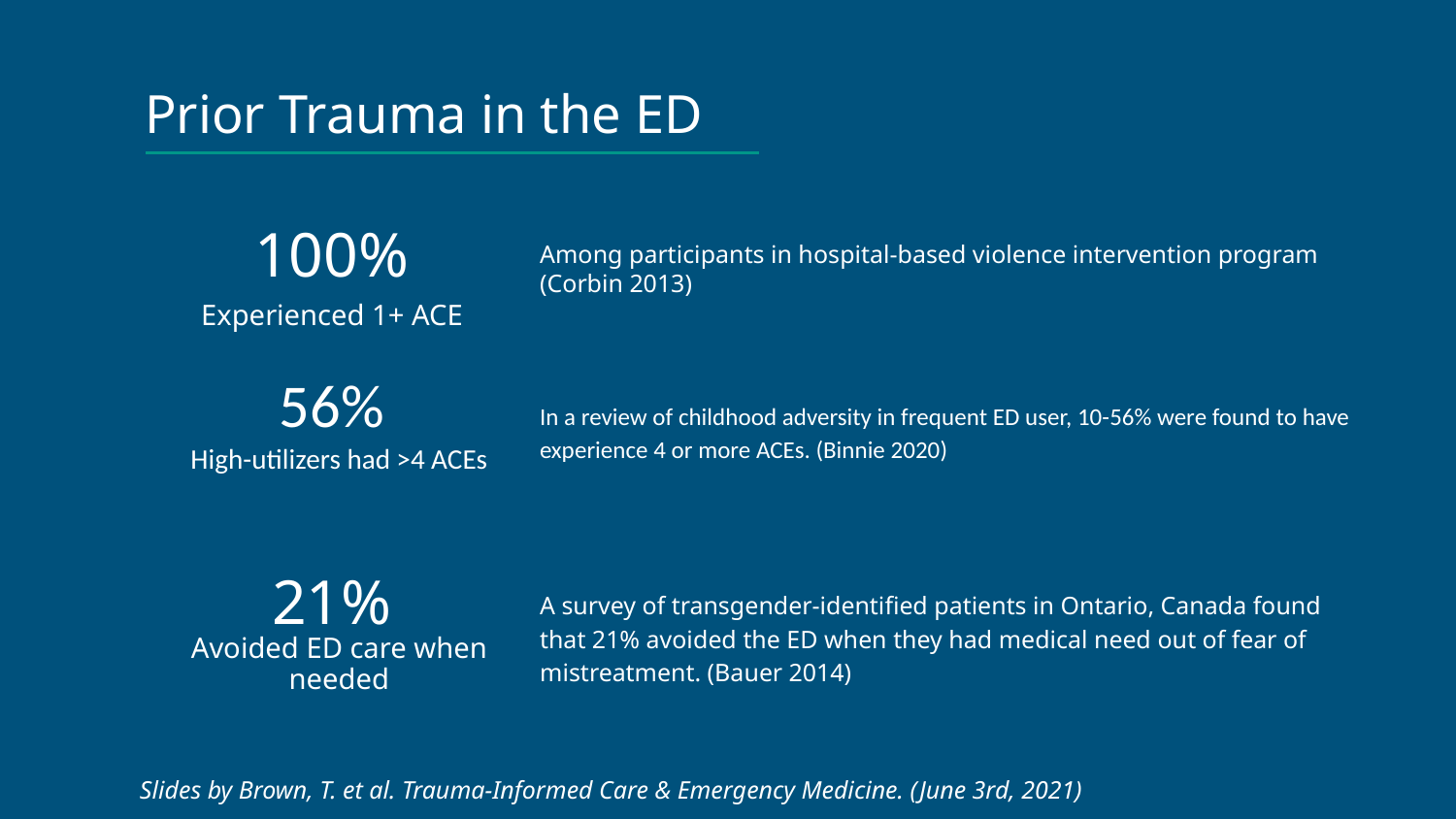

Prior Trauma in the ED
100%
Among participants in hospital-based violence intervention program (Corbin 2013)
Experienced 1+ ACE
56%
In a review of childhood adversity in frequent ED user, 10-56% were found to have experience 4 or more ACEs. (Binnie 2020)
High-utilizers had >4 ACEs
21%
A survey of transgender-identified patients in Ontario, Canada found that 21% avoided the ED when they had medical need out of fear of mistreatment. (Bauer 2014)
Avoided ED care when needed
Slides by Brown, T. et al. Trauma-Informed Care & Emergency Medicine. (June 3rd, 2021)

## Slide 12
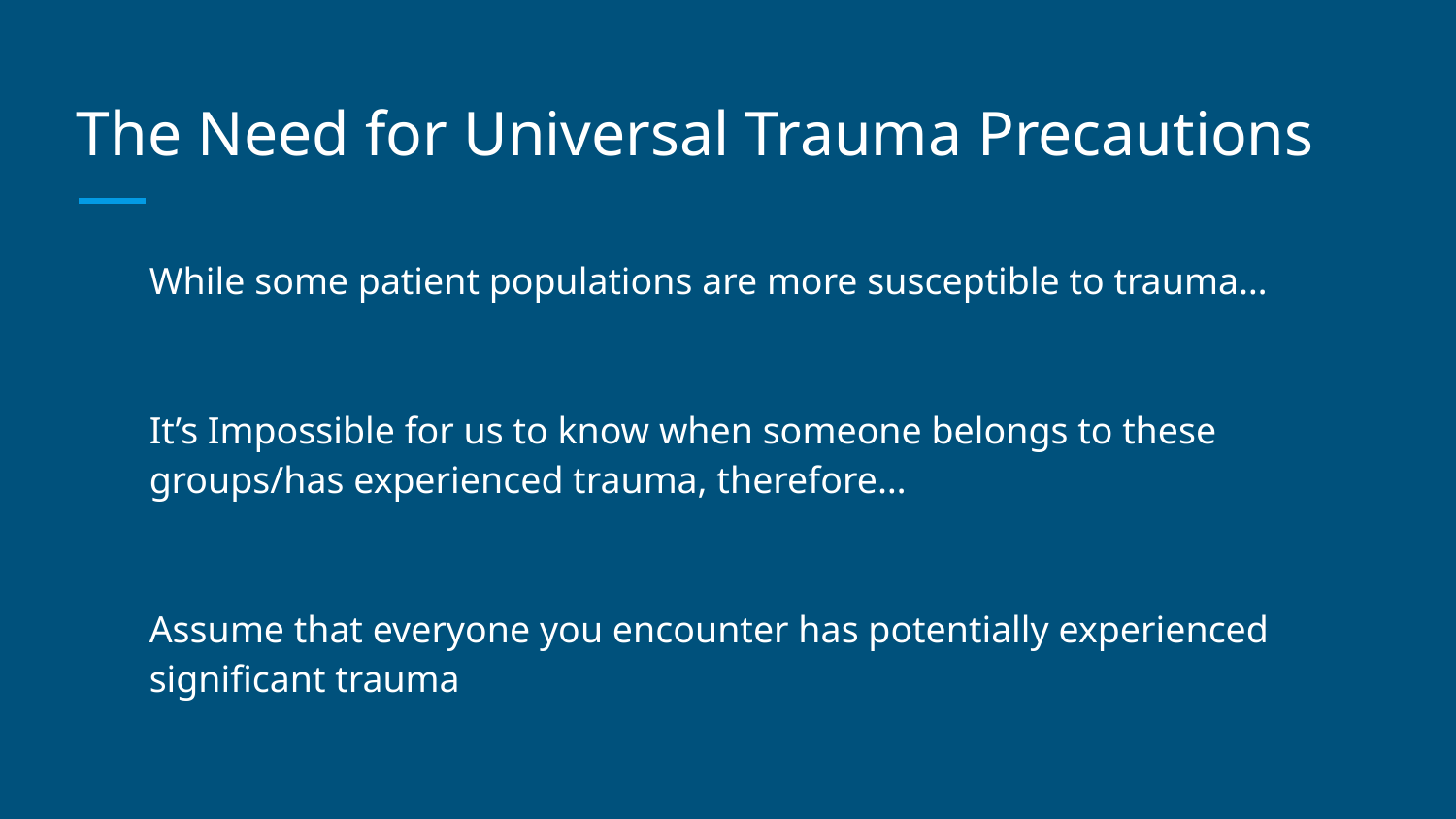

# The Need for Universal Trauma Precautions
While some patient populations are more susceptible to trauma…
It’s Impossible for us to know when someone belongs to these groups/has experienced trauma, therefore…
Assume that everyone you encounter has potentially experienced significant trauma

## Slide 13
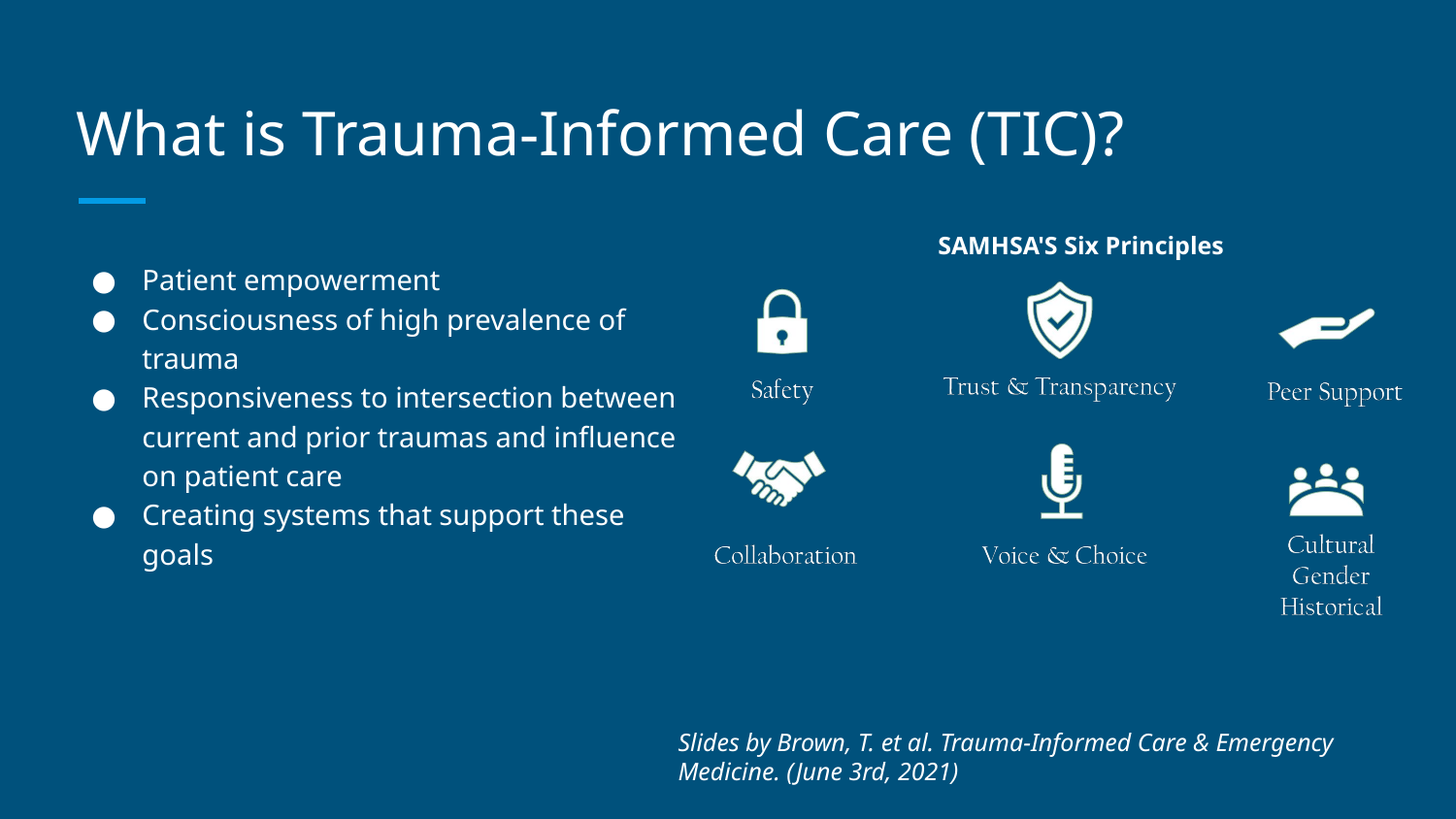

# What is Trauma-Informed Care (TIC)?
SAMHSA'S Six Principles
Patient empowerment
Consciousness of high prevalence of trauma
Responsiveness to intersection between current and prior traumas and influence on patient care
Creating systems that support these goals
Slides by Brown, T. et al. Trauma-Informed Care & Emergency Medicine. (June 3rd, 2021)

## Slide 14
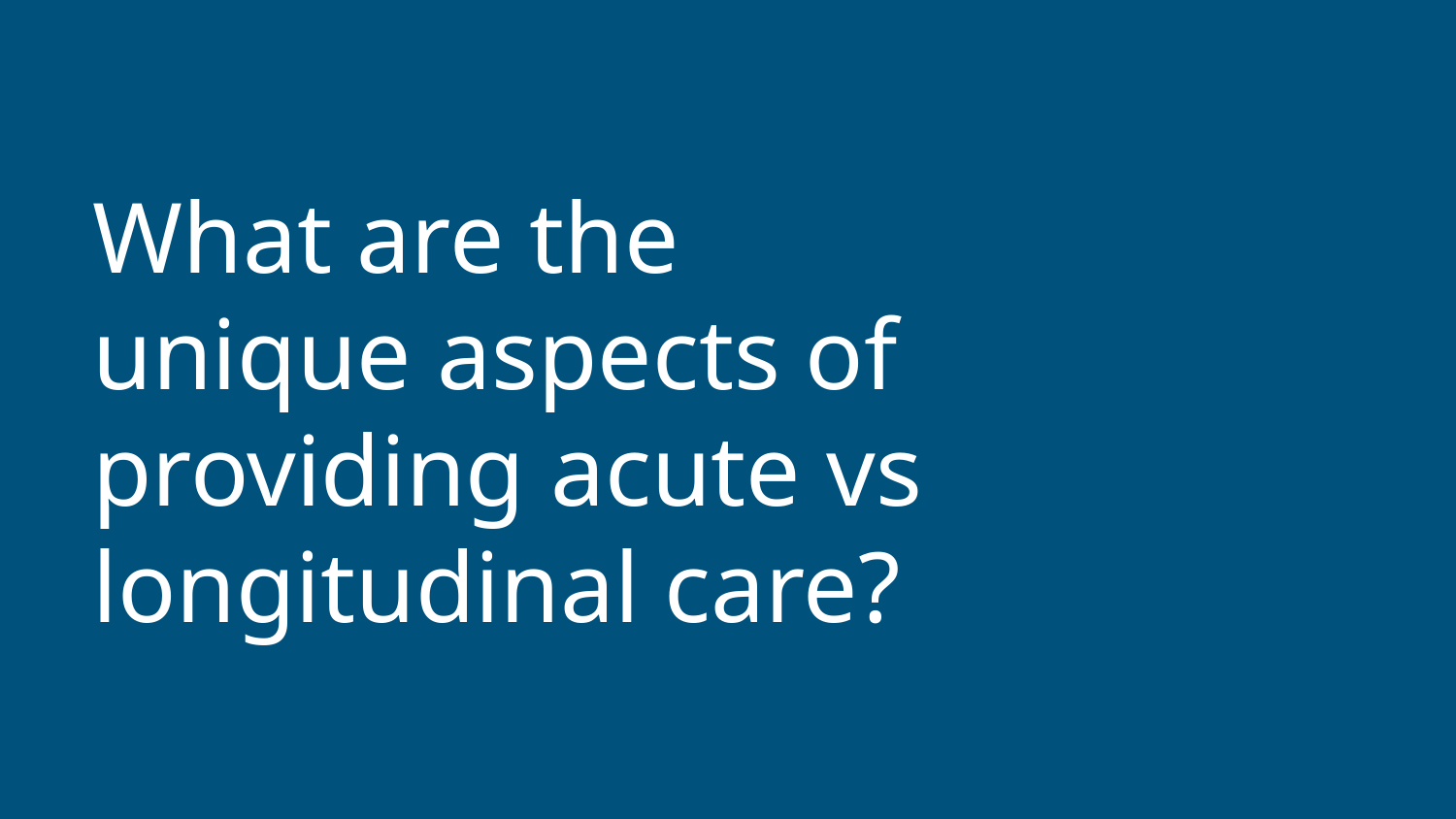

# What are the unique aspects of providing acute vs longitudinal care?

## Slide 15
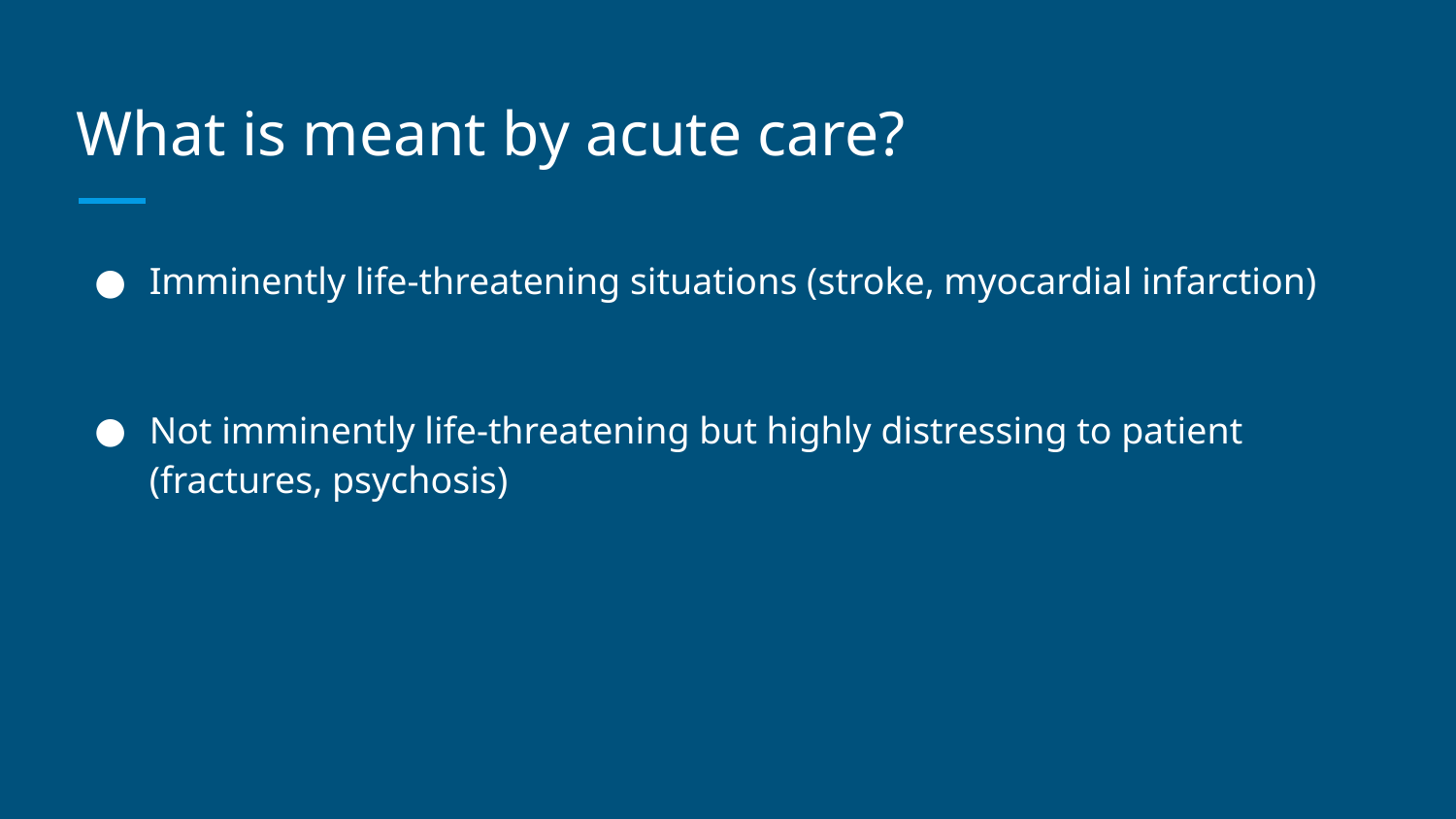

# What is meant by acute care?
Imminently life-threatening situations (stroke, myocardial infarction)
Not imminently life-threatening but highly distressing to patient (fractures, psychosis)

## Slide 16
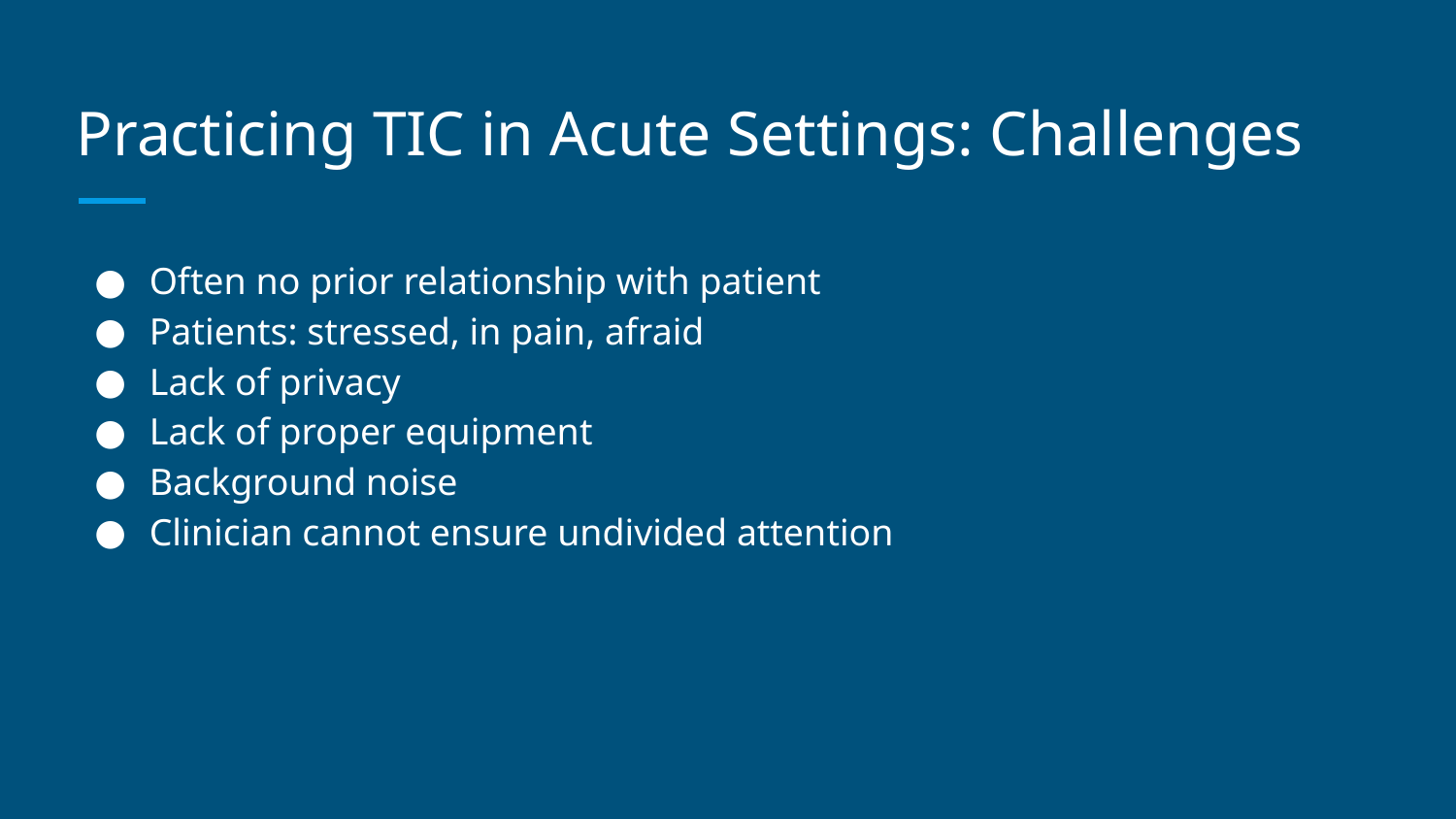

# Practicing TIC in Acute Settings: Challenges
Often no prior relationship with patient
Patients: stressed, in pain, afraid
Lack of privacy
Lack of proper equipment
Background noise
Clinician cannot ensure undivided attention

## Slide 17
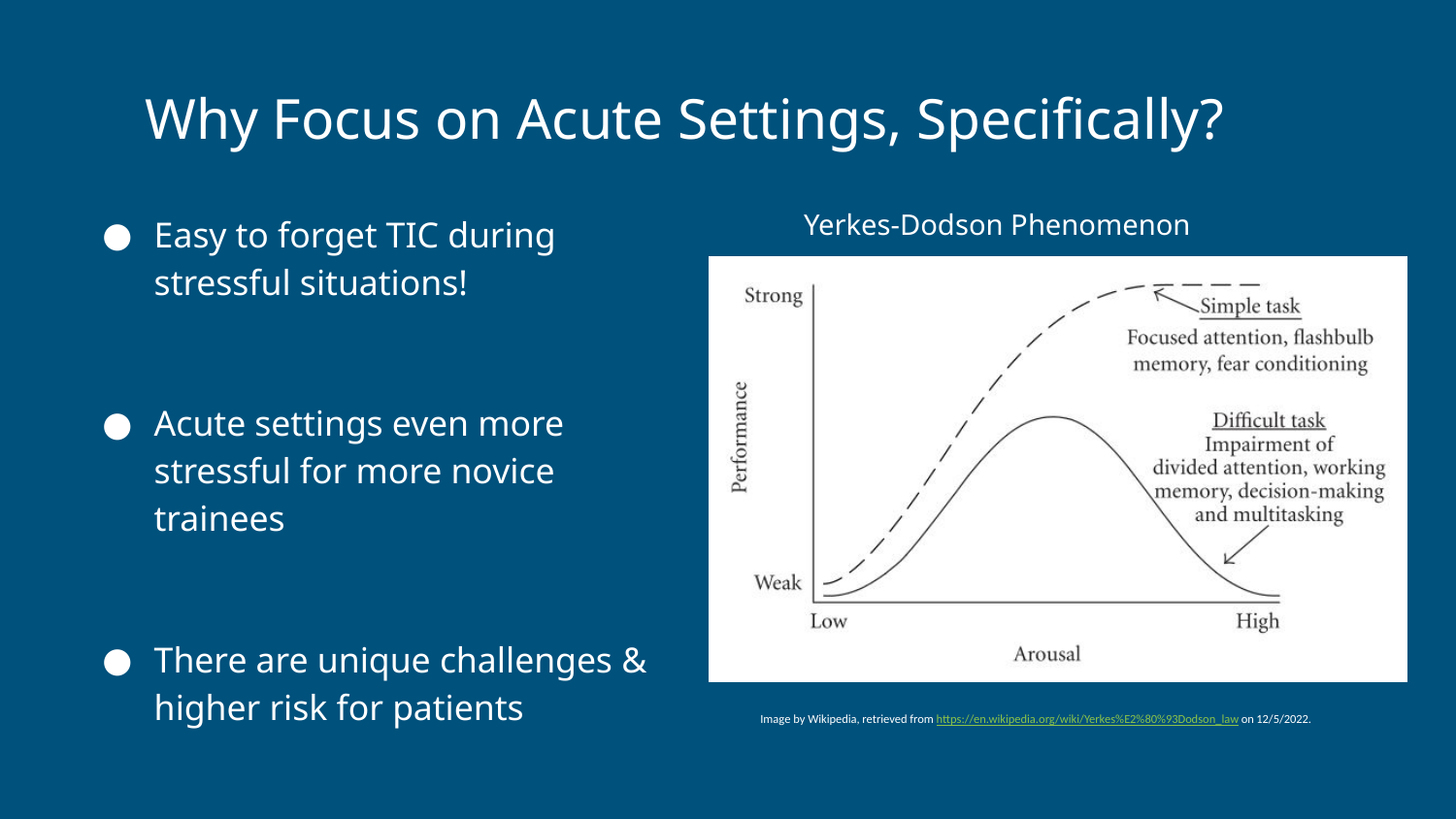

# Why Focus on Acute Settings, Specifically?
Easy to forget TIC during stressful situations!
Acute settings even more stressful for more novice trainees
There are unique challenges & higher risk for patients
Yerkes-Dodson Phenomenon
Image by Wikipedia, retrieved from https://en.wikipedia.org/wiki/Yerkes%E2%80%93Dodson_law on 12/5/2022.

## Slide 18
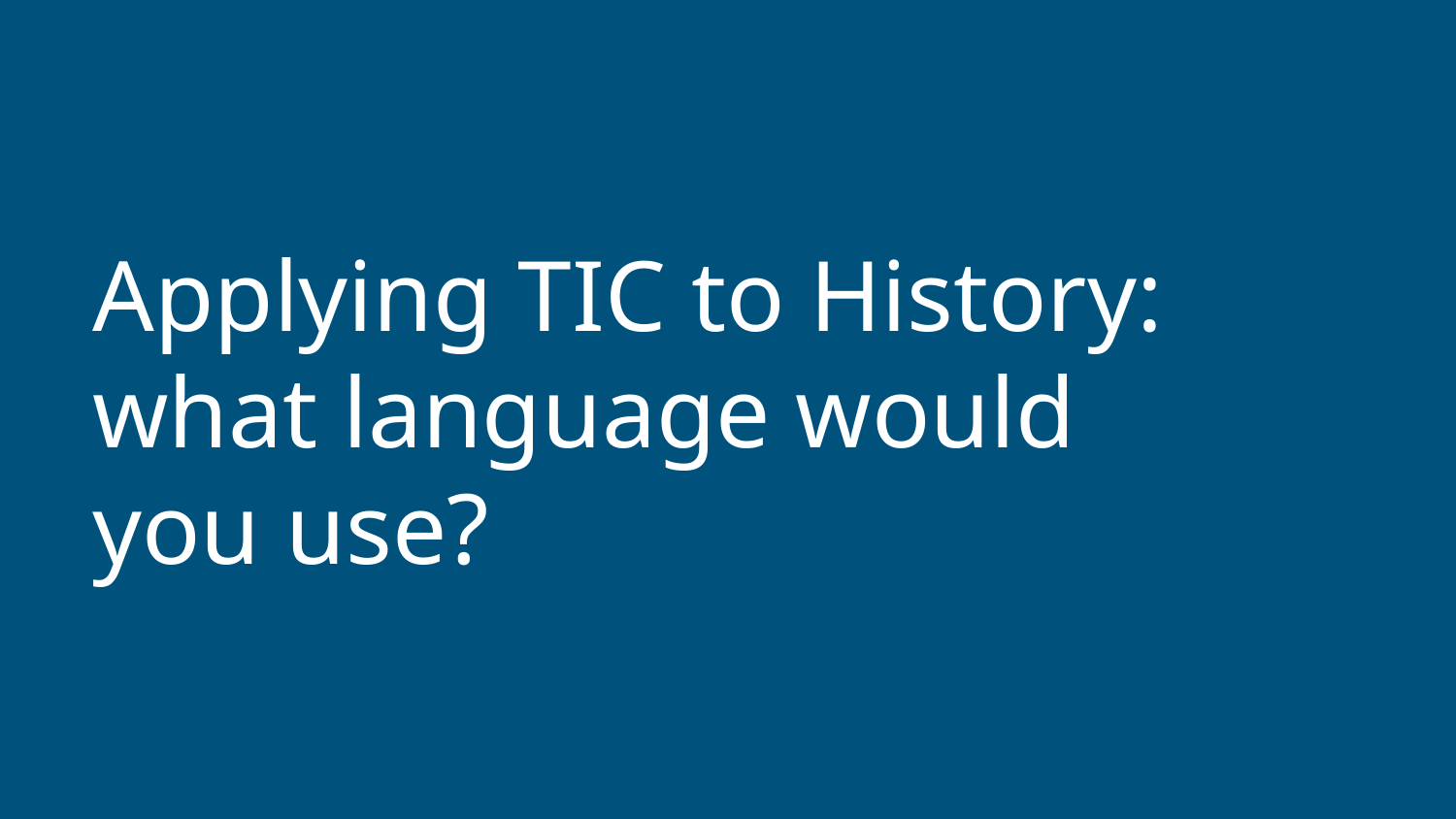

# Applying TIC to History: what language would you use?

## Slide 19
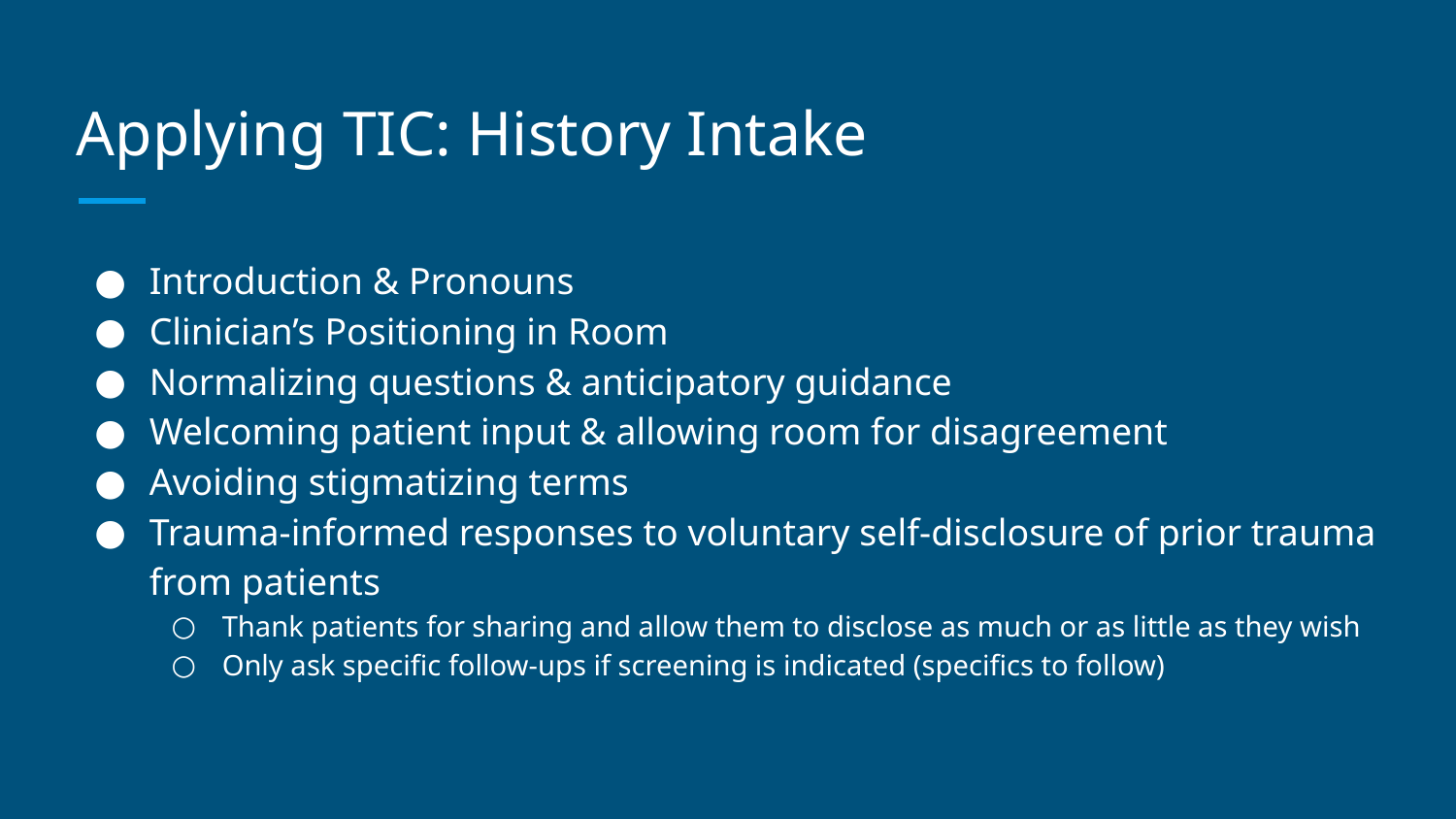

# Applying TIC: History Intake
Introduction & Pronouns
Clinician’s Positioning in Room
Normalizing questions & anticipatory guidance
Welcoming patient input & allowing room for disagreement
Avoiding stigmatizing terms
Trauma-informed responses to voluntary self-disclosure of prior trauma from patients
Thank patients for sharing and allow them to disclose as much or as little as they wish
Only ask specific follow-ups if screening is indicated (specifics to follow)

## Slide 20
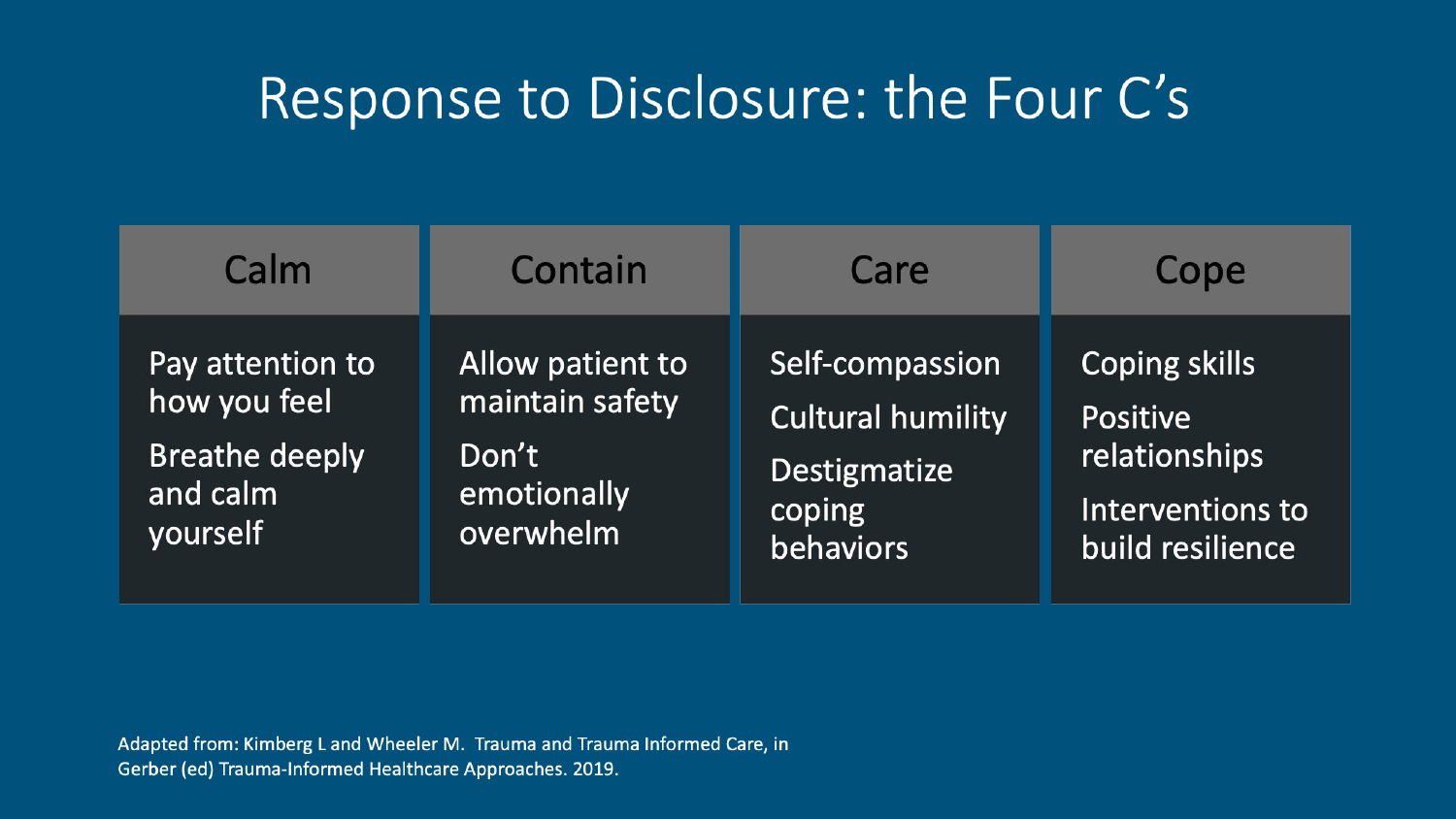

## Slide 21
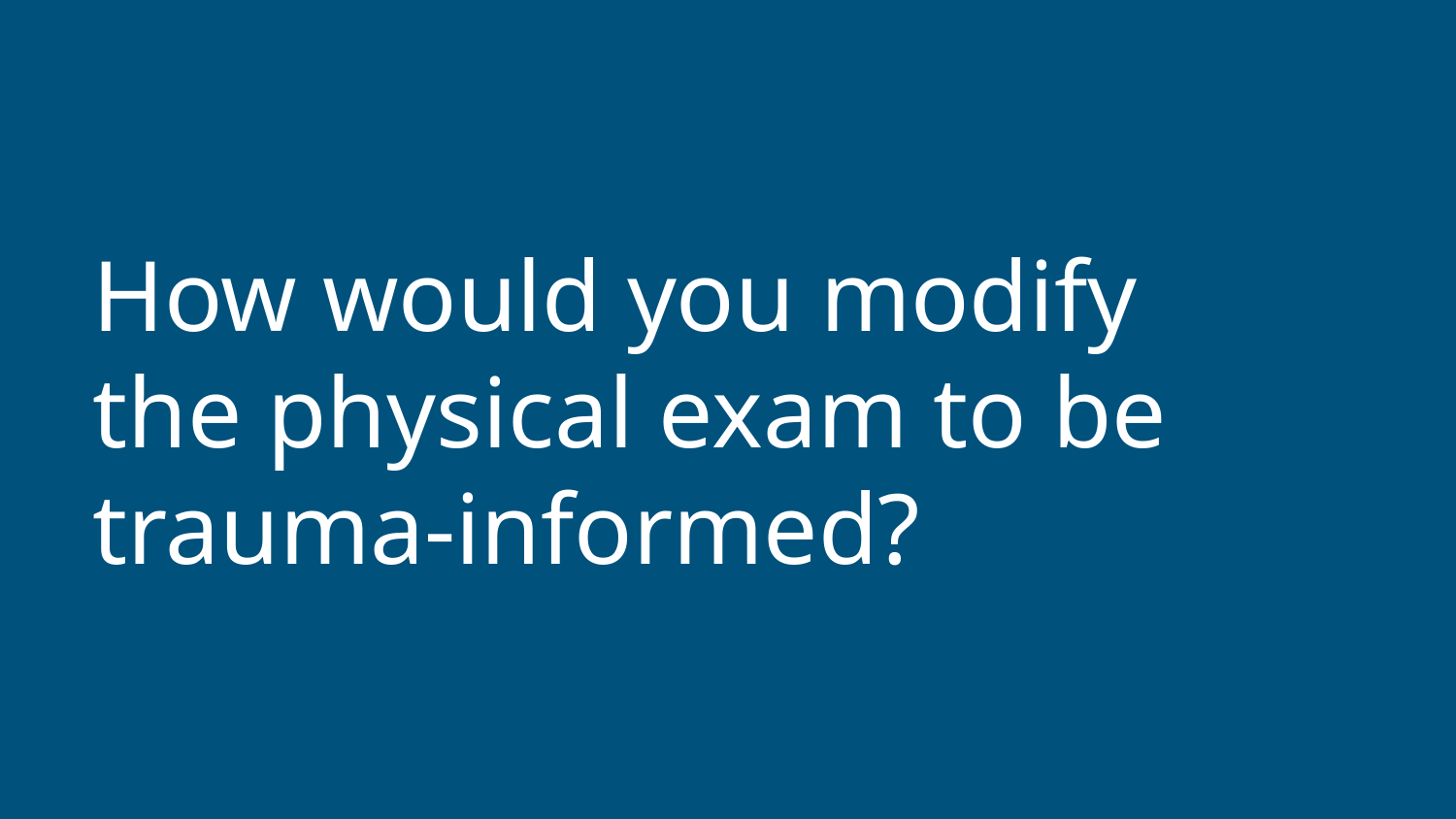

# How would you modify the physical exam to be trauma-informed?

## Slide 22
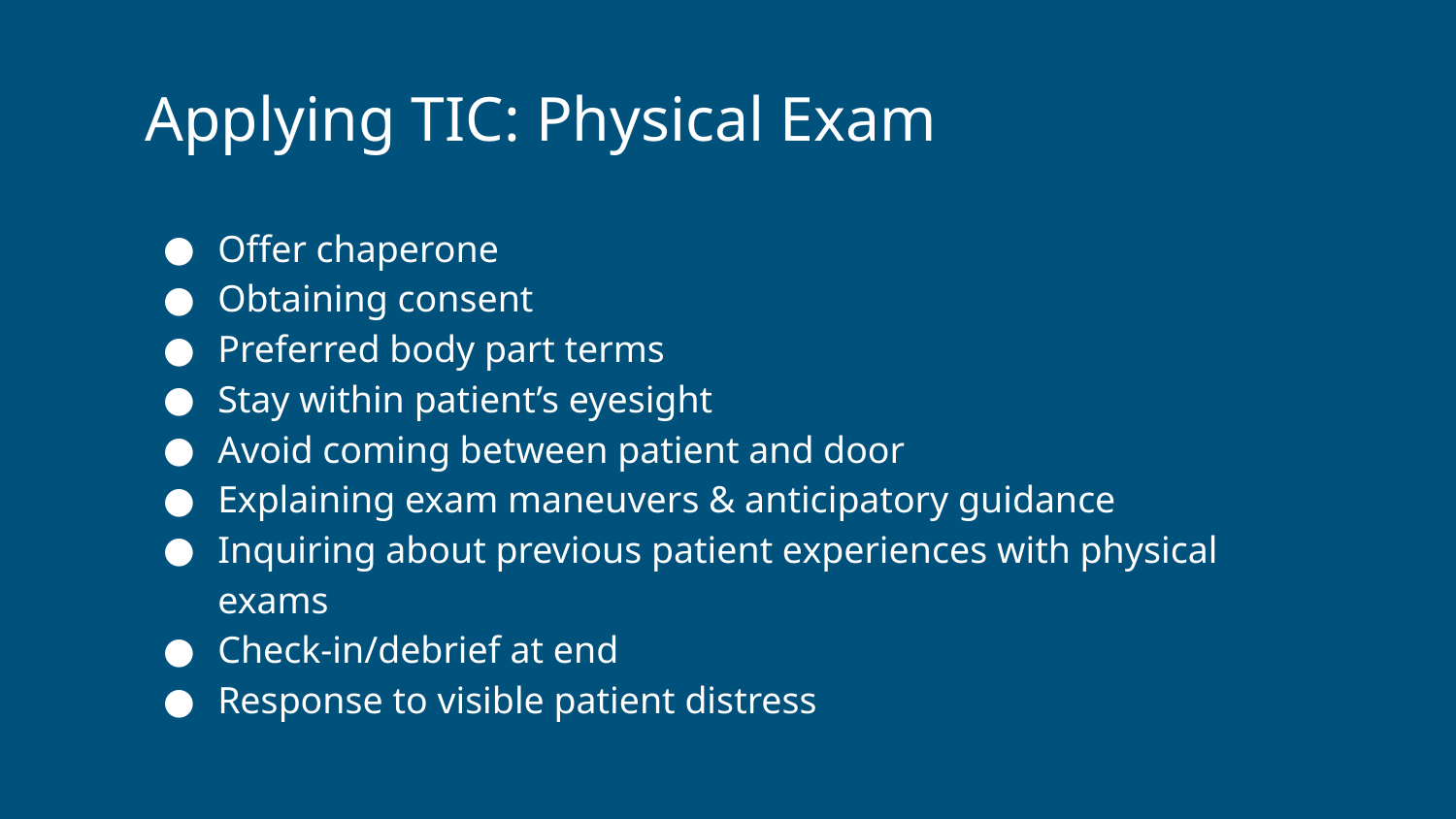

# Applying TIC: Physical Exam
Offer chaperone
Obtaining consent
Preferred body part terms
Stay within patient’s eyesight
Avoid coming between patient and door
Explaining exam maneuvers & anticipatory guidance
Inquiring about previous patient experiences with physical exams
Check-in/debrief at end
Response to visible patient distress

## Slide 23
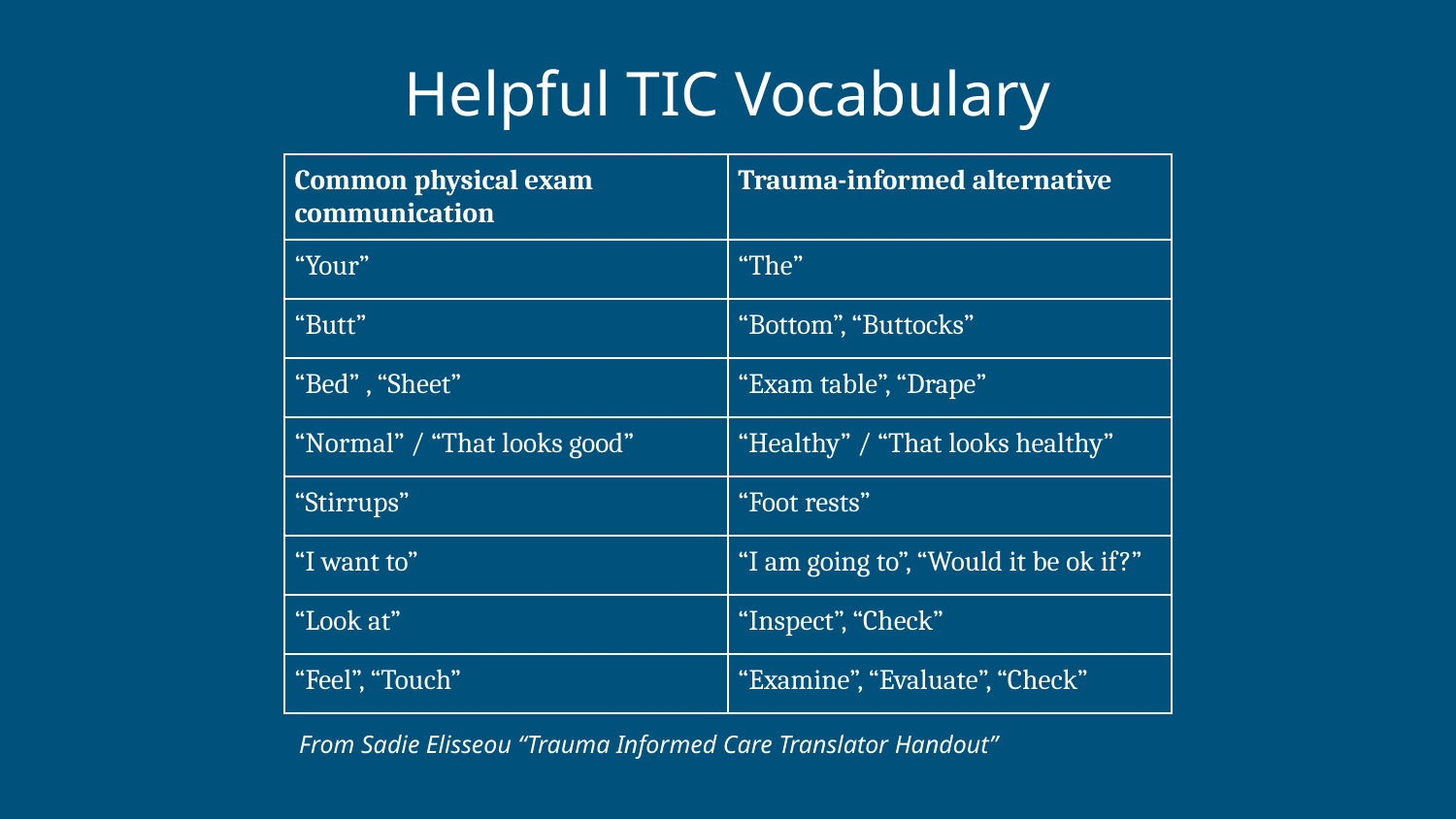

# Helpful TIC Vocabulary
| Common physical exam communication | Trauma-informed alternative |
| --- | --- |
| “Your” | “The” |
| “Butt” | “Bottom”, “Buttocks” |
| “Bed” , “Sheet” | “Exam table”, “Drape” |
| “Normal” / “That looks good” | “Healthy” / “That looks healthy” |
| “Stirrups” | “Foot rests” |
| “I want to” | “I am going to”, “Would it be ok if?” |
| “Look at” | “Inspect”, “Check” |
| “Feel”, “Touch” | “Examine”, “Evaluate”, “Check” |
From Sadie Elisseou “Trauma Informed Care Translator Handout”

## Slide 24
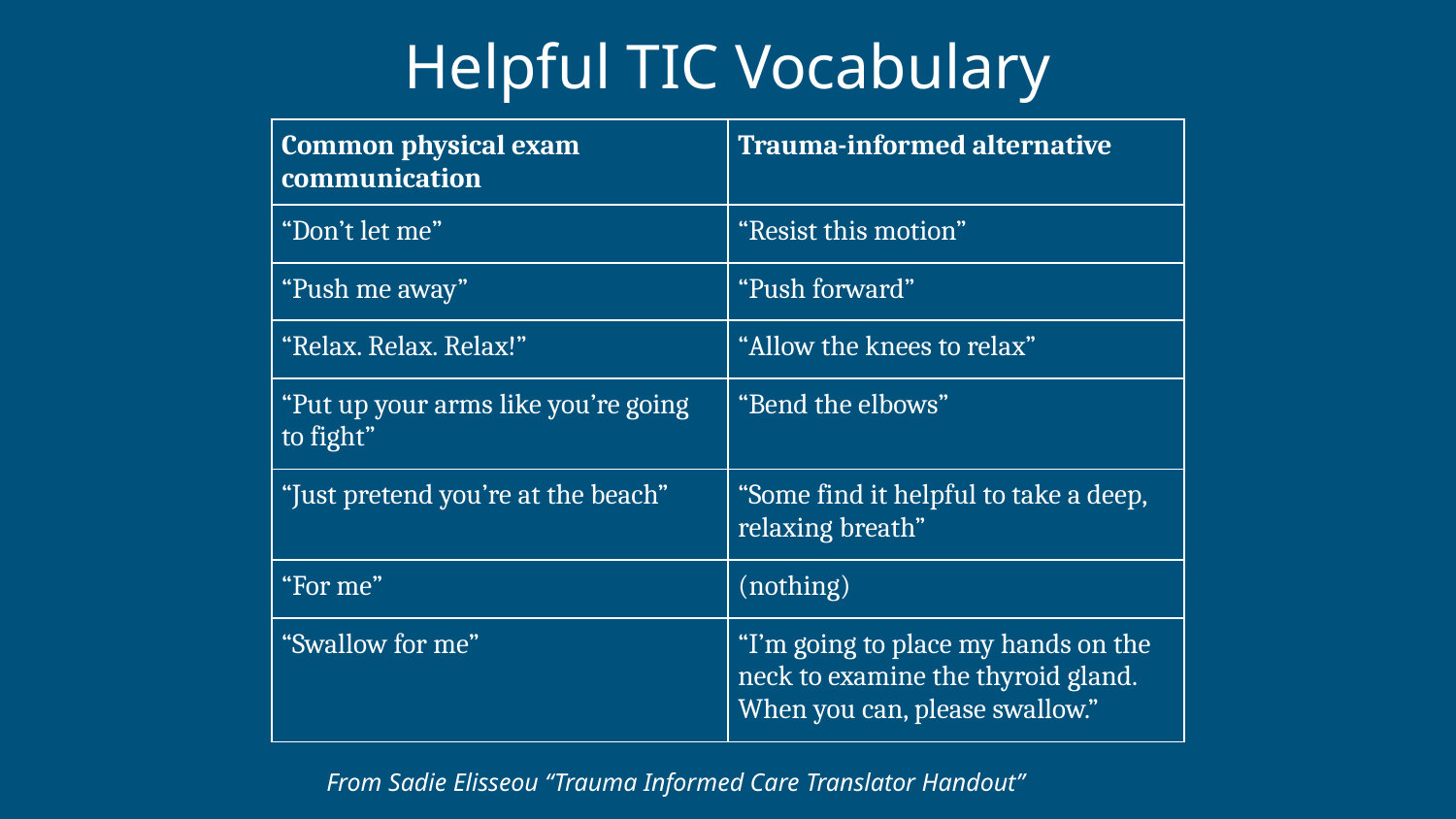

# Helpful TIC Vocabulary
| Common physical exam communication | Trauma-informed alternative |
| --- | --- |
| “Don’t let me” | “Resist this motion” |
| “Push me away” | “Push forward” |
| “Relax. Relax. Relax!” | “Allow the knees to relax” |
| “Put up your arms like you’re going to fight” | “Bend the elbows” |
| “Just pretend you’re at the beach” | “Some find it helpful to take a deep, relaxing breath” |
| “For me” | (nothing) |
| “Swallow for me” | “I’m going to place my hands on the neck to examine the thyroid gland. When you can, please swallow.” |
From Sadie Elisseou “Trauma Informed Care Translator Handout”

## Slide 25
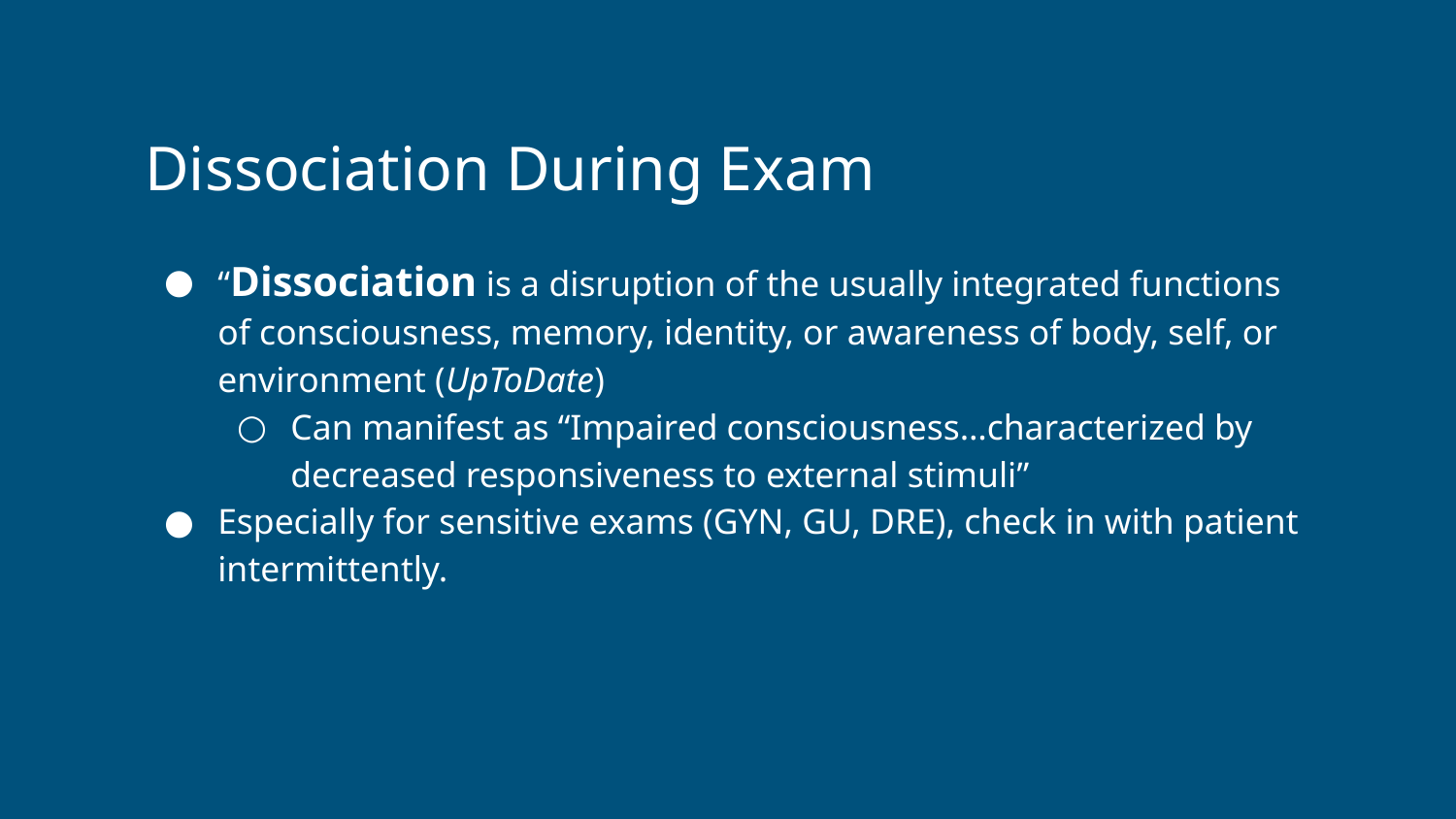

# Dissociation During Exam
“Dissociation is a disruption of the usually integrated functions of consciousness, memory, identity, or awareness of body, self, or environment (UpToDate)
Can manifest as “Impaired consciousness…characterized by decreased responsiveness to external stimuli”
Especially for sensitive exams (GYN, GU, DRE), check in with patient intermittently.

## Slide 26
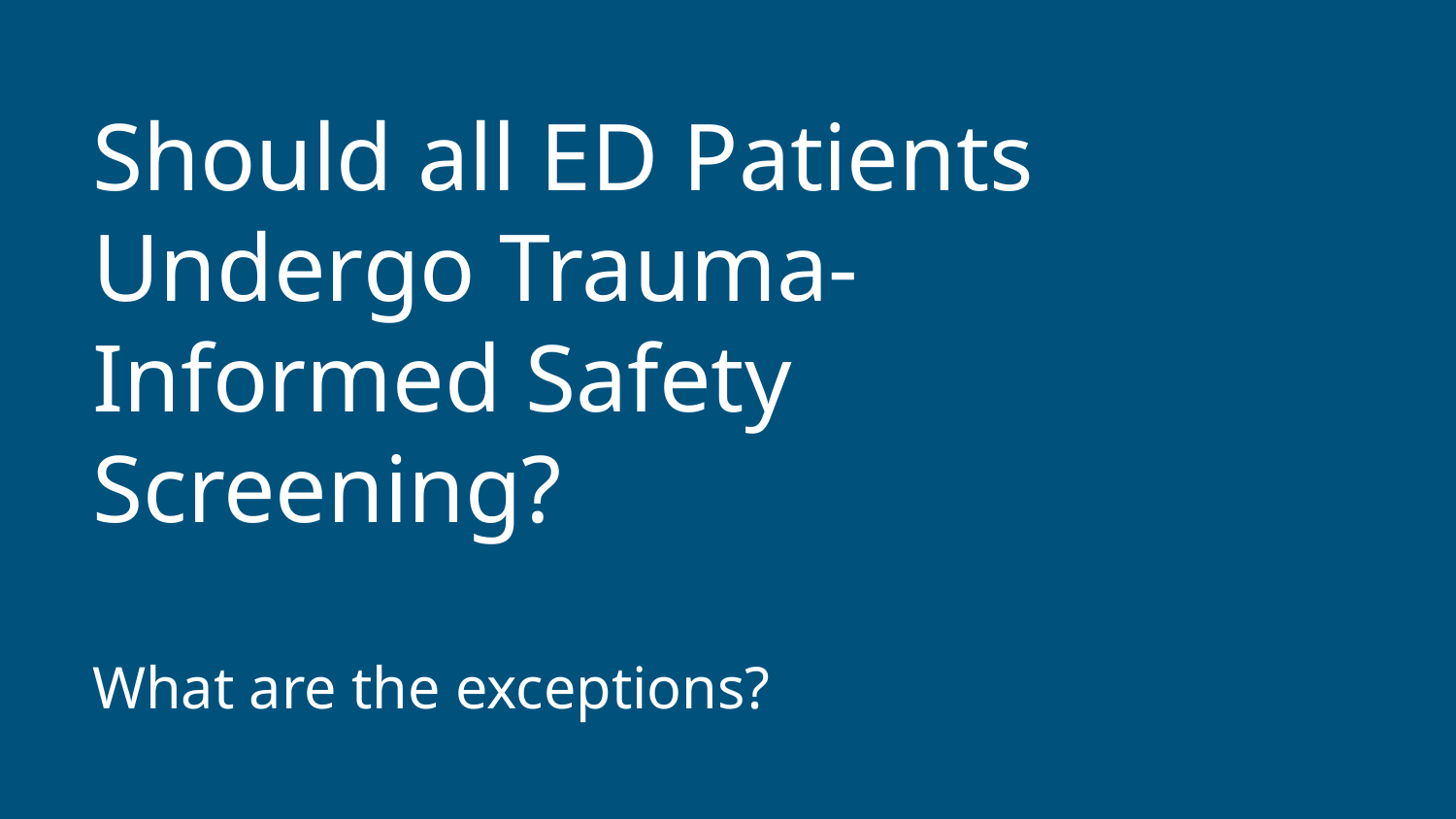

# Should all ED Patients Undergo Trauma-Informed Safety Screening?
What are the exceptions?

## Slide 27
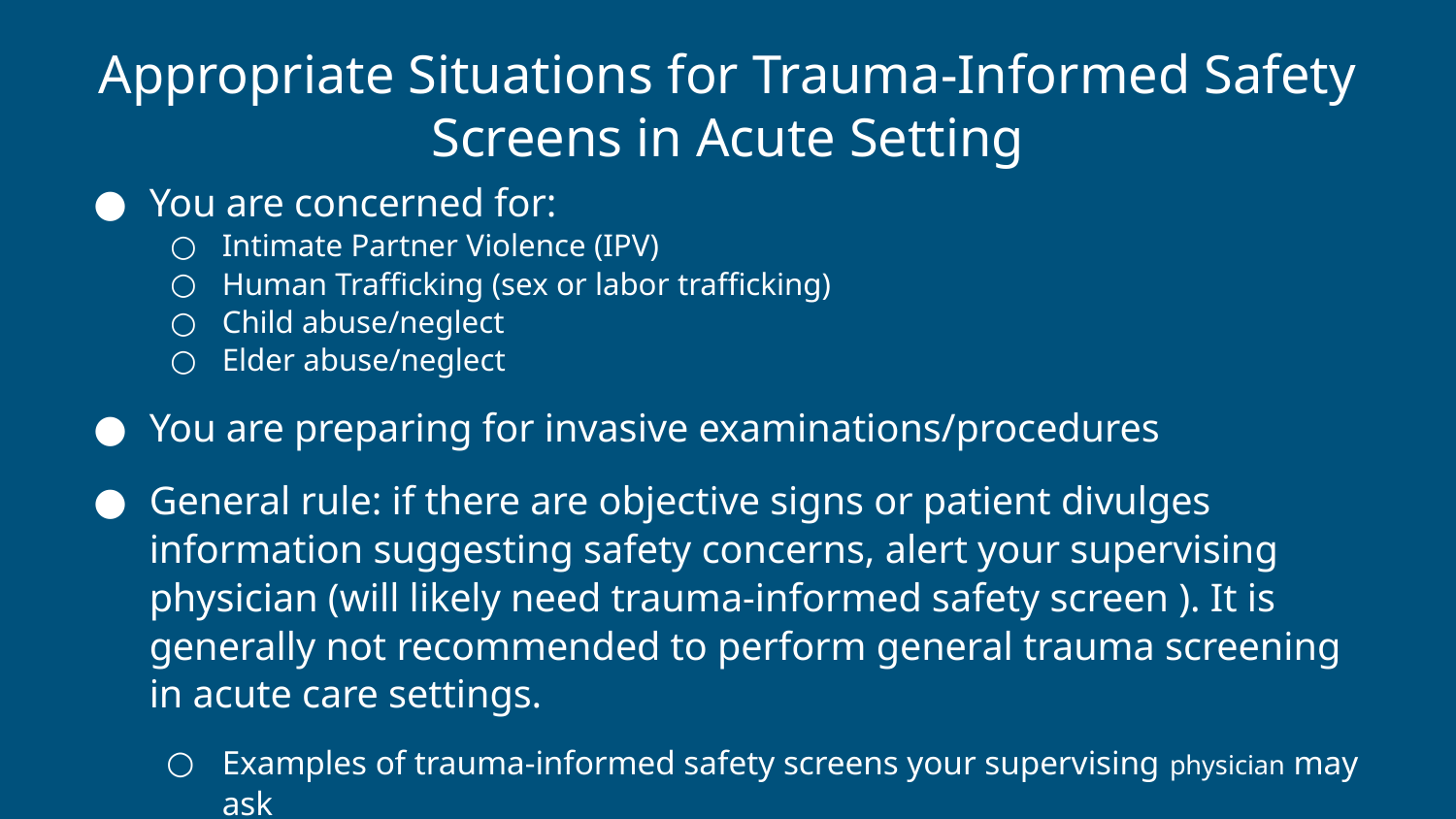

# Appropriate Situations for Trauma-Informed Safety Screens in Acute Setting
You are concerned for:
Intimate Partner Violence (IPV)
Human Trafficking (sex or labor trafficking)
Child abuse/neglect
Elder abuse/neglect
You are preparing for invasive examinations/procedures
General rule: if there are objective signs or patient divulges information suggesting safety concerns, alert your supervising physician (will likely need trauma-informed safety screen ). It is generally not recommended to perform general trauma screening in acute care settings.
Examples of trauma-informed safety screens your supervising physician may ask

## Slide 28
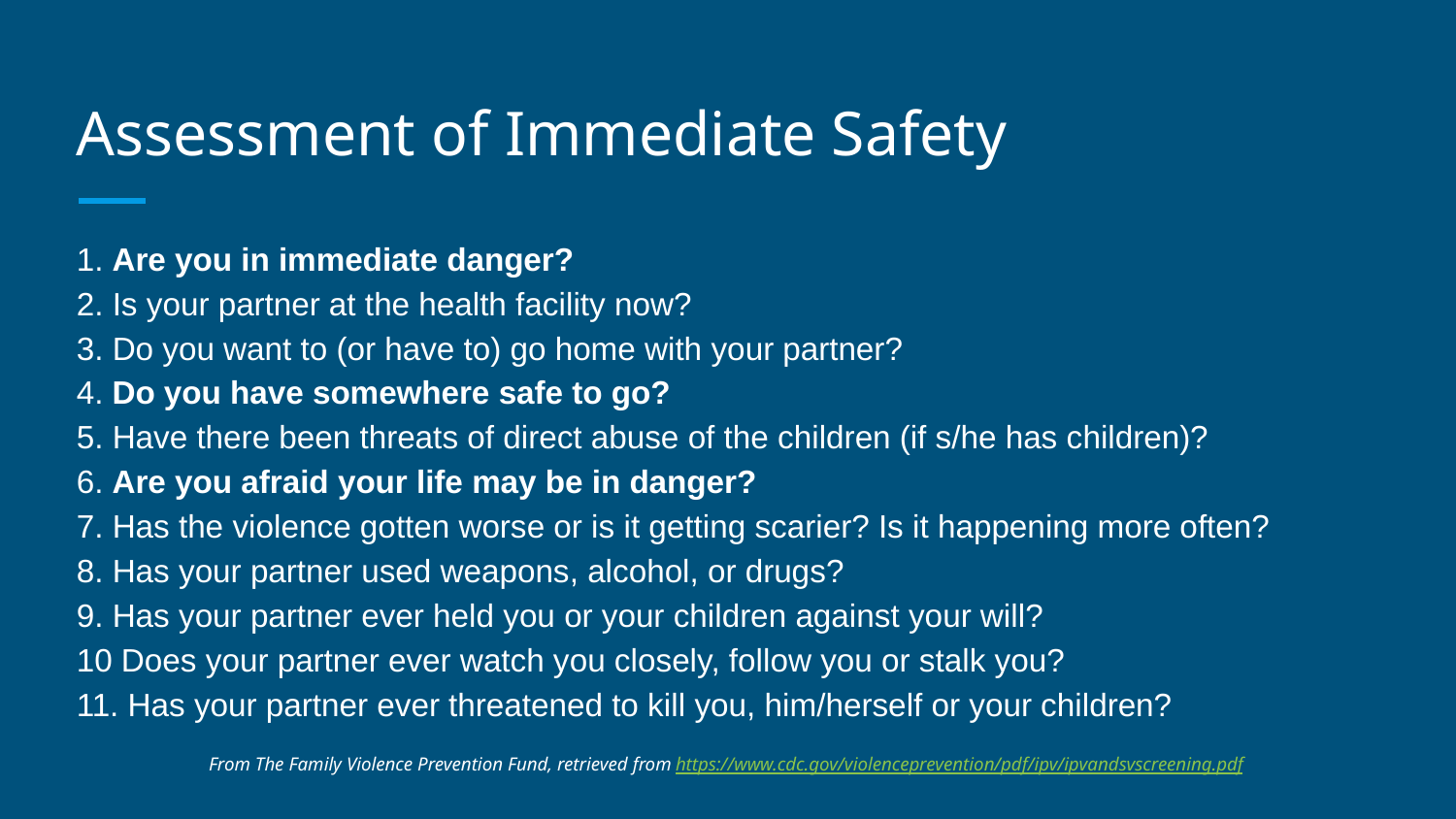

# Assessment of Immediate Safety
1. Are you in immediate danger?
2. Is your partner at the health facility now?
3. Do you want to (or have to) go home with your partner?
4. Do you have somewhere safe to go?
5. Have there been threats of direct abuse of the children (if s/he has children)?
6. Are you afraid your life may be in danger?
7. Has the violence gotten worse or is it getting scarier? Is it happening more often?
8. Has your partner used weapons, alcohol, or drugs?
9. Has your partner ever held you or your children against your will?
10 Does your partner ever watch you closely, follow you or stalk you?
11. Has your partner ever threatened to kill you, him/herself or your children?
From The Family Violence Prevention Fund, retrieved from https://www.cdc.gov/violenceprevention/pdf/ipv/ipvandsvscreening.pdf

## Slide 29
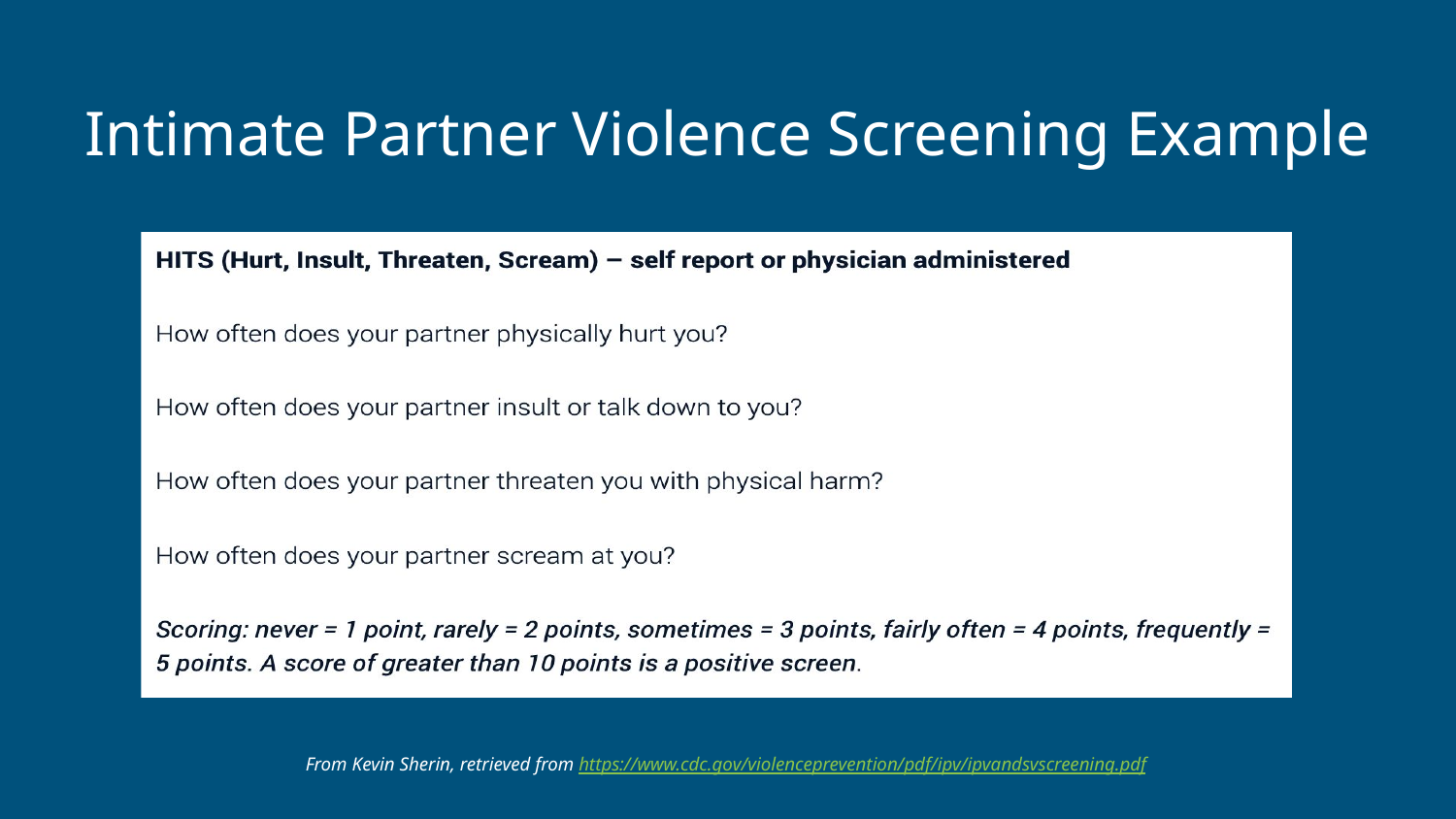

# Intimate Partner Violence Screening Example
From Kevin Sherin, retrieved from https://www.cdc.gov/violenceprevention/pdf/ipv/ipvandsvscreening.pdf

## Slide 30
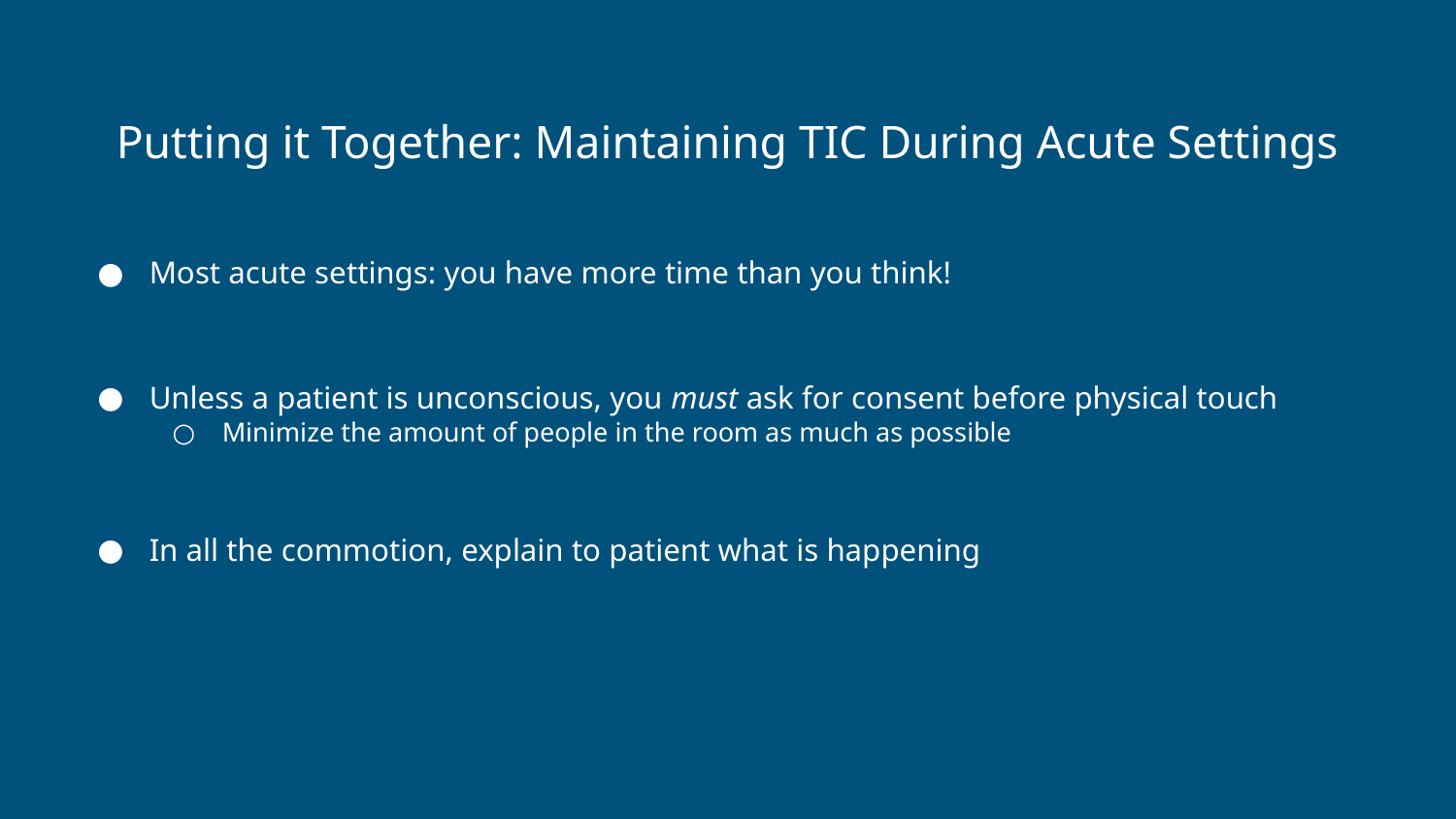

# Putting it Together: Maintaining TIC During Acute Settings
Most acute settings: you have more time than you think!
Unless a patient is unconscious, you must ask for consent before physical touch
Minimize the amount of people in the room as much as possible
In all the commotion, explain to patient what is happening

## Slide 31
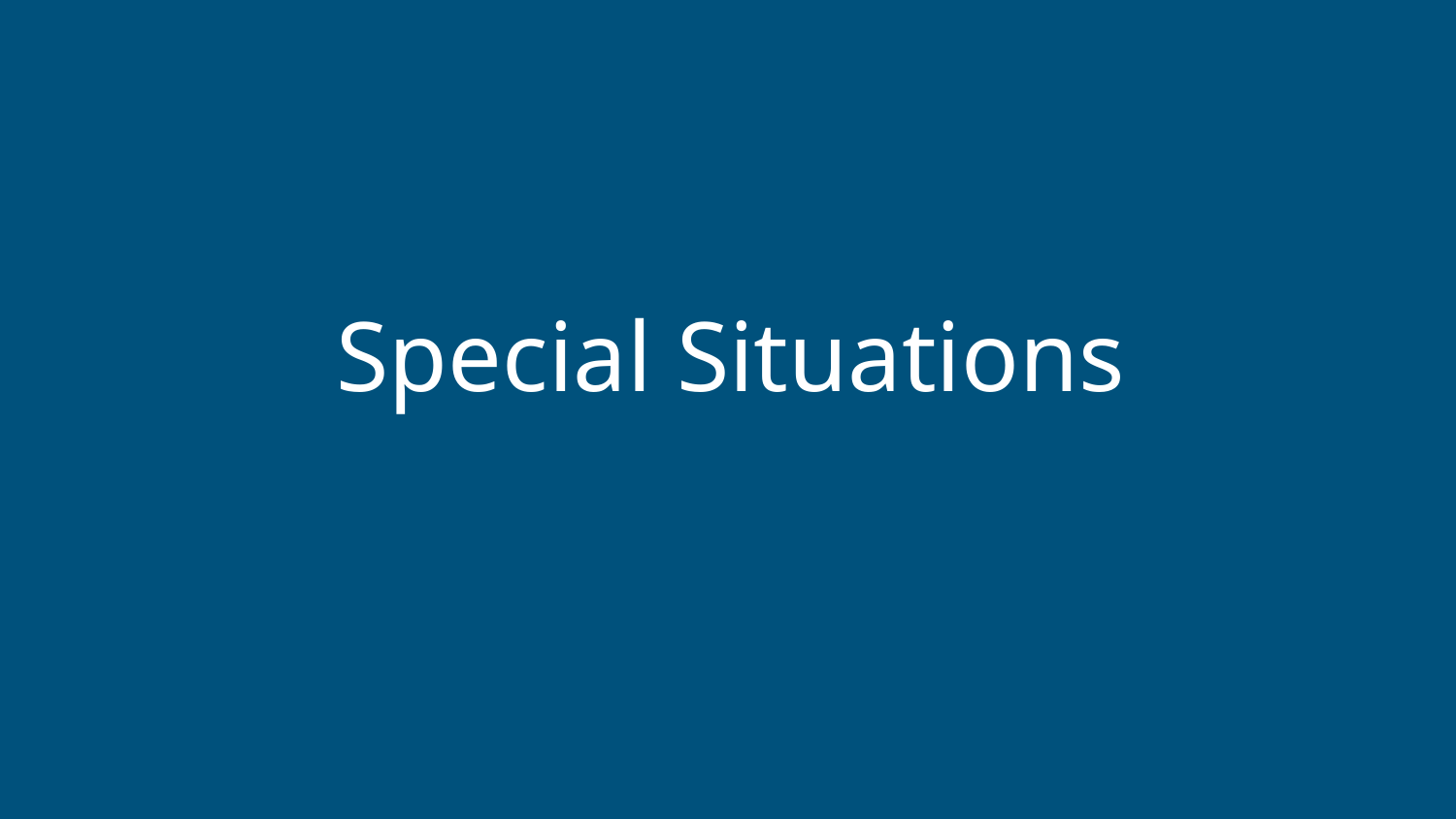

# Special Situations

## Slide 32
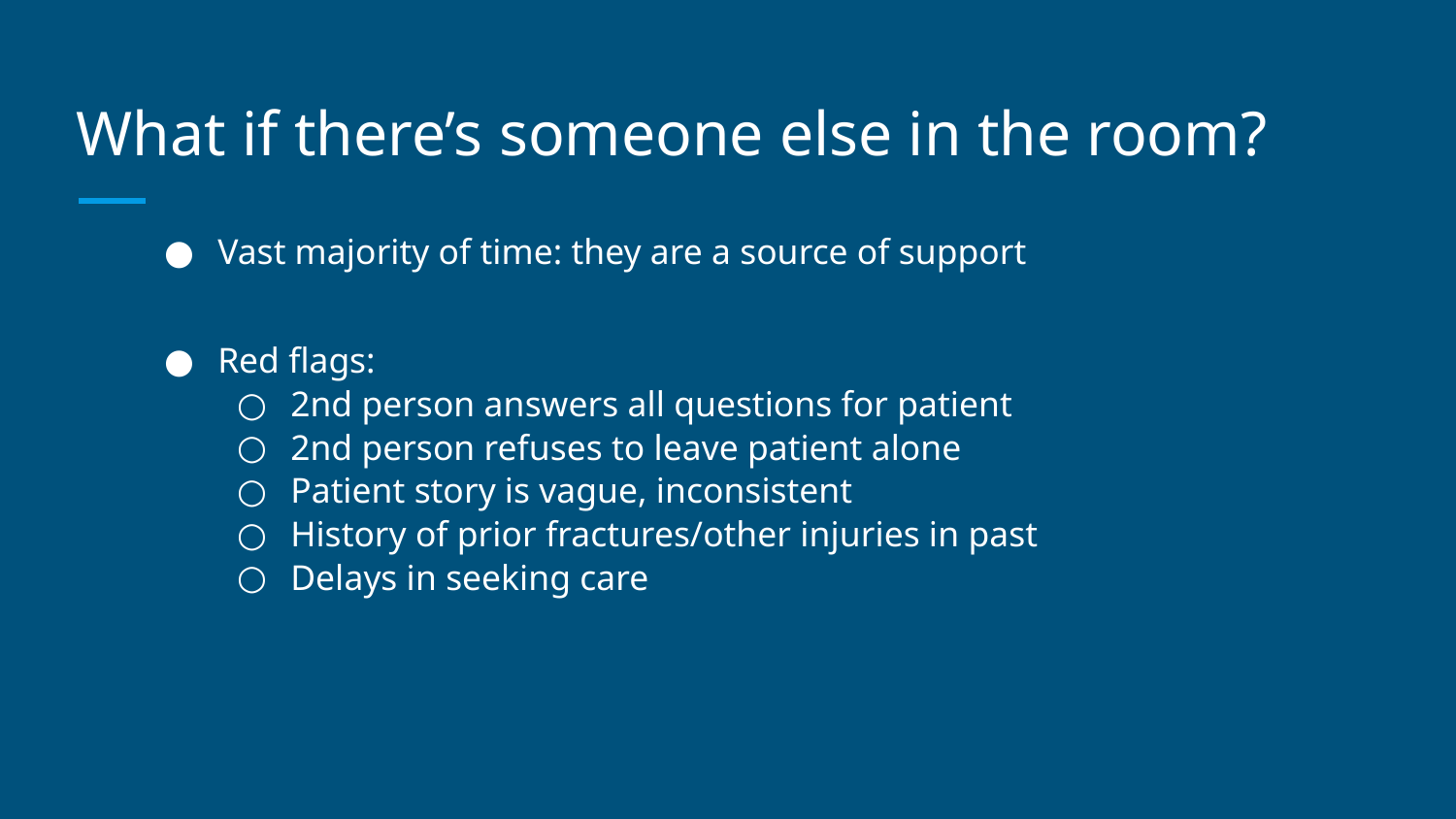

# What if there’s someone else in the room?
Vast majority of time: they are a source of support
Red flags:
2nd person answers all questions for patient
2nd person refuses to leave patient alone
Patient story is vague, inconsistent
History of prior fractures/other injuries in past
Delays in seeking care

## Slide 33
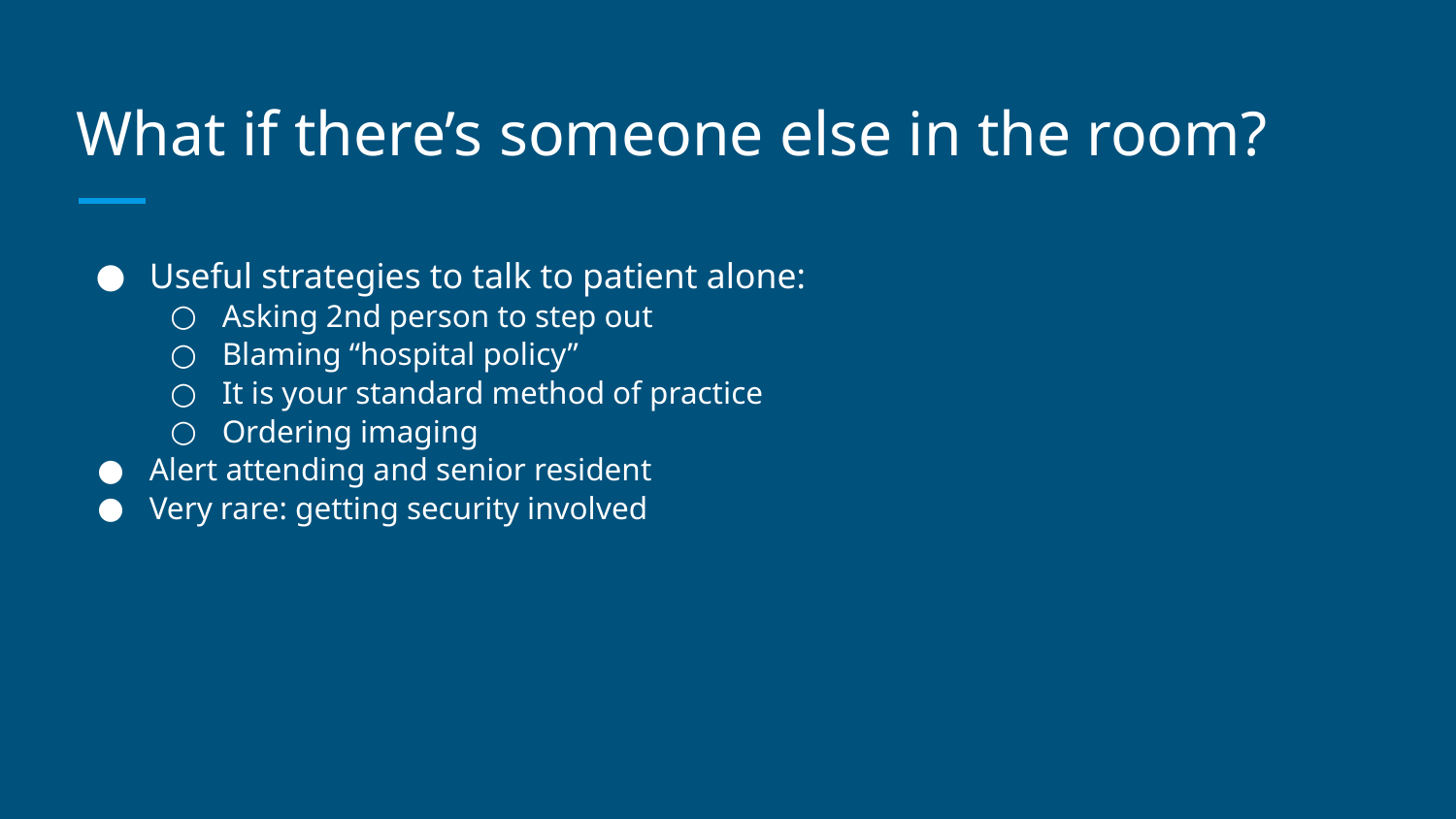

# What if there’s someone else in the room?
Useful strategies to talk to patient alone:
Asking 2nd person to step out
Blaming “hospital policy”
It is your standard method of practice
Ordering imaging
Alert attending and senior resident
Very rare: getting security involved

## Slide 34
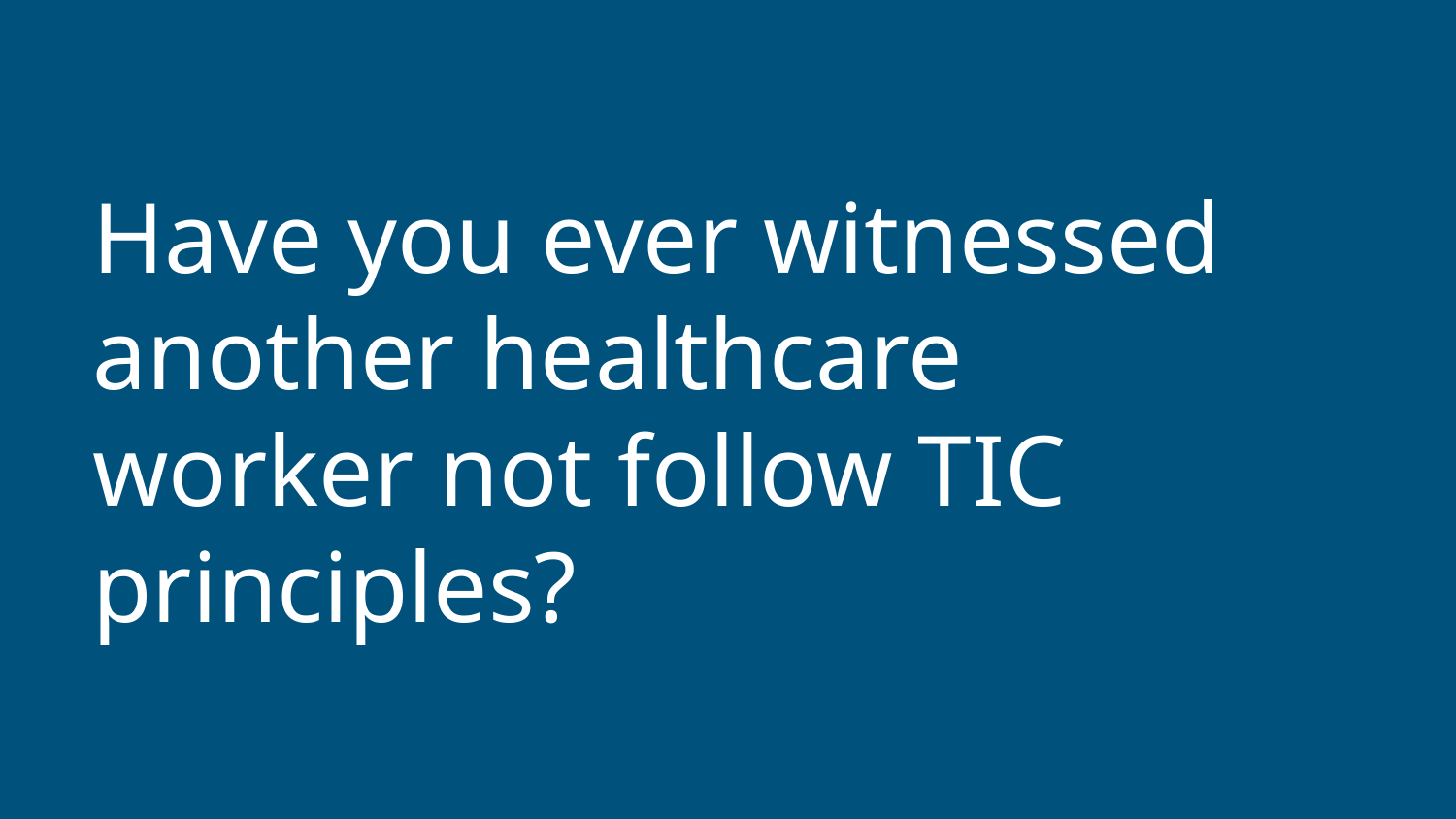

# Have you ever witnessed another healthcare worker not follow TIC principles?

## Slide 35
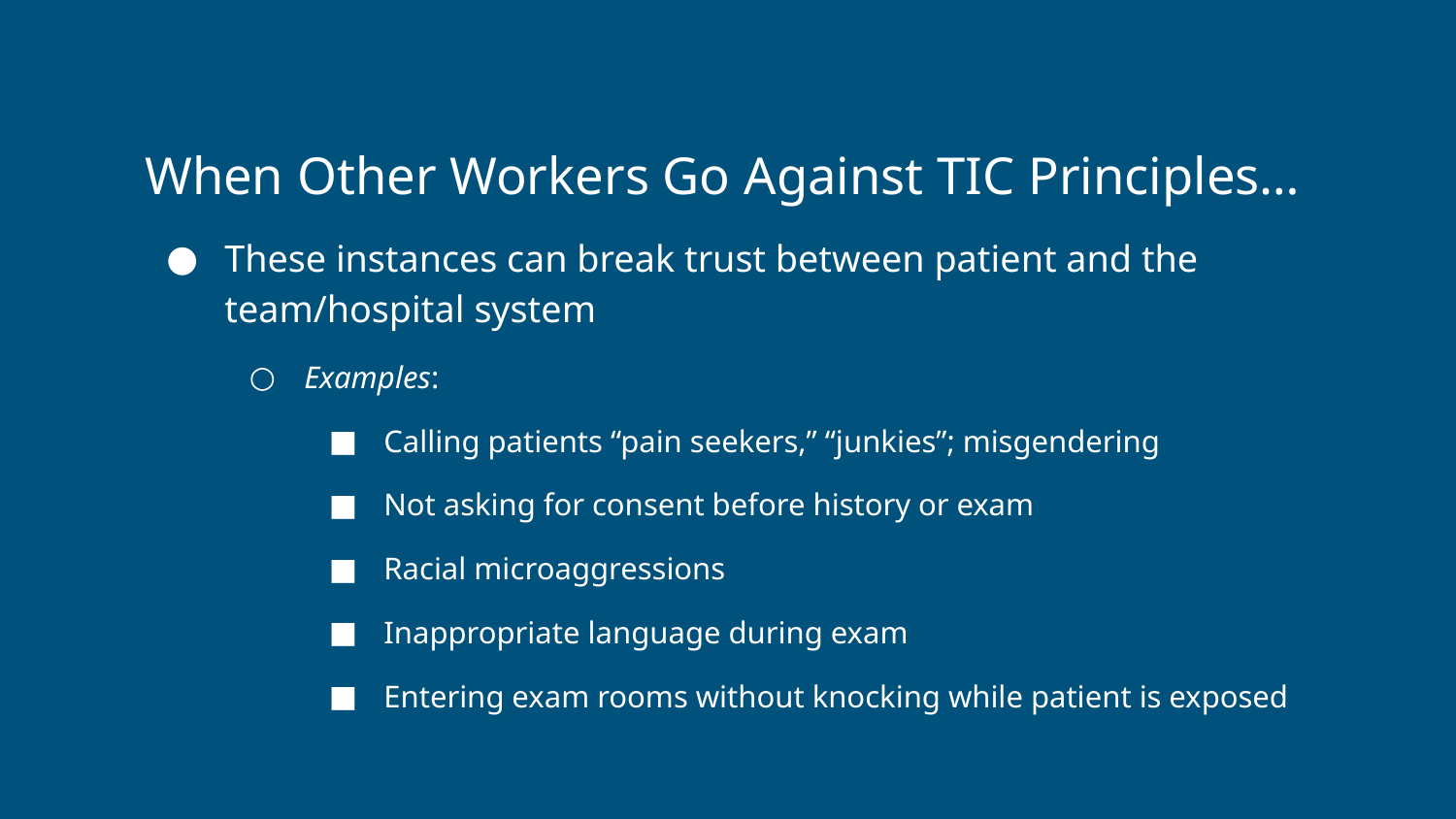

# When Other Workers Go Against TIC Principles…
These instances can break trust between patient and the team/hospital system
Examples:
Calling patients “pain seekers,” “junkies”; misgendering
Not asking for consent before history or exam
Racial microaggressions
Inappropriate language during exam
Entering exam rooms without knocking while patient is exposed

## Slide 36
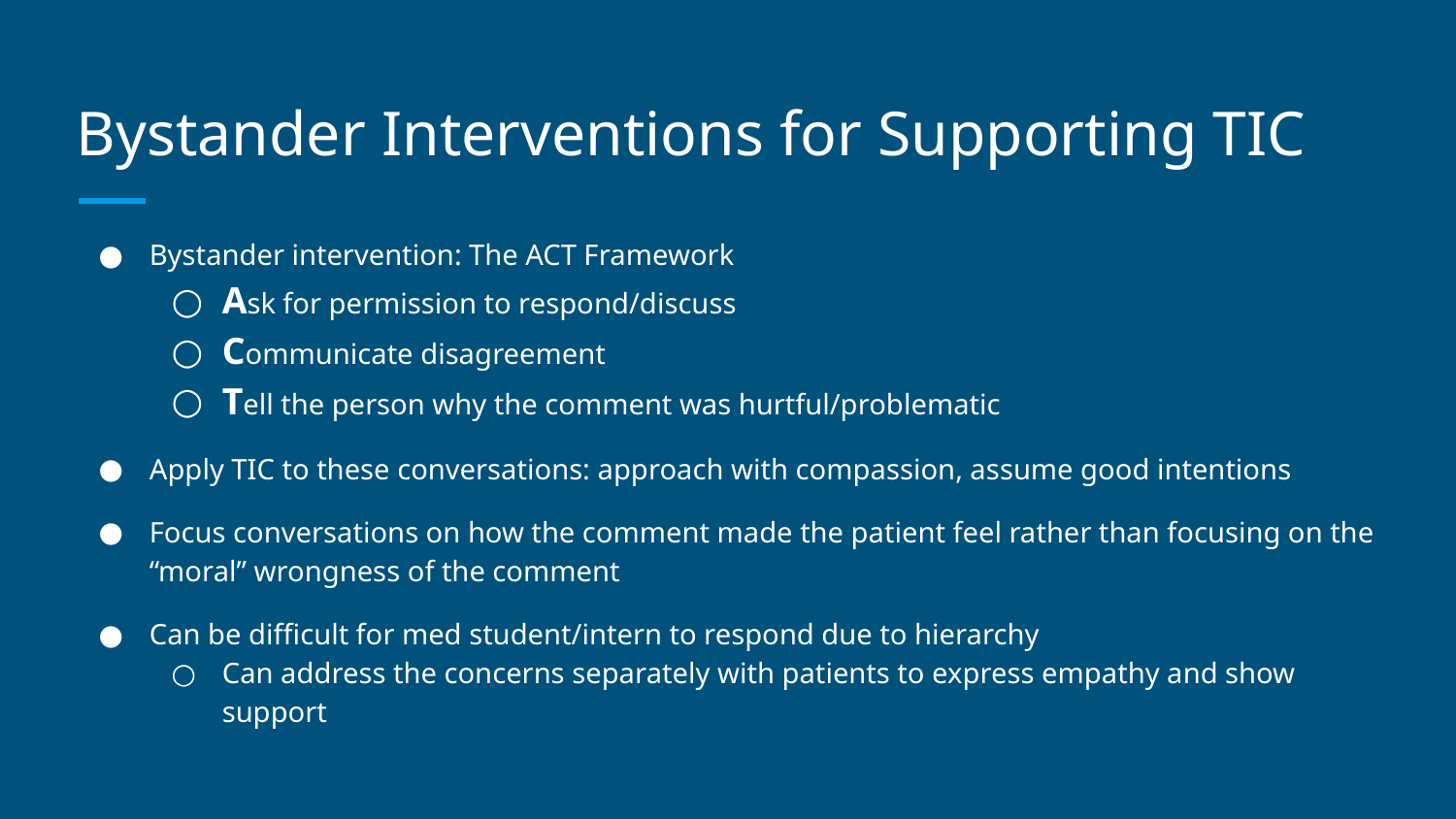

# Bystander Interventions for Supporting TIC
Bystander intervention: The ACT Framework
Ask for permission to respond/discuss
Communicate disagreement
Tell the person why the comment was hurtful/problematic
Apply TIC to these conversations: approach with compassion, assume good intentions
Focus conversations on how the comment made the patient feel rather than focusing on the “moral” wrongness of the comment
Can be difficult for med student/intern to respond due to hierarchy
Can address the concerns separately with patients to express empathy and show support

## Slide 37
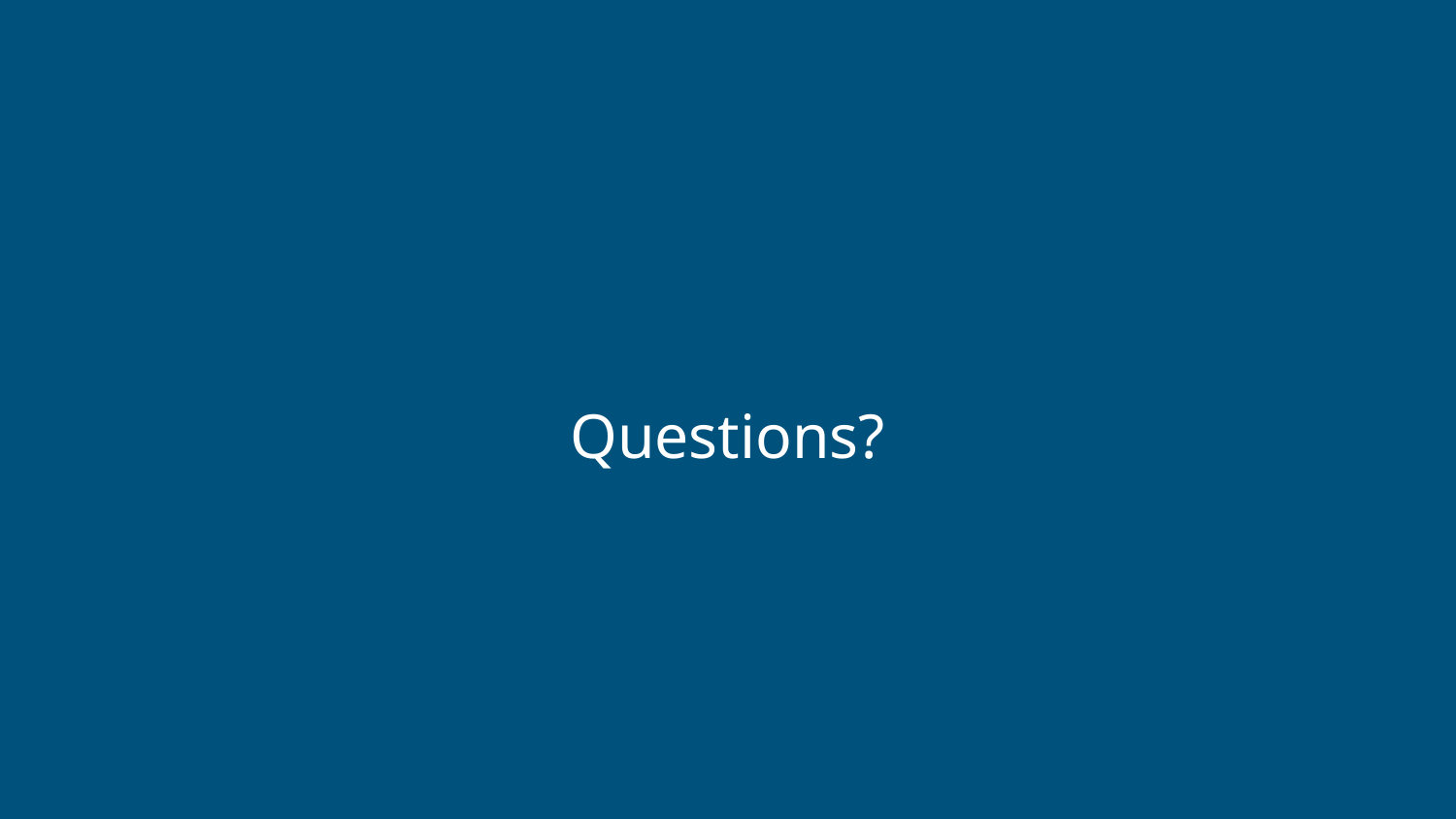

# Questions?

## Slide 38
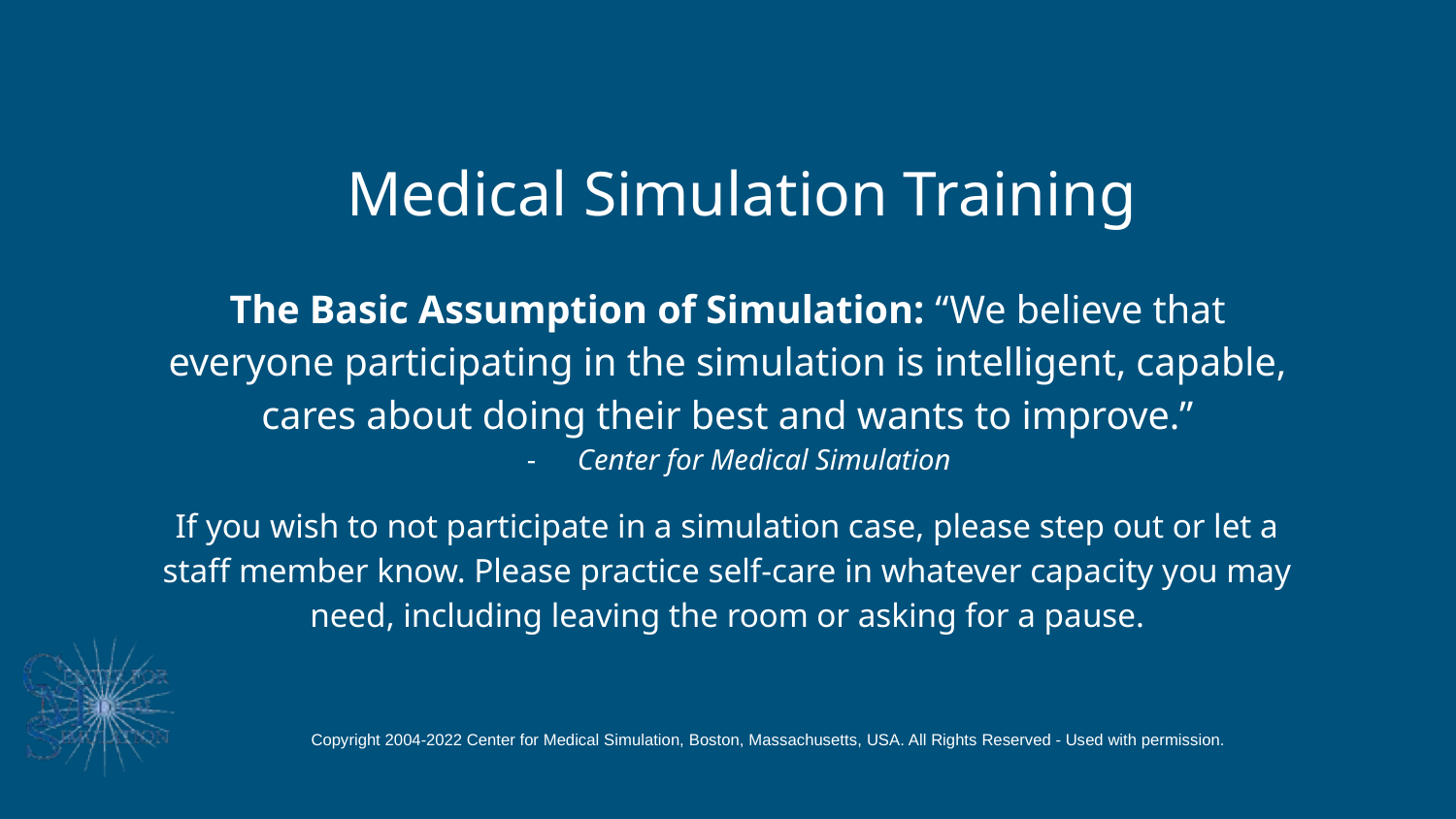

# Medical Simulation Training
The Basic Assumption of Simulation: “We believe that everyone participating in the simulation is intelligent, capable, cares about doing their best and wants to improve.”
Center for Medical Simulation
If you wish to not participate in a simulation case, please step out or let a staff member know. Please practice self-care in whatever capacity you may need, including leaving the room or asking for a pause.
Copyright 2004-2022 Center for Medical Simulation, Boston, Massachusetts, USA. All Rights Reserved - Used with permission.

## Slide 39
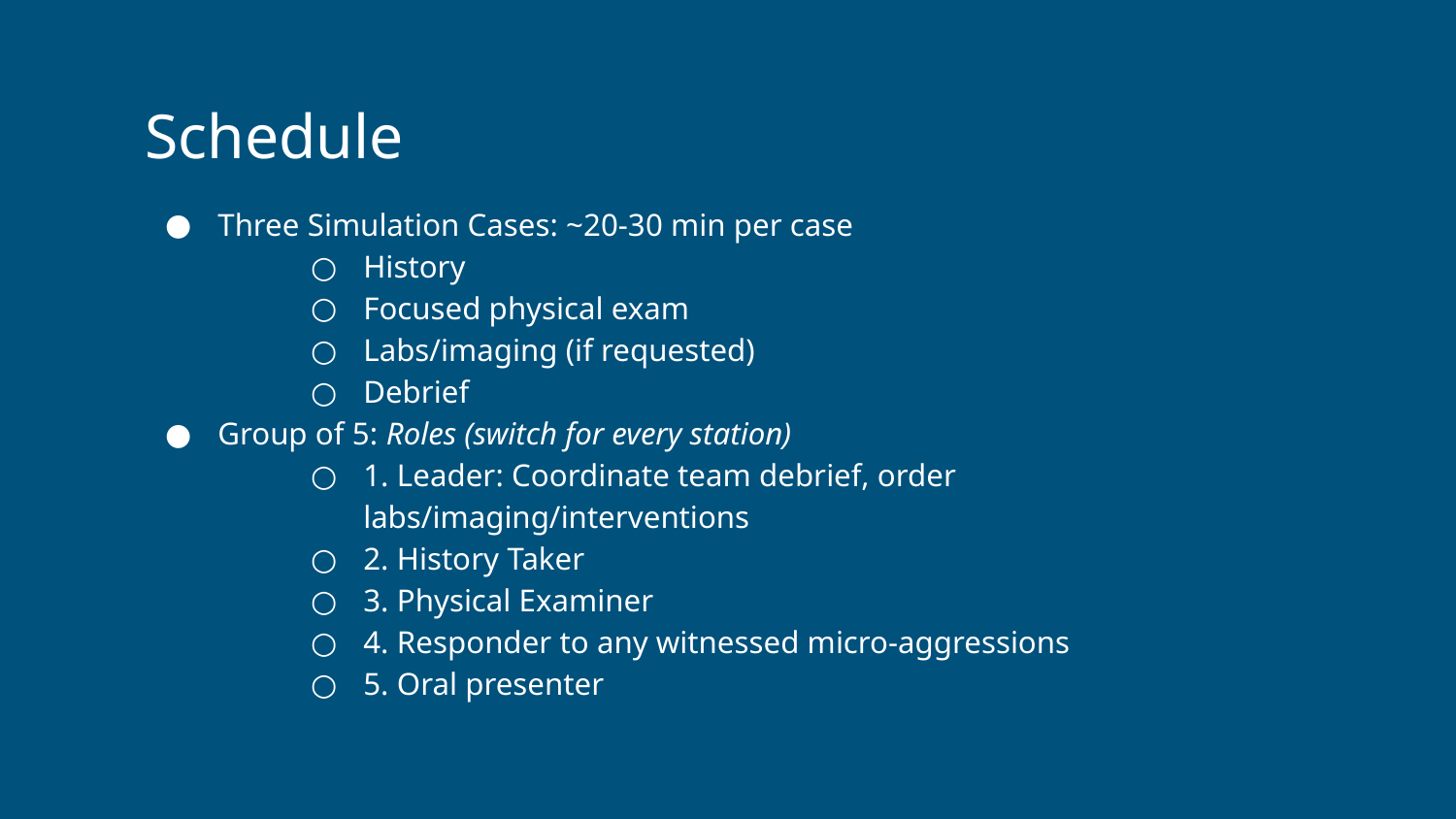

# Schedule
Three Simulation Cases: ~20-30 min per case
History
Focused physical exam
Labs/imaging (if requested)
Debrief
Group of 5: Roles (switch for every station)
1. Leader: Coordinate team debrief, order labs/imaging/interventions
2. History Taker
3. Physical Examiner
4. Responder to any witnessed micro-aggressions
5. Oral presenter

## Slide 40
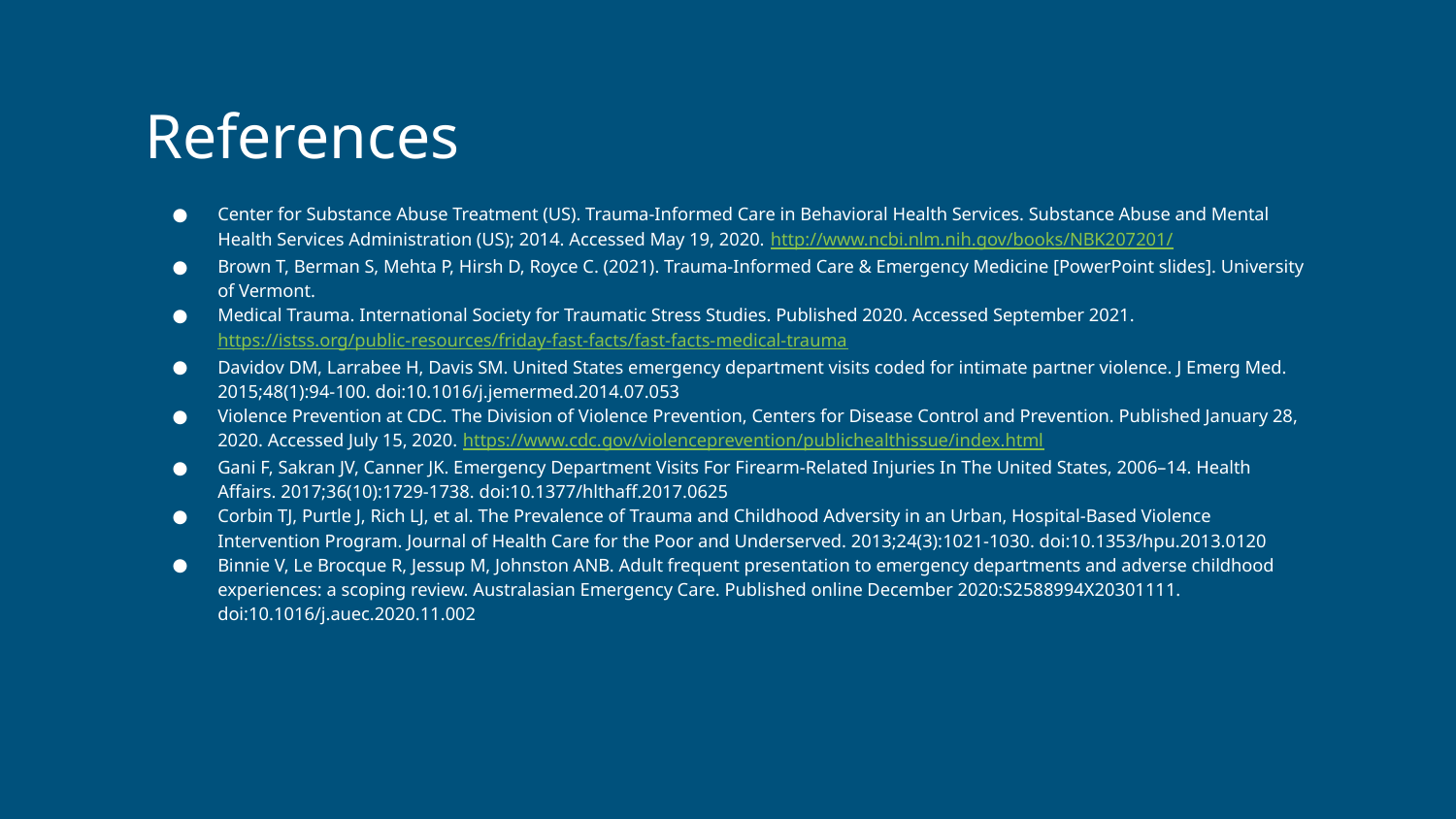

# References
Center for Substance Abuse Treatment (US). Trauma-Informed Care in Behavioral Health Services. Substance Abuse and Mental Health Services Administration (US); 2014. Accessed May 19, 2020. http://www.ncbi.nlm.nih.gov/books/NBK207201/
Brown T, Berman S, Mehta P, Hirsh D, Royce C. (2021). Trauma-Informed Care & Emergency Medicine [PowerPoint slides]. University of Vermont.
Medical Trauma. International Society for Traumatic Stress Studies. Published 2020. Accessed September 2021. https://istss.org/public-resources/friday-fast-facts/fast-facts-medical-trauma
Davidov DM, Larrabee H, Davis SM. United States emergency department visits coded for intimate partner violence. J Emerg Med. 2015;48(1):94-100. doi:10.1016/j.jemermed.2014.07.053
Violence Prevention at CDC. The Division of Violence Prevention, Centers for Disease Control and Prevention. Published January 28, 2020. Accessed July 15, 2020. https://www.cdc.gov/violenceprevention/publichealthissue/index.html
Gani F, Sakran JV, Canner JK. Emergency Department Visits For Firearm-Related Injuries In The United States, 2006–14. Health Affairs. 2017;36(10):1729-1738. doi:10.1377/hlthaff.2017.0625
Corbin TJ, Purtle J, Rich LJ, et al. The Prevalence of Trauma and Childhood Adversity in an Urban, Hospital-Based Violence Intervention Program. Journal of Health Care for the Poor and Underserved. 2013;24(3):1021-1030. doi:10.1353/hpu.2013.0120
Binnie V, Le Brocque R, Jessup M, Johnston ANB. Adult frequent presentation to emergency departments and adverse childhood experiences: a scoping review. Australasian Emergency Care. Published online December 2020:S2588994X20301111. doi:10.1016/j.auec.2020.11.002

## Slide 41
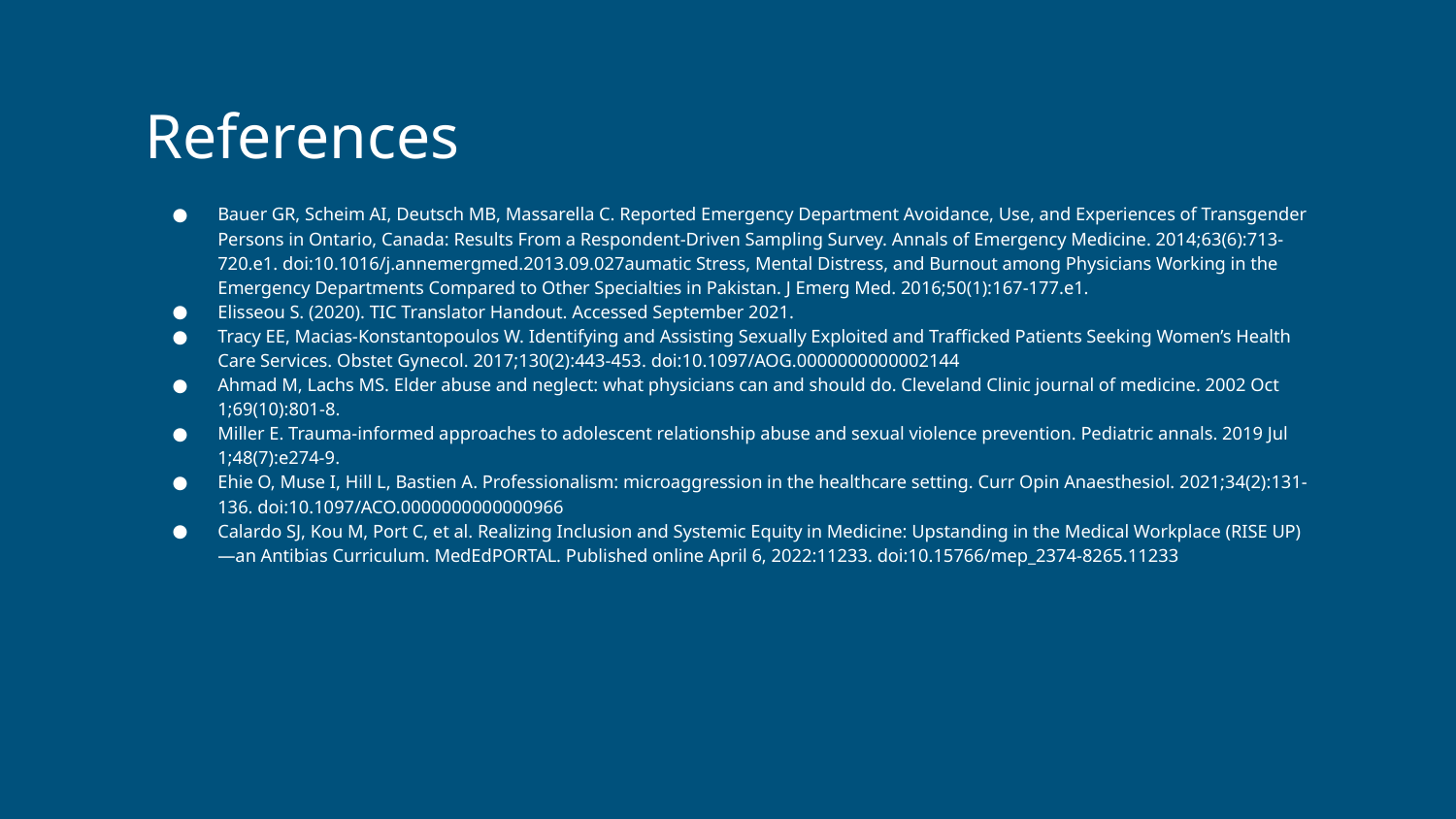

# References
Bauer GR, Scheim AI, Deutsch MB, Massarella C. Reported Emergency Department Avoidance, Use, and Experiences of Transgender Persons in Ontario, Canada: Results From a Respondent-Driven Sampling Survey. Annals of Emergency Medicine. 2014;63(6):713-720.e1. doi:10.1016/j.annemergmed.2013.09.027aumatic Stress, Mental Distress, and Burnout among Physicians Working in the Emergency Departments Compared to Other Specialties in Pakistan. J Emerg Med. 2016;50(1):167-177.e1.
Elisseou S. (2020). TIC Translator Handout. Accessed September 2021.
Tracy EE, Macias-Konstantopoulos W. Identifying and Assisting Sexually Exploited and Trafficked Patients Seeking Women’s Health Care Services. Obstet Gynecol. 2017;130(2):443-453. doi:10.1097/AOG.0000000000002144
Ahmad M, Lachs MS. Elder abuse and neglect: what physicians can and should do. Cleveland Clinic journal of medicine. 2002 Oct 1;69(10):801-8.
Miller E. Trauma-informed approaches to adolescent relationship abuse and sexual violence prevention. Pediatric annals. 2019 Jul 1;48(7):e274-9.
Ehie O, Muse I, Hill L, Bastien A. Professionalism: microaggression in the healthcare setting. Curr Opin Anaesthesiol. 2021;34(2):131-136. doi:10.1097/ACO.0000000000000966
Calardo SJ, Kou M, Port C, et al. Realizing Inclusion and Systemic Equity in Medicine: Upstanding in the Medical Workplace (RISE UP)—an Antibias Curriculum. MedEdPORTAL. Published online April 6, 2022:11233. doi:10.15766/mep_2374-8265.11233
