# Supplementary material for: Trauma-Informed Care for Acute Care Settings: A Novel Simulation Training for Medical Students
Source: MedEdPORTAL. 2023 Jul 28;19:11327. doi: 10.15766/mep_2374-8265.11327 (PMC10376910; doi:10.15766/mep_2374-8265.11327)
Supplement: Supplementary file 1 — TIC Acute Care Didactic.pptxSimulation Cases.docxDebriefing Materials.docxSimulation Checklists.docxSurvey Questions.docx [file mep_2374-8265.11327-s001.zip › C. Debriefing Materials.docx]

**Trauma-Informed Care in Acute Care Settings:**

**Debriefing Materials**

Following each simulation, students will gather back in the main auditorium or conference room to discuss how they felt the simulation went. Specific topics pertaining to each simulation should be discussed, ideally with interweaving discussion of these topics with student feedback on performance.

Not all of these have to be discussed, but generally most important topics to cover as debrief are listed first.

Case 1 (Intimate Partner Violence)

Discussion Questions

- How did that feel? What went well, what could have gone better?
- How did it feel to have to be stern with a partner?

TIC Clinical Pearls

- IPV situations: partnership with patient
  - Patient knows best, for example: whether it is safe for them to take information/pamphlets home with them or not
  - Assess the concern for other potential ADULT victims in the home: treating your patient may have implications for other potential victims
  - Social work can be helpful
  - Do not worry alone: call your attending/senior resident into the situation
- How does the situation change when the patient discloses that there are children in the home also exposed to violence by the partner?
  - Duty to report
  - Even though you have a duty to report, you can still be tactful by enlisting patient’s help for best way to do this
- De-escalation strategies when perpetrators become upset
  - Blaming hospital policy
  - Using laboratory tests or imaging as a technique to talk to the patient alone
  - Stating that it is your standard practice to ask patients questions alone without any family/friends present
- Last resort strategies
  - Having attending/senior resident step in and “put their foot down”
  - Calling security
  - Alerting administration
  - Very rare: ED placed on lockdown (always try to avoid these drastic steps, but sometimes necessary if there is significant safety risks)

Case 2 (Gender Affirming Surgery Post-Op Complications)

Discussion Questions

- How did that feel? What went well, what could have gone better?
- How did you feel seeing the patient distressed by the idea of having to have a physical exam, yet knowing you had to do it for their safety?
- How did you feel being caught in the situation where a senior team member kept misgendering the patient?
- How did you respond to this microaggression? How would you try to respond to this in the future?

TIC Clinical Pearls:

- TIC for sexual-and-gender minority patients can also involve knowing the particulars of hormone replacement therapy and gender affirming care: these procedures are meticulous, take hours, and complications are certainly possible. Clinically knowing the most common complications of gender affirming procedures can also help you take better care of patients .

Case 3 (IVDU & Medical Discrimination)

- How did that feel? What went well, what could have gone better?
- How did it feel when you found out the patient had originally “lied” to you about how they were injured in the first place?
- How did it feel to have a senior team member be disrespectful and use stigmatizing language in front of the patient?
  - How do you feel responding to this?
  - How might you respond to this in the future?
  - Many times individuals usually don’t mean to harm the patient, but nevertheless might let slip a stigmatizing word that can impact the care relationship
- What are some words you can think of to replace “addict, junkie, pain seeker” which both respect patients and also acknowledge the dependence on substance use?

TIC Clinical Pearls

- TIC for patients who self-inject drugs also means understanding the language, the equipment, etc. for IVDU. Being comfortable talking about injection logistics with patients helps you feel more confident in eliciting conversations about their drug use.
- Assessing for safe self-injection practices:
  - Type of needle used, where are you injecting, licking needles (Strep viridans, other oral flora anaerobes), skin popping, sharing needles (HIV, Hep C, Hep B)
  - Type of water used (tap, water bottle, sterile) [think pseudomonas and gram negatives]
  - Cotton filters: reuse? (Cotton fever)
  - Cookers: tetanus
  - Acids used to dissolve heroin/fentanyl (pure vitamin C vs. lemon juice (candida) vs. others
  - Tourniquets (belts→ possible limb ischemia)
  - Alcohol swabs, basic first-aid supplies for self-treating wounds, hand hygiene before injecting (Staph, GAS)
  - Buddy system: do you use alone or with friends?
    - Naloxone training/access?
  - Access to syringe exchange programs?
- Immediate symptom control:
  - Warm blankets
  - Food & water (entering diet orders)
  - Nicotine replacement
  - COWS and CIWA scoring
  - Pharmacologic treatment for withdrawal and anxiety (only if enough time, as this is more involved material that could be stand-alone lecture)
    - Clonidine for adrenergic symptoms
    - Methadone vs. Suboxone in-patient
  - Naloxone on discharge
