# Supplementary material for: Trauma-Informed Care for Acute Care Settings: A Novel Simulation Training for Medical Students
Source: MedEdPORTAL. 2023 Jul 28;19:11327. doi: 10.15766/mep_2374-8265.11327 (PMC10376910; doi:10.15766/mep_2374-8265.11327)
Supplement: Supplementary file 1 — TIC Acute Care Didactic.pptxSimulation Cases.docxDebriefing Materials.docxSimulation Checklists.docxSurvey Questions.docx [file mep_2374-8265.11327-s001.zip › D. Simulation Checklists.docx]

**Trauma-Informed Care in Acute Care Settings:**

**Simulation Checklists**

**Case 1: Intimate Partner Violence Case**

| **Introduction** | **Performed** | **Did Not Perform** |
| --- | --- | --- |
| Student introduces themselves and their role |  |  |
| Student asks permission for patient’s pronouns and preferred name |  |  |
| Student communicates with patient at same physical level |  |  |
| Student does not come between the door and the patient |  |  |
| **History Taking** |  |  |
| Student offers patient the choice to opt out of questions |  |  |
| Student provides anticipatory guidance by introducing questions and explaining why they are asking |  |  |
| Student asks about and does not assume the relationship between the two individuals in the room |  |  |
| Student responds to the potential for intimate partner violence by trying to address the patient alone (*For example: asking partner to step out of the room, making excuse to get imaging, etc.)* |  |  |
| Student asks screening questions for intimate partner violence (*For example: Do you feel safe in your relationship? Does your partner ever abuse you physically? Emotionally? Sexually?)* |  |  |
| Student asks screening questions for patient safety (*For example: Are you in immediate danger? Do you have somewhere safe to go?)* |  |  |
| **Physical Exam** |  |  |
| Student obtains verbal consent before physical touch |  |  |
| Student provides anticipatory guidance, introduces exam steps, and explains reasoning for exam maneuvers |  |  |
| Student remains within eyesight of the patient throughout encounter |  |  |

**Case 2: Gender-Affirming Surgery Post-Op Complications Case**

| **Introduction** | Performed | Did Not Perform |
| --- | --- | --- |
| Student introduces themselves and their role |  |  |
| Student asks permission for patient’s pronouns and preferred name |  |  |
| Student communicates with patient at same physical level |  |  |
| Student does not come between the door and the patient |  |  |
| **History Taking** |  |  |
| Student offers patient the choice to opt out of questions at any time |  |  |
| Student provides anticipatory guidance by introducing questions and explaining why they are asking |  |  |
| Student addresses the staff member who misgenders the patient by checking in with the patient |  |  |
| Student addresses the staff member who misgenders the patient by bringing it up to the staff member |  |  |
| **Physical Exam** |  |  |
| Student obtains verbal consent before physical touch |  |  |
| Student provides anticipatory guidance, introduces exam steps, and explains reasoning for exam maneuvers |  |  |
| Student uses patient-centered terms when discussing anatomy (*For example asking the* patient what terms they would like to use for their anatomy) |  |  |
| Student remains within eyesight of the patient throughout encounter |  |  |
| Student recognizes patient is distressed by exam and appropriately responds (For example: *C*hecks in, validates feelings, or suggests taking a break) |  |  |

**Case 3: Drug Use & Medical Distrust Case**

| **Introduction** | **Performed** | **Did Not Perform** |
| --- | --- | --- |
| Student introduces themselves and their role |  |  |
| Student communicates with patient at same physical level |  |  |
| Student does not come between the door and the patient |  |  |
| **History Taking** |  |  |
| Student offers patient the choice to opt out of questions at any time |  |  |
| Student provides anticipatory guidance by introducing questions and explaining why they are asking |  |  |
| Student addresses the staff member who uses stigmatizing language about the patient by checking in with the patient |  |  |
| Student addresses the staff member who uses stigmatizing language about patient by bringing it up to the staff member |  |  |
| Student uses person-centered language, avoiding stigmatizing words (i.e. “addict”) when discussing patient |  |  |
| Student asks a detailed and appropriate social history to understand the patient’s socioeconomic situation (*For example: Asking about housing, insurance, primary care usage, etc.*) |  |  |
| Student asks a detailed and appropriate history to understand the patient’s drug use (*For example: Last use, method of use, safety precautions including Naloxone and clean needles, etc.)* |  |  |
| **Physical Exam** |  |  |
| Student obtains verbal consent before physical touch |  |  |
| Student provides anticipatory guidance, introduces exam steps, and explains reasoning for exam maneuvers |  |  |
| Student remains within eyesight of the patient throughout encounter |  |  |
