# Supplementary material for: Trauma-Informed Care for Acute Care Settings: A Novel Simulation Training for Medical Students
Source: MedEdPORTAL. 2023 Jul 28;19:11327. doi: 10.15766/mep_2374-8265.11327 (PMC10376910; doi:10.15766/mep_2374-8265.11327)
Supplement: Supplementary file 1 — TIC Acute Care Didactic.pptxSimulation Cases.docxDebriefing Materials.docxSimulation Checklists.docxSurvey Questions.docx [file mep_2374-8265.11327-s001.zip › E. Survey Questions.docx]

**Trauma-Informed Care in Acute Care Settings:**

**Survey Questions**

**Pre-Event Survey:**

1. **The following will be used to de-identify your responses and match the pre & post surveys:** Please type the first 3 characters of your mother’s first name (please use ‘x’ if less than 2 characters) followed by the day of the month of your birth date (all lowercase letters). *Example: (Christine, January 1) = chr1*
2. **What year did you start medical school?**
   1. 2017
   2. 2018
   3. 2019
   4. 2020
   5. 2021
   6. Other:
3. **What phase of your training are you in?**
   1. Pre-Clinical
   2. Clinical Year
   3. Post-Clinical Year
4. **Have you had prior education (lectures, etc.) in medical school on how to obtain a history using trauma-informed care principles?**
   1. Yes
   2. No
5. **Have you had prior education (lectures, etc.) in medical school on how to perform a physical exam using trauma-informed care principles?**
   1. Yes
   2. No
6. **How often do you observe your preceptors (attendings, residents) purposely incorporate trauma-informed care principles in your prior clinical encounters?**
   1. Always
   2. Often
   3. Sometimes
   4. Rarely
   5. Never
7. **How often have you yourself purposely incorporated trauma-informed care principles in your prior clinical encounters?**
   1. Always
   2. Often
   3. Sometimes
   4. Rarely
   5. Never
8. **How important do you think trauma-informed care principles are to your future clinical encounters?**
   1. Not at all Important
   2. Slightly Important
   3. Somewhat Important
   4. Very Important
   5. Extremely Important
9. **How confident do you feel identifying situations where a trauma screening is indicated for patient safety and/or clinical care?**
   1. Not at all Confident
   2. Slightly Confident
   3. Somewhat Confident
   4. Very Confident
   5. Extremely Confident
10. **How confident do you feel sensitively inquiring patients about past adverse life experiences if relevant to their clinical care?**
    1. Not at all Confident
    2. Slightly Confident
    3. Somewhat Confident
    4. Very Confident
    5. Extremely Confident
11. **How confident do you feel actively responding to situations where other individuals may treat patients in manners violating trauma-informed care principles?**
    1. Not at all Confident
    2. Slightly Confident
    3. Somewhat Confident
    4. Very Confident
    5. Extremely Confident
12. **How familiar are you with using appropriate language for physical exams incorporating trauma-informed care principles?**
    1. Not at all Familiar
    2. Slightly Familiar
    3. Somewhat Familiar
    4. Very Familiar
    5. Extremely Familiar
13. **How familiar are you with performing physical exams incorporating trauma-informed care principles?**
    1. Not at all Familiar
    2. Slightly Familiar
    3. Somewhat Familiar
    4. Very Familiar
    5. Extremely Familiar
14. **In which scenario(s) should clinicians approach patients using trauma-informed care principles?**
    1. Patient presenting for medical care related to prior trauma
    2. Patient presenting for acute psychological care
    3. Patient presenting for longitudinal primary care
    4. All of the above
15. **In which of the following scenario(s) would you ask additional questions if a patient discloses trauma?**
    1. Patient with experiences of childhood adversity
    2. Patient with abusive partner
    3. Patient with history of sexual assault
    4. Patient with prior negative healthcare experiences
16. **Which of the following is NOT one of SAMHSA’s six key principles for trauma-informed approach?**
    1. Safety
    2. Peer Support
    3. Collaboration & Mutuality
    4. Empowerment, Voice, & Choice
    5. System Transparency
17. **Approximately how many individuals have experienced at least one Adverse Childhood Experience (ACE) in their life?**
    1. ~10%
    2. ~25%
    3. ~50%
    4. ~75%
    5. ~90%
18. **You are a physician evaluating a patient with substance use disorder, and the nurse continuously uses the word “addict” in front of the patient. The most appropriate immediate next step would be to:**
    1. Step out and file an administrative report
    2. Ask the nurse to step out, then address the concern with the patient and the nurse separately
    3. Address the nurse’s mistake in front of the patient
    4. Consult the clinical social worker to interview the patient with you
19. **How useful do you believe simulation-based exercises are for learning about trauma-informed care principles?**
    1. Not at all Useful
    2. Slightly Useful
    3. Somewhat Useful
    4. Very Useful
    5. Extremely Useful
20. **How useful do you believe simulation-based exercises are for preparing you to interact with patients using trauma-informed care principles?**
    1. Not at all Useful
    2. Slightly Useful
    3. Somewhat Useful
    4. Very Useful
    5. Extremely Useful
21. **How much do you agree with the following statement: “More simulation-based exercises should be incorporated into the existing medical school curriculum”?**
    1. Not at all
    2. Slightly
    3. Somewhat
    4. Very Much
    5. Extremely
22. **What are you hoping to learn from this session on Trauma-Informed Care?**
23. **What are some challenges to learning about or practicing “Trauma-Informed Care” in medical school?**

**Post-Training Survey:**

1. **The following will be used to de-identify your responses and match the pre & post surveys:** Please type the first 3 characters of your mother’s first name (please use ‘x’ if less than 2 characters) followed by the day of the month of your birth date (all lowercase letters). *Example: (Christine, January 1) = chr1*
2. **How important do you think trauma-informed care principles are to your future clinical encounters?**
   1. Not at all Important
   2. Slightly Important
   3. Somewhat Important
   4. Very Important
   5. Extremely Important
3. **How confident do you feel identifying situations where a trauma screening is indicated for patient safety and/or clinical care?**
   1. Not at all Confident
   2. Slightly Confident
   3. Somewhat Confident
   4. Very Confident
   5. Extremely Confident
4. **How confident do you feel sensitively inquiring patients about past adverse life experiences if relevant to their clinical care?**
   1. Not at all Confident
   2. Slightly Confident
   3. Somewhat Confident
   4. Very Confident
   5. Extremely Confident
5. **How confident do you feel actively responding to situations where other individuals may treat patients in manners violating trauma-informed care principles?**
   1. Not at all Confident
   2. Slightly Confident
   3. Somewhat Confident
   4. Very Confident
   5. Extremely Confident
6. **How familiar are you with using appropriate language for physical exams incorporating trauma-informed care principles?**
   1. Not at all Familiar
   2. Slightly Familiar
   3. Somewhat Familiar
   4. Very Familiar
   5. Extremely Familiar
7. **How familiar are you with performing physical exams incorporating trauma-informed care principles?**
   1. Not at all Familiar
   2. Slightly Familiar
   3. Somewhat Familiar
   4. Very Familiar
   5. Extremely Familiar
8. **How likely are you to purposely incorporate “Trauma- Informed Care” principles in your future clinical encounters?**
   1. Definitely
   2. Very Likely
   3. Likely
   4. Somewhat Likely
   5. Not Likely
9. **How effective was this session at meeting the stated learning objectives?**
   1. Extremely Effective
   2. Somewhat Effective
   3. Neutral
   4. Somewhat Ineffective
   5. Ineffective
10. **In which scenario(s) should clinicians approach patients using TIC principles?**
    1. Patient presenting for medical care related to prior trauma
    2. Patient presenting for acute psychological care
    3. Patient presenting for longitudinal primary care
    4. All of the above
11. **In which of the following scenario(s) would you ask additional questions if a patient discloses trauma?**
    1. Patient with experiences of childhood adversity
    2. Patient with abusive partner
    3. Patient with history of sexual assault
    4. Patient with prior negative healthcare experiences
12. **Which of the following is NOT one of SAMHSA’s six key principles for trauma-informed approach?**
    1. Safety
    2. Peer Support
    3. Collaboration & Mutuality
    4. Empowerment, Voice, & Choice
    5. System Transparency
13. **Approximately how many individuals have experienced at least one Adverse Childhood Experience (ACE) in their life?**
    1. ~10%
    2. ~25%
    3. ~50%
    4. ~75%
    5. ~90%
14. **You are a physician evaluating a patient with substance use disorder, and the nurse continuously uses the word “addict” in front of the patient. The most appropriate immediate next step would be to:**
    1. Step out and file an administrative report
    2. Ask the nurse to step out, and address the concern with the patient and the nurse separately
    3. Address the nurse’s mistake in front of the patient
    4. Consult the clinical social worker to interview the patient with you
15. **How useful do you believe simulation-based exercises are for learning about trauma-informed care principles?**
    1. Not at all Useful
    2. Slightly Useful
    3. Somewhat Useful
    4. Very Useful
    5. Extremely Useful
16. **How useful do you believe simulation-based exercises are for preparing you to interact with patients using trauma-informed care principles?**
    1. Not at all Useful
    2. Slightly Useful
    3. Somewhat Useful
    4. Very Useful
    5. Extremely Useful
17. **How much do you agree with the following statement: “More simulation-based exercises should be incorporated into the existing medical school curriculum”?**
    1. Not at all
    2. Slightly
    3. Somewhat
    4. Very Much
    5. Extremely
18. **What did you find the most useful from participating in this session?**
19. **What would you change about the session going forward?**
20. **How do you plan to incorporate what you learned to your future clinical encounters?**

***Thank you for participating in our survey!***
