# Supplementary material for: Trauma-Informed Care for Acute Care Settings: A Novel Simulation Training for Medical Students
Source: MedEdPORTAL. 2023 Jul 28;19:11327. doi: 10.15766/mep_2374-8265.11327 (PMC10376910; doi:10.15766/mep_2374-8265.11327)
Supplement: Supplementary file 1 — TIC Acute Care Didactic.pptxSimulation Cases.docxDebriefing Materials.docxSimulation Checklists.docxSurvey Questions.docx [file mep_2374-8265.11327-s001.zip › Educational Summary Report_11327.pdf]

MedEdPORTAL® The AAMC Journal of Teaching and Learning Resources

Original Publication Open Access

Trauma-Informed Care for Acute Care Settings: A Novel Simulation Training for Medical Students

Caroline H. Lee*, Carlos Dos Santos, Taylor Brown, MD, Henry Ashworth, MD, MPH, Jason J. Lewis, MD

*Corresponding author: Caroline.Lee@ucsf.edu

Abstract

Introduction: Physicians often care for patients who have experienced traumatic events including abuse, discrimination, and violence. Trauma-informed care (TIC) is a framework that recognizes the prevalence of trauma, promotes patient empowerment, and minimizes retraumatization. There are limited education curricula on how to apply TIC to acute care settings, with simulation-based training presenting a novel educational tool for this aim. **Methods:** Students participated in a didactic on TIC principles and its applications in acute care settings. Learners participated in three simulation cases where they performed physical exams and gathered history on patients with urgent medical needs related to intimate partner violence, transgender health, and health care discrimination. Debriefing followed each simulation. **Results:** Seventeen medical students participated across four sessions. The sessions were evaluated with pre- and postparticipation surveys, including Likert scales and free-response questions. After participation, individuals' self-assessed confidence improved across multiple domains, including identifying situations for trauma screenings, inquiring about trauma, and responding as a bystander. Learners also felt more familiar with TIC-specific history taking and physical exam skills. Finally, simulation was perceived as a beneficial educational tool. All findings were statistically significant ($p \leq .01$). **Discussion:** Our simulation-based training enabled students to practice conversations and interventions related to trauma. This novel training represents a feasible and effective means for teaching TIC for acute care settings, including in the emergency department and in-patient settings. Development and evaluation were supported by the Society for Academic Emergency Medicine.

Keywords:
Trauma-Informed Care, Communication Skills, Emergency Medicine, Gender Issues in Medicine, Intimate Partner Violence, Pediatric Emergency Medicine, Simulation, Diversity, Equity, Inclusion

Educational Objectives

By the end of this session, learners will be able to:

1. Identify the importance of incorporating trauma-informed care (TIC) into clinical practice as a universal precaution in the acute care setting.
2. Identify clinical situations in which performing safety screenings for trauma history is indicated in the acute care setting.
3. Improve comfort in using TIC principles during a physical exam in an acute care setting.
4. Increase confidence when responding to colleagues through bystander intervention using TIC principles.

Citation:
Lee CH, Dos Santos C, Brown T, Ashworth H, Lewis JJ. Trauma-informed care for acute care settings: a novel simulation training for medical students. *MedEdPORTAL*. 2023;19:11327.
https://doi.org/10.15766/mep_2374-8265.11327

Introduction

Physicians often care for patients who have experienced trauma, which is defined as experiences or events that have lasting adverse effects on one's mental, physical, social, or spiritual well-being.[1,2] Patients who have experienced trauma or adverse experiences have higher rates of mental health conditions and chronic physical symptoms.[3,4] Further retraumatization may occur during medical encounters, leading to distrust and decreased usage of essential services, including preventative screenings.[3,4] Avoidance of preventative health care may in turn also lead to increased utilization of the emergency department (ED).[3-6]

Trauma-informed care (TIC) is a framework that describes how providers can reduce retraumatization and promote healing for their patients.[2] This approach is based on the following six key

Copyright © 2023 Kung et al. This is an open-access publication distributed under the terms of the Creative Commons Attribution license.

principles: (1) safety; (2) trustworthiness and transparency; (3) peer support; (4) collaboration and mutuality; (5) empowerment, voice, and choice; and (6) cultural, historical, and gender Issues.[2] Integrating TIC into clinical care has led to increased perceived partnership between patients and physicians, which may improve patient engagement and health outcomes.[7,8]

Acute care settings, including the ED and urgent care, treat millions of patients per year who have had exposure to trauma, including experiences of abuse, discrimination, and violence.[2,9] These settings also have high volume and turnover, with providers caring for patients experiencing high levels of stress and/or acute pain. An understanding of TIC is essential when practicing in these settings, as patients may present with signs, symptoms, and/or sequelae of trauma (e.g., intimate partner violence [IPV], elder or child abuse, or community violence). Additionally, adults who frequently present to the ED have high rates of post-traumatic stress disorder and adverse childhood experiences (ACEs), suggesting this patient population has greater prevalence of preexisting adversity.[9-11] The application of TIC may also improve aspects of patient-centered care and safety, such as by reducing restraint usage in the ED.[12-15]

Despite the significant need for the implementation of TIC in acute care settings, several barriers for acute care providers exist, including a lack of longitudinal relationships with patients, limited training, high acuity, and time constraints.[16,17] Due to the lack of continuity of care, clinicians must also be specifically trained on situations when performing a trauma-informed safety screening is warranted for patient safety.

Educational trainings for health care workers can improve their understanding of TIC and implementation of TIC principles.[13,17] In particular, incorporating these trainings during undergraduate and graduate medical education can provide developing physicians with an important framework to apply to their future patient encounters.[18] Undergraduate medical education in TIC, as described in *MedEdPORTAL,* has utilized tools including small-group discussions, peer role-play, and standardized patient demonstrations.[18-22]

Our educational workshop focuses on integrating TIC into acute care settings using simulations, which are designed to reproduce complex clinical encounters in a controlled and safe environment.[23,24] Simulations are particularly helpful for recreating high-risk situations with sensitive topics and vulnerable patient populations, thus making them particularly well suited for educating learners about TIC.[23,24]

This workshop describes a novel application to TIC to educate medical students on how to apply their skills to the acute care setting through simulation-based training. Simulations of trauma-informed approaches have previously been developed on specific topics such as applications to human trafficking and approaching ACEs in the pediatric population.[21,25,26] To our knowledge, this publication is the first of its kind to describe a simulation training to educate medical students on TIC practices in acute care settings.

Methods

Development

We developed a simulation workshop on TIC practices in acute care settings building off previous literature on TIC workshops.[17-20] We recruited multidisciplinary TIC experts, including physicians, researchers, and nurses, from Brigham and Women's Hospital, Harvard Medical School, and Beth Israel Deaconess Medical Center to review the content of the didactic presentation and simulation cases. Cases were based on real-life patient cases experienced in the ED using input from emergency medicine residents and attendings, then reviewed by the experts mentioned above for content accuracy and patient representation. The workshop was part of a larger educational movement at Harvard to longitudinally incorporate TIC into undergraduate medical education.[27,28]

Two authors (Caroline H. Lee and Carlos Dos Santos) had participated as learners and one author (Taylor Brown) helped develop the TIC curriculum for first-year medical students at Harvard Medical School that focusing on applying TIC history taking and physical exam skills.[18,20] Based on these experiences, we identified gaps in the curriculum and developed our educational materials to focus on knowledge not covered in the existing curriculum, such as bystander training and trauma-informed safety screening in acute care settings.

The session consisted of a didactic lecture and three simulation cases with patients who had urgent medical needs and a relevant history of traumatic experiences. Facilitators first presented a didactic that covered an introduction to TIC, unique challenges of delivering TIC in acute care settings, and applications to history taking and physical exam procedures (Appendix A). The didactic included opportunities for active learning through prompts for reflection and open-ended discussion. Learners then interacted with three cases: (1) a patient presenting with physical sequelae from IPV, (2) a transgender patient with postoperative complications following

Copyright © 2023 Kung et al. This is an open-access publication distributed under the terms of the Creative Commons Attribution license.

a gender-affirming surgical procedure, and (3) a patient with IV drug use and history of stigmatizing experiences by medical providers.

In accordance with trauma-informed medical education, we incorporated TIC principles into the delivery of the workshop to ensure that students were supported while engaging with challenging subject matter.[28] At the beginning of the didactic and the start of simulation cases, facilitators provided content warnings about sensitive topics, reminders to practice self-care, and invitations to take breaks from the workshop if needed. We also provided institution-specific self-care resources. Finally, the facilitators acted as the patients, so students did not have to embody the lived experiences of trauma. Instead, students interacted with the simulated patients in patient care roles similar to those in which they would encounter such individuals in real clinical settings.

The simulation sessions were performed in the Shapiro Simulation and Skills Center at Beth Israel Deaconess Medical Center in a simulated ED. We recruited first- through fourth-year medical students from Harvard Medical School and the University of Massachusetts Chan Medical School via email invitation.

We provided students with a $10 gift card as compensation for their participation. Implementation of the sessions was supported by a grant from the Society for Academic Emergency Medicine.

Equipment

Equipment used to implement the three simulation cases included the following:

- High-fidelity mannequin (with vaginal external genitalia)
- Hospital bed
- Fake blood to simulate hemorrhage
- IV, IV pole, normal saline bag and tubing, syringes, and needles
- Blood pressure cuff
- Screen to display vital signs (heart rate, blood pressure, respiratory rate), with sound and alarms on

Personnel

Facilitators (upper-year medical students and emergency medicine residents) and faculty mentors were chosen due to their preexisting familiarity with TIC. One faculty mentor had content expertise in emergency medicine and medical student education, and another faculty mentor had content expertise in TIC. All facilitators received the didactic, case templates, and debriefing materials (Appendices A-C) beforehand to review in preparation.

At least two facilitators (upper-year medical student or resident) played the following roles:

- Case 1: IPV
  - Simulated participant: patient
  - Simulated participant: patient's partner
- Case 2: transgender patient
  - Simulated participant: patient
  - Simulated participant: health care colleague (nurse, medical assistant)
- Case 3: health care discrimination
  - Simulated participant: patient
  - Simulated participant: resident or attending

Faculty mentors provided feedback for the learners during the debriefing session. A simulation technician was responsible for changing vital signs and setting up the simulation environment.

Implementation

We hosted four sessions, with three to five learners per session. Three of these sessions were optional learning activities open to all medical students, and one session was integrated into an Emergency Medicine Bootcamp course for graduating medical students.

Each session lasted approximately 2.5 hours. The workshop began with a 1-hour interactive didactic followed by three simulation cases. Each case took 30 minutes including the simulation and debrief.

Prior to each simulation, participants self-designated themselves to at least one of the following roles: team leader, history taker, physical examiner, communicator with other health care staff, and oral presenter. Roles rotated with each simulation.

Before each case, a facilitator established the setting as the ED, introduced the one-liner about the patient, and asked the team to gather a history and perform an exam on the patient. Participants were not alerted to the specific type of adversity the patient had experienced. When participants entered the room, vital signs were already provided to the team via simulation monitors. A summary of the case scenarios appears below and is described further in the case templates (Appendix B).

Case 1: The patient was introduced as a male presenting with unilateral arm pain with a history of prior fractures. Learners encountered the patient and his partner in the examining room. When individuals asked the patient questions, his partner periodically answered for the patient and interjected into the conversation. The history provided to explain the

Copyright © 2023 Kung et al. This is an open-access publication distributed under the terms of the Creative Commons Attribution license.

injury was vague and included changing details. If participants separated the partner and patient to speak to the patient alone, the patient revealed a history of IPV and fear regarding his safety at home. This situation provided participants with the opportunity to practice removing potential suspects for confidential conversations and to identify that a trauma-informed safety screen focused on IPV was indicated.

Case 2: The patient was introduced as a female presenting with light-headedness. Participants encountered a mannequin (voiced by a facilitator) that was hypotensive and tachycardic. She revealed that she had postsurgical bleeding after a vaginoplasty. During the case, another health care worker misgendered the patient. This situation offered learners the opportunity to address potential conflict in a health care team, to practice conversations related to transgender health, and to perform a trauma-informed physical exam.

Case 3: The patient was introduced as a male with right arm pain. While he originally explained his pain as caused by an animal bite, he started to exhibit signs of opioid withdrawal, including nausea and restlessness, and later revealed that he used IV drugs. During the assessment, the facilitator, acting as a supervising physician, made statements referring to a history of frequent ED visits and implying that the patient was malingering, which the patient overheard. This situation gave participants the chance to practice establishing trust and rapport with patients who may have distrust related to prior experiences in the health care system and to manage comments from other health care providers.

During each case, the facilitator completed a skills checklist of TIC actions students implemented during their SIM (Appendix D). At the conclusion of each scenario, students provided an oral summary of patient history, assessment, and plan to the facilitator.

Assessment

The facilitator completed the simulation checklists (Appendix D) to record TIC-relevant actions that students implemented during each scenario. Facilitators used the checklists to guide feedback provided during each debriefing session. We created the checklists to assess completion of principles from prior TIC curricula on history taking and physical exam skills, as well as tasks specific to each case.[18,20]

We distributed pre- and posttraining survey questionnaires (Appendix E) to participants. The questionnaires used Likert-scale, multiple-choice, and free-response questions. Each learner answered questions that evaluated self-perceived confidence and familiarity with TIC skills using a 5-point Likert scale (1 = *not at all confident/familiar,* 5 = *extremely confident/familiar*), utility of simulations to teach TIC using a 5-point Likert scale (1 = *not at all useful,* 5 = *extremely useful*), multiple-choice knowledge questions, and free-response questions about major takeaways and suggestions for improvement. We designed these evaluations to assess reaction and learning in the Kirkpatrick model.[29] We developed questions based on a literature review of evaluations for prior simulation and TIC curricula.[18,20-22,26] We then tailored questions to our specific educational objectives and solicited feedback from content experts in both medical education and TIC.

We analyzed the survey results using Microsoft Excel. A Fisher exact test was used to compare pre- and postparticipation answers. Free responses were analyzed for recurrent themes.

Debriefing

Given the difficult interactions and sensitive topics simulated in the sessions, a dedicated debriefing session after each case was facilitated to allow for participant reflection.

Following each case, students met with facilitators to discuss reactions to the case in a roundtable and guided-discussion format. Students were first asked to self-assess their performance by discussing what they perceived had gone well and identifying areas for improvement. Facilitators then provided feedback on the students' interactions with the patient and other individuals in the simulation and suggested areas of improvement based on the TIC skills checklist (Appendix D).

Finally, facilitators summarized the following teaching points relevant to each case, as listed in the debriefing materials (Appendix C):

- Case 1: methods of talking privately to patients suspected of being victims of violence, de-escalation strategies, and next steps for ensuring patient safety
- Case 2: balancing the need for acute medical management and TIC principles, TIC applications to invasive physical exams, and bystander responses to misgendering
- Case 3: discrimination in the health care system, safety for individuals who use injection drugs, and engagement of other team members using stigmatizing language

Results

Seventeen medical students participated in the training, all of whom completed the pre- and postsimulation surveys. Of all the learners, 65% had not yet completed their core clinical year and

Copyright © 2023 Kung et al. This is an open-access publication distributed under the terms of the Creative Commons Attribution license.

35% had. Most participants had had some form of prior education in medical school on obtaining a history (88%) and performing a physical exam (76%) using TIC principles.

Students reacted unanimously positively to the session, with 100% rating it as somewhat or extremely effective in meeting the stated learning objectives.

Students' self-assessed confidence in relevant TIC knowledge improved after participation in the workshop. They reported improved confidence in identifying situations where a trauma-informed safety screening was indicated in acute care settings ($p \leq .01$), sensitively inquiring about trauma ($p \leq .01$), and responding as a bystander after observing breaches in TIC ($p \leq .01$; Table 1). Learners also felt more familiar with specific skills, including TIC-informed history taking ($p \leq .01$) and performing TIC-informed physical exams ($p \leq .01$; Table 1).

The utility of simulation-based exercises as a mechanism for learning about TIC and preparing for patient interactions was perceived as more useful after participation in the workshop ($p \leq .01$; Table 2). Students also expressed greater interest in having more simulations integrated into their medical school curriculum after their experience ($p \leq .01$; Table 2).

The differences in learners' performance on knowledge questions were variable (Table 3). After participation, there were improvements in the number of correct responses to questions where students were asked to determine whether all scenarios were applicable to TIC and to identify situations in which to elicit further information after a patient's disclosure of trauma. There was no change in performance on questions about the Substance Abuse and Mental Health Services Administration's six TIC principles and what percentage of individuals had experienced at least one ACE in their life. There was a slight decrease in the number of correct answers on a scenario-based question on the best course of action as a bystander.

Themes from the students' free-response questions are summarized in Table 4. Students expressed the goals of their participation in the workshop as being a desire to practice TIC skills, respond appropriately to patient disclosures of trauma, and understand TIC physical exam techniques. Challenges in medical school in learning about TIC included a lack of familiarity with the topic by their clinical preceptors, limited integration into the core curriculum, and difficulties in universally applying the principles. After the session, learners found that the opportunity to practice TIC in a supportive setting and the debriefing sessions were the most useful components of the workshop. Participants suggested areas of improvement such as having increased case difficulty for students of different clinical skill levels and a lower learner-to-facilitator ratio. Furthermore, major takeaways from the session included knowledge of specific TIC terminology to use with patients and, importantly, a desire to universally provide TIC to all their patients.

Discussion

Our educational intervention represents the first simulation-based one focused on applications of TIC to acute care settings. Prior

Table 1. Confidence and Familiarity With Trauma-Informed Care Before and After Participation ($N = 17$)

Question[a]	*M* (*SD*)	*Mdn*	Range	*p*
How confident do you feel identifying situations where a trauma screening is indicated for patient safety and/or clinical care?				
Presession	2.2 (0.9)	2	1-4	<.001
Postsession	3.5 (0.7)	4	2-5	
How confident do you feel sensitively inquiring patients about past adverse life experiences if relevant to their clinical care?				
Presession	2.2 (0.7)	2	1-3	.01
Postsession	3.2 (0.9)	3	1-5	
How confident do you feel actively responding to situations where other individuals may treat patients in manners violating trauma-informed care principles?				
Presession	1.8 (0.9)	2	1-4	.002
Postsession	2.8 (0.8)	3	1-4	
How familiar are you with using appropriate language for physical exams incorporating trauma-informed care principles?				
Presession	2.2 (0.9)	2	1-4	<.001
Postsession	3.4 (0.6)	3	2-4	
How familiar are you with performing physical exams incorporating trauma-informed care principles?				
Presession	2.2 (1.0)	2	1-4	.003
Postsession	3.2 (0.8)	3	2-5	

[a]Rated on a 5-point scale (1 = *not at all confident/familiar*, 2 = *slightly confident/familiar*, 3 = *somewhat confident/familiar*, 4 = *very confident/familiar*, 5 = *extremely confident/familiar*).

Copyright © 2023 Kung et al. This is an open-access publication distributed under the terms of the Creative Commons Attribution license.

Table 2. Utility of Simulations in Learning Trauma-Informed Care (*N* = 17)

Question[a]	*M* (*SD*)	*Mdn*	Range	*p*
How useful do you believe simulation-based exercises are for learning about trauma-informed care principles?				
Presession	3.8 (0.9)	4	2-5	.008
Postsession	4.5 (0.5)	5	4-5	
How useful do you believe simulation-based exercises are for preparing you to interact with patients using trauma-informed care principles?				
Presession	3.5 (0.9)	2	2-5	<.001
Postsession	4.5 (0.5)	4	4-5	
How much do you agree with the following statement: "More simulation-based exercises should be incorporated into the existing medical school curriculum"?				
Presession	4.0 (0.8)	4	2-5	<.001
Postsession	4.8 (0.4)	5	4-5	

[a]Rated on a 5-point scale (1 = *not at all useful/agree*, 2 = *slightly useful/agree*, 3 = *somewhat useful/agree*, 4 = *very useful/agree*, 5 = *extremely useful/agree*).

research has demonstrated the value of using simulations to practice TIC.[25,26] However, those situations were limited in scope and did not reflect the unique challenges present in acute care environments. It is essential to teach medical students how to integrate TIC principles into acute care settings, given the need to treat a patient population directly impacted by sequelae of trauma, to intervene to ensure patient safety, and to navigate unique challenges including time constraints and lack of privacy. Our novel simulation training offers students an opportunity to practice integrating TIC principles into acute care situations, preparing them to provide holistic care for a diversity of patients.

The workshop's measured outcomes demonstrated that simulating acute care settings to teach TIC was feasible, well received, and effective. Students' confidence and familiarity in specific TIC skills, from inquiring about adverse life experiences to actively responding as a bystander, significantly improved after their participation. Simulations were perceived as a valuable modality for education on TIC, and students expressed the desire for further simulation training during medical school. One of the 17 participants indicated feeling "not at all comfortable" with some TIC skills after the session, illustrating the need for this workshop to be part of ongoing longitudinally integrated TIC curricula. Overall, the workshop was successful in facilitating practice of TIC clinical skills, including history taking and performing physical exams.

There were limited improvements in the knowledge assessment questions, which may represent the fact that the answers were not explicitly highlighted in the training and that inherent ambiguity exists in answers to scenario-based questions. However, after participating in the session, all students were able to correctly identify TIC as an important universal precaution in all clinical scenarios. Additionally, this exercise was performed among students with high levels of exposure to TIC even before participation, due to the incorporation of TIC into the first-year curriculum at Harvard Medical School.[18] Greater knowledge gains may be observed in groups with less extensive backgrounds in TIC.

Table 3. Results of Knowledge-Based Assessment Questions (*N* = 17)

Question	Correct Answer	% Correct Answers Presession	% Correct Answers Postsession
In which scenario(s) should clinicians approach patients using trauma-informed care principles?	All of the above (patient presenting for medical care related to prior trauma; patient presenting for acute psychological care; patient presenting for longitudinal primary care)	88	100
In which of the following scenario(s) would you ask additional questions if a patient discloses trauma?	Patient with abusive partner	65	82
Which of the following is not one of SAMHSA's six key principles for trauma-informed approach?	System transparency	47	47
Approximately how many individuals have experienced at least one adverse childhood experience in their life?	~50%	53	53
You are a physician evaluating a patient with substance use disorder, and the nurse continuously uses the word addict in front of the patient. The most appropriate immediate next step would be to:	Ask the nurse to step out, then address the concern with the patient and the nurse separately	100	82

Abbreviation: SAMHSA, Substance Abuse and Mental Health Services Administration.

Copyright © 2023 Kung et al. This is an open-access publication distributed under the terms of the Creative Commons Attribution license.

Table 4. Themes From Free-Response Questions ($N = 17$)

Question	Themes	Representative Quotes
What are you hoping to learn from this session on TIC?	Practicing skills relevant to TIC	"I'm hoping to get practice and feedback at implementing TIC principles."
	Formulating responses to disclosures of trauma	"Learn how to appropriately respond to positive disclosures.""I'm hoping to learn what to do in situations where someone discloses trauma and what to say."
	Understanding of TIC physical exam techniques	"How to incorporate TIC into physical exam."
What are some challenges to learning about or practicing TIC in medical school?	Clinical instructors often lack familiarity with TIC practices	"Our preceptors haven't themselves had formal training in TIC and thus are not all well prepared to model or teach techniques/skills.""Preceptors lack of familiarity with TIC—therefore, it's hard to learn about it since it's not being modeled."
	Limited integration of TIC into medical school curriculum	"It's not incorporated into the core of the curriculum."
	Difficulties adjusting TIC to each patient	"Trauma is so ubiquitous it seems difficult to fully teach how to practice medicine in a trauma-informed way, knowing that trauma is such a difficult subject and looks so different for everyone."
What did you find the most useful from participating in this session?	Opportunity to apply skills during the simulations	"Getting to practice asking the difficult questions and responding to disclosures of trauma.""Thrown into the real stuff but no consequences of messing up.""The didactic session followed by the simulation, it mixed theory with action."
	Debrief sessions helped to integrate principles	"The reflections and discussions after each simulation were the most helpful."
What would you change about the session going forward?	Case variations for medical students in early versus later years	"Beneficial for an advanced session to include more difficult cases (more difficult trauma, more pushback from patient).""I wish I had more interview/physical exam skills going in, but it was still very helpful."
	Decreased number of participants per simulation	"More simulation stations in pairs. Four people causes some diffusion of responsibility."
How do you plan to incorporate what you learned to your future clinical encounters?	Using specific TIC language	"I plan to first practice using TIC language during my clinical encounters!"
	Implementing a universal approach to all patients	"I hope to use TIC with all my patients and do my best to help patients feel as comfortable as possible."

Abbreviation: TIC, trauma-informed care.

There are several limitations to our curricular evaluation. First, most of our participants had some prior exposure to TIC during their medical school curriculum. Thus, our findings can be largely applied to students with a baseline understanding of TIC, and it is unknown if the intervention would have the same effect in populations with more limited prior exposure. The sample size was too small to compare differences between individuals who participated before and after their core clinical experiences. Additionally, because participants volunteered their time to participate, they already demonstrated interest in the topic of TIC and may have been comfortable with role-playing in the training. Next, postparticipation surveys were completed immediately after students finished the simulations. This methodology could not evaluate the effectiveness of this intervention after longer follow-up to see if the intervention led to long-term knowledge retention or increased application of TIC principles in clinical settings.

Some logistical challenges we encountered included the time and resources required to implement this training. While all our facilitators and faculty mentors had preexisting education on TIC practices, additional faculty development on TIC may need to be created and evaluated if knowledgeable facilitators are not readily available. Options to reduce the training from 2.5 hours due to limited time constraints include providing the didactic online asynchronously and/or selecting one or two simulation cases to implement instead of three. Participants provided feedback that the session would have been improved if there were even smaller groups for the simulations. Therefore, this intervention may be difficult to incorporate with fewer resources or larger groups. Despite these limitations, our simulation still provides a unique method to educate students on how to integrate TIC into clinical acute care environments.

The medical students who participated in our sessions noted that a key challenge to practicing TIC was that their preceptors often lacked familiarity with TIC practices. Therefore, future simulations teaching TIC should focus on utility in educating both residents and attending physicians who serve as clinicians and educational role models for trainees. Additionally, further research is needed to understand if simulation-based TIC workshops lead to retained knowledge and subsequent integration of learned skills into clinical care. These aims can be achieved by means of longitudinal evaluations that incorporate follow-up with

Copyright © 2023 Kung et al. This is an open-access publication distributed under the terms of the Creative Commons Attribution license.

students or clinical skills assessments through individual OSCE performances.

Overall, this novel training represents a well-received and effective means for teaching TIC skills for acute care environments.

Appendices

A. TIC Acute Care Didactic.pptx

B. Simulation Cases.docx

C. Debriefing Materials.docx

D. Simulation Checklists.docx

E. Survey Questions.docx

All appendices are peer reviewed as integral parts of the Original Publication.

Caroline H. Lee: Fourth-Year Medical Student, Harvard Medical School; ORCID: https://orcid.org/0000-0002-0750-9868

Carlos Dos Santos: Fourth-Year Medical Student, Harvard Medical School

Taylor Brown, MD: Second-Year Resident, Department of Emergency Medicine, Beth Israel Deaconess Medical Center

Henry Ashworth, MD, MPH: First-Year Resident, Department of Emergency Medicine, Highland Hospital, Alameda Health System

Jason J. Lewis, MD: Assistant Professor, Department of Emergency Medicine, Beth Israel Deaconess Medical Center

Disclosures:
None to report.

Funding/Support:
This project has been supported by a grant from the Society for Academic Emergency Medicine.

Prior Presentations:
Lee CH, Dos Santos C, Brown T, Ashworth H, Lewis JJ. Medical simulation training on trauma-informed care in the emergency department. Poster presented at: Council of Residency Directors in Emergency Medicine Academic Assembly; March 27, 2022; San Diego, CA.

Ethical Approval:
The Harvard Medical School Institutional Review Board deemed further review of this project not necessary.

Disclaimer:
Development and evaluation were supported by the Society for Academic Emergency Medicine.

References

1. Raja S, Hasnain M, Hoersch M, Gove-Yin S, Rajagopalan C. Trauma informed care in medicine: current knowledge and future research directions. *Fam Community Health*. 2015;38(3): 216-226. https://doi.org/10.1097/FCH.0000000000000071
2. Substance Abuse and Mental Health Services Administration. *SAMHSA's Concept of Trauma and Guidance for a Trauma-Informed Approach*. Substance Abuse and Mental Health Services Administration; 2014:1-20. HHS publication (SMA) 14-4884. Accessed May 15, 2023. https://ncsacw.acf.hhs.gov/userfiles/files/SAMHSA_Trauma.pdf
3. Bauer GR, Scheim AI, Deutsch MB, Massarella C. Reported emergency department avoidance, use, and experiences of transgender persons in Ontario, Canada: results from a respondent-driven sampling survey. *Ann Emerg Med*. 2014;63(6): 713-720.e1. https://doi.org/10.1016/j.annemergmed.2013.09.027
4. Purkey E, Davison C, MacKenzie M, et al. Experience of emergency department use among persons with a history of adverse childhood experiences. *BMC Health Serv Res*. 2020;20:455. https://doi.org/10.1186/s12913-020-05291-6
5. Rosenberg HJ, Rosenberg SD, Wolford GL II, Manganiello PD, Brunette MF, Boynton RA. The relationship between trauma, PTSD, and medical utilization in three high risk medical populations. *Int J Psychiatry Med*. 2000;30(3):247-259. https://doi.org/10.2190/J8M8-YDTE-46CB-GYDK
6. Farley M, Patsalides BM. Physical symptoms, posttraumatic stress disorder, and healthcare utilization of women with and without childhood physical and sexual abuse. *Psychol Rep*. 2001;89(3):595-606. https://doi.org/10.2466/pr0.2001.89.3.595
7. Green BL, Saunders PA, Power E, et al. Trauma-informed medical care: patient response to a primary care provider communication training. *J Loss Trauma*. 2016;21(2):147-159. https://doi.org/10.1080/15325024.2015.1084854
8. Menschner C, Maul A. *Key Ingredients for Successful Trauma-Informed Care Implementation*. Center for Health Care Strategies/Robert Wood Johnson Foundation; 2016. Accessed May 15, 2023. https://www.samhsa.gov/sites/default/files/programs_campaigns/childrens_mental_health/atc-whitepaper-040616.pdf
9. Corbin TJ, Purtle J, Rich LJ, et al. The prevalence of trauma and childhood adversity in an urban, hospital-based violence intervention program. *J Health Care Poor Underserved*. 2013;24(3):1021-1030. https://doi.org/10.1353/hpu.2013.0120
10. Binnie V, Le Brocque R, Jessup M, Johnston ANB. Adult frequent presentation to emergency departments and adverse childhood experiences: a scoping review. *Australas Emerg Care*. 2021;24(4):264-279. https://doi.org/10.1016/j.auec.2020.11.002
11. Fischer KR, Bakes KM, Corbin TJ, et al. Trauma-informed care for violently injured patients in the emergency department. *Ann Emerg Med*. 2019;73(2):193-202. https://doi.org/10.1016/j.annemergmed.2018.10.018

Copyright © 2023 Kung et al. This is an open-access publication distributed under the terms of the Creative Commons Attribution license.

12. Cole R. Reducing restraint use in a trauma center emergency room. *Nurs Clin North Am*. 2014;49(3):371-381. https://doi.org/10.1016/j.cnur.2014.05.010

13. Azeem MW, Aujla A, Rammerth M, Binsfeld G, Jones RB. Effectiveness of six core strategies based on trauma informed care in reducing seclusions and restraints at a child and adolescent psychiatric hospital. *J Child Adolesc Psychiatr Nurs*. 2011;24(1):11-15. https://doi.org/10.1111/j.1744-6171.2010.00262.x

14. Agboola IK, Coupet E Jr, Wong AH. "The coats that we can take off and the ones we can't": the role of trauma-informed care on race and bias during agitation in the emergency department. *Ann Emerg Med*. 2021;77(5):493-498. https://doi.org/10.1016/j.annemergmed.2020.11.021

15. Brown T, Ashworth H, Bass M, et al. Trauma-informed care interventions in emergency medicine: a systematic review. *West J Emerg Med*. 2022;23(3):334-344. https://doi.org/10.5811/westjem.2022.1.53674

16. Bruce MM, Kassam-Adams N, Rogers M, Anderson KM, Sluys KP, Richmond TS. Trauma providers' knowledge, views, and practice of trauma-informed care. *J Trauma Nurs*. 2018;25(2):131-138. https://doi.org/10.1097/JTN.0000000000000356

17. Matta G, Woodward-Kron RE, Petty S, Salzberg MR. Eliciting and responding to patient histories of abuse and trauma: challenges for medical education. *Med J Aust*. 2016;205(6):248-249. https://doi.org/10.5694/mja16.00216

18. Brown T, Mehta PK, Berman S, et al. A trauma-informed approach to the medical history: teaching trauma-informed communication skills to first-year medical and dental students. *MedEdPORTAL*. 2021;17:11160. https://doi.org/10.15766/mep_2374-8265.11160

19. Goldstein E, Murray-García J, Sciolla AF, Topitzes J. Medical students' perspectives on trauma-informed care training. *Perm J*. 2018;22(1):17-126. https://doi.org/10.7812/TPP/17-126

20. Elisseou S, Puranam S, Nandi M. A novel, trauma-informed physical examination curriculum for first-year medical students. *MedEdPORTAL*. 2019;15:10799. https://doi.org/10.15766/mep_2374-8265.10799

21. Stoklosa H, Lyman M, Bohnert C, Mittel O. Medical education and human trafficking: using simulation. *Med Educ Online*. 2017; 22(1):1412746. https://doi.org/10.1080/10872981.2017.1412746

22. Chokshi B, Walsh K, Dooley D, Falusi O, Deyton L, Beers L. Teaching trauma-informed care: a symposium for medical students. *MedEdPORTAL*. 2020;16:11061. https://doi.org/10.15766/mep_2374-8265.11061

23. So HY, Chen PP, Wong GKC, Chan TTN. Simulation in medical education. *J R Coll Physicians Edinb*. 2019;49(1):52-57. https://doi.org/10.4997/JRCPE.2019.112

24. Jones F, Passos-Neto CE, Braghiroli OFM. Simulation in medical education: brief history and methodology. *Princ Pract Clin Res*. 2015;1(2):46-54. https://doi.org/10.21801/ppcrj.2015.12.8

25. Lloyd MC, Ratner J, La Charite J, et al. Addressing trauma and building resilience in children and families: standardized patient cases for pediatric residents. *MedEdPORTAL*. 2021;17:11193. https://doi.org/10.15766/mep_2374-8265.11193

26. Miller-Cribbs J, Bragg JE, Isaacson M, et al. Evaluation of a simulation-based training program on childhood trauma with occupational therapy students. *J Occup Ther Educ*. 2021;5(2):9. https://doi.org/10.26681/jote.2021.050209

27. Grossman S, Berman S, Brown T, et al. Implementing a trauma-informed care curriculum in undergraduate medical education. In: Douglass LL, Threlkeld A, Merriweather LR, eds. *Trauma in Adult and Higher Education: Conversations and Critical Reflections*. Information Age Publishing; 2022:187-204.

28. Brown T, Berman S, McDaniel K, et al. Trauma-informed medical education (TIME): advancing curricular content and educational context. *Acad Med*. 2021;96(5):661-667. https://doi.org/10.1097/ACM.0000000000003587

29. Smidt A, Balandin S, Sigafoos J, Reed VA. The Kirkpatrick model: a useful tool for evaluating training outcomes. *J Intellect Dev Disabil*. 2009;34(3):266-274. https://doi.org/10.1080/13668250903093125

Received: June 28, 2022
Accepted: April 11, 2023
Published: July 28, 2023

Copyright © 2023 Kung et al. This is an open-access publication distributed under the terms of the Creative Commons Attribution license.
